# Natural drug sources for respiratory diseases from *Fritillaria*: chemical and biological analyses

**DOI:** 10.1186/s13020-021-00450-1

**Published:** 2021-05-31

**Authors:** Ye Wang, Hongping Hou, Qiang Ren, Haoyu Hu, Tiechui Yang, Xiwen Li

**Affiliations:** 1 Institute of Chinese Materia Medica, China Academy of Chinese Medical Sciences, No 16, Neinanxiao Street, Dongcheng District, Beijing, 100700 China; 2 Department of Pharmacy, Jining Medical University, Rizhao, 272000 China; 3 Nin Jiom Medicine Manufactory (Hong Kong) Limited, Hong Kong, 999077 China

**Keywords:** *Fritillaria*, Alkaloids, Chemical components, Ethnopharmacology, Respiratory disease

## Abstract

*Fritillaria* naturally grows in the temperate region of Northern Hemisphere and mainly distributes in Central Asia, Mediterranean region, and North America. The dried bulbs from a dozen species of this genus have been usually used as herbal medicine, named Beimu in China. Beimu had rich sources of phytochemicals and have extensively applied to respiratory diseases including coronavirus disease (COVID-19). *Fritillaria* species have alkaloids that act as the main active components that contribute multiple biological activities, including anti-tussive, expectorant, and anti-asthmatic effects, especially against certain respiratory diseases. Other compounds (terpenoids, steroidal saponins, and phenylpropanoids) have also been identified in species of *Fritillaria*. In this review, readers will discover a brief summary of traditional uses and a comprehensive description of the chemical profiles, biological properties, and analytical techniques used for quality control. In general, the detailed summary reveals 293 specialized metabolites that have been isolated and analyzed in *Fritillaria* species. This review may provide a scientific basis for the chemical ecology and metabolomics in which compound identification of certain species remains a limiting step.

**Supplementary Information:**

The online version contains supplementary material available at 10.1186/s13020-021-00450-1.

## Background

The global climate change increased the spread of respiratory diseases, resulting in a health-care burden to the human beings, especially in some developing countries. Respiratory diseases contain acute respiratory distress syndrome, chronic obstructive pulmonary disease (COPD), respiratory infections, etc. [1, 2]. More than 545 million individuals live with a chronic respiratory condition, which is the third leading cause of death following cardiovascular diseases and neoplasms; meanwhile, chronic diseases affected 7.4% of the world’s population in 2017 [3]. Coronavirus disease 19 (COVID-19) pandemic is a brachychronic respiratory distress syndrome with cough and fever symptoms, and it has caused more than 114 million confirmed cases and 2,534,520 confirmed deaths as of March 2021 [4]. Traditional Chinese medicine has been playing positive role for the cure and prevention of the epidemic [5, 6]; an example of such traditional medicine is Jinhua Qinggan Granule (comprising *Fritillaria thunbergii*, *Artemisia annua*, etc.) which exhibits a curative effect via attenuating cytokine storms, thus inhibiting the activity of severe acute respiratory syndrome coronavirus 2 and enhancing antiviral immunity [7, 8]. *F. thunbergii* is one of the main ingredients in Jinhua Qinggan Granule possessing anti-tussive and antiasthmatic properties. In addition, *Fritillaria* species, which are consumed as household medicines, have potential properties with rich pharmacological history.

The crude materials from *Fritillaria* were first showed in Shen Nong Ben Cao Jing, with efficacy of moistening dryness and clearing lung heat. Pharmacological studies transformed these traditional efficacies into scientific pharmacological activities, including anti-tussive, expectorant, anti-asthmatic effects because of the presence of alkaloids and other metabolites [9]. Alkaloids, the largest class of photochemical components of *Fritillaria* species and accounting for approximate 42.32% of all authenticated components, are regarded as potential agents that reduce lung injury induced in various ways [10]. Cough treatment using natural products from *Fritillaria* have significant advantages compared with the usual drugs, such as codeine, and display less or no side effects [11]. *F. cirrhosa*, *F. delavayi*, and *F. wabuensis*, and other five species, collectively named as “Chuan Bei Mu”, have evident ability to treat dry coughs without phlegm and chronic cough due to Yin deficiency [9, 12]. In addition, bulbs from *Fritillaria* process cold properties in terms of the theory of traditional Chinese medicine. These characteristics may be suitable for the symptoms caused by the COVID-19 virus.

Statistical analysis indicated that 1529 Chinese patent medicines contain crude materials consisting of *Fritillaria* species that are used as anti-tussive agents, accounting for 19.28% of all cough-related products. However, the destruction in wild resources and crude cultivation methods led to the imbalance between the supply and demand for producing these patent medicines, especially the alpine Himalayan species of *Fritillaria*, such as *F. cirrhosa* and *F. delavayi*. Furthermore, combined with these obstacles, the growth periods of 3–5 years resulted in high manufacturing cost, which extremely restricted the industrial application of *Fritillaria* species. Awareness of the export amount of herbal medicine made from *Fritillaria* increased from 195,700 kg in 2015 to 321,800 kg in 2019, whereas while the export value increased by 133% within 5 years. The commercial medicinal plant harvest supports 50–100% of households living at high elevations from 2700 to 3400 m [13]. Therefore, it is extremely necessary for the researchers to conduct the chemical and biological investigations, for which consider that the increasing demand for *Fritillaria* is leading to their over-collection in the market and decline in the wild [14].

Previous review articles focused on the chemical composition and molecular biological techniques [9], resource situation [15], breeding technology [16], and classification of *Fritillaria* species [17, 18]. Nevertheless, the scientific concern and broad research of the genus are lacking. A systematic and comprehensive review focusing on these contents should be conducted for in-depth studies of *Fritillaria* species to determine the possible correlation between their biological activities and metabolic profiles. In this report, we provide a critical summary of the detailed phytochemical and pharmacological analyses of crude extracts or authenticated components, traditional use, and botanical description of taxa belonging to *Fritillaria*.

## Botanical characterizations and traditional use

### Natural resource and botanical description

**Fig. 1:**
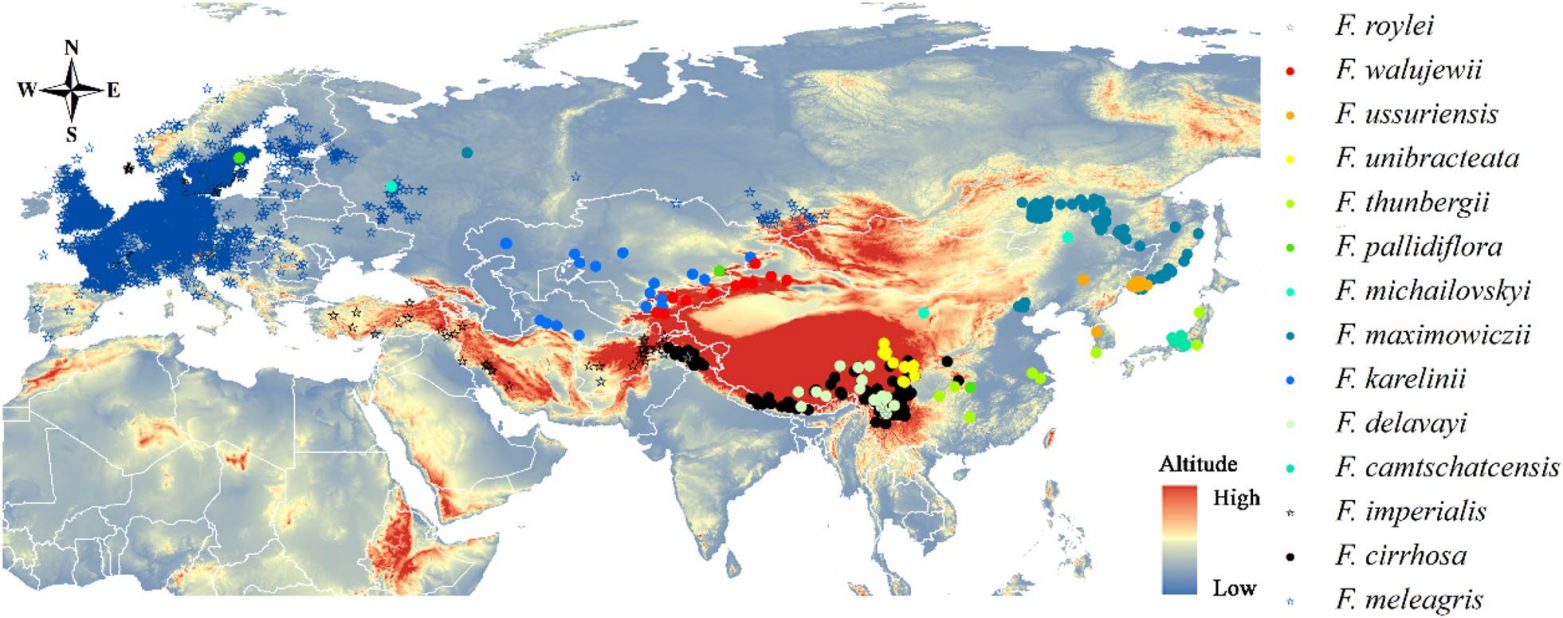
The distribution of main medicinal *Fritillaria* species in the Asian and Europe

In general, there are 130 species of *Fritillaria* (Liliaceae), cultivated or distributed in the world as some ornamental and medicinal plants, and the crude material or extracted components exists diverse phytochemical properties. Parts of species distributed in the Northern Hemisphere, and the high-altitude species (such as *F. cirrhosa* and *F. delavayi*) alongside the Himalayan Mountain have been the most intensively exploited (Fig. 1). The extensive demand of *Fritillaria* species resulted in a situation that they are regarded as a Class III protected species; especially, their bulbs are registered as an active ingredient in medicinal preparations in Oceania, North America, and Asia [19]. The IUCN Red List of Threatened Species contains eighteen *Fritillaria* species and the document is an accessible system used for determining *Fritillaria* species as global extinction, because these plants are vulnerable to unsustainable collection after their bulbs have been dug out. Nine *Fritillaria* species are traded in Europe, which are commonly harvested from China with high volume of business transactions. *F. roylei* is included in the Ayurvedic Pharmacopeia, whereas the Korean Pharmacopoeia contain four species of the genus within its “Fritillariae Cirrhosae Bulbus” monograph. Meanwhile, Chinese Pharmacopeia contains ten species and one cultivation variation of *Fritillaria* from the genus used for different medicinal properties.

The medicinal plants of *Fritillaria* species are generally characterized as perennial herbs with 2–3 fleshy and farinaceous (after dried) bulbs covering a translucent tunic. The stems are erect and without branches with petiolate basal and sessile cauline leaves. The arrangement of leaves is spirally alternate, opposite, or whorled, and the leaf blade is oblong or lanceolate. One or several flowers form a racemose or umbellate inflorescence, and the bracts are present after fading. The bisexual flowers are usually nodding with a campanulate or saucer-shape tessellation, with dark and light-color spots, and a nectary near the base adaxially. Six free stamens, and the anthers of stamens are basifixed, rarely dorsifixed. The three-lobed or subentire stigma is linear or extremely short. The winged or wingless capsule is erect with three locules and six angles. The seeds, which are generated from loculicidal dried fruits, are flatly arranged in two rows in each valve.

The Plant List, as an authoritative working list guided by the 2002–2010 Global Strategy for Plant Conservation [20], mentions 551 scientific plant names of *Fritillaria*. 156 scientific records are accepted as species names among of them, which indicates vast potential for the development of the *Fritillaria*. The Flora of China subdivided *Fritillaria* into three sections (Sect. *Fritillaria*, Sect. *Liliorhiza*, and Sect. *Theresia*) based on morphological traits. Most of the collected species in this review, such as *F. cirrhosa*, *F. delavayi*, and *F. hupehensis*, belong to Sect. *Fritillaria*. One species, *F. maximowiczii*, belongs to Sect. *Liliorhiza*, whereas *F. karelinii* belongs to Sect. *Theresia*. To date, several species have been successfully cultivated by herbal farmers in China, inspired by the vast demand and high profits. The main cultivated species include, *F. cirrhosa*, *F. unibracteata*, *F. thunbergii*, and *F. ussuriensis*, all of which belong to the Sect. *Fritillaria*. However, the whole cultivation period from seeds to commercial forms lasts for at latest four years, which seriously restricts the deep resource utilization and causes the destruction of wild *Fritillaria* resources, especially the original plants of Chuan Bei Mu. The plant grows one leaf and a weeny bulb within the first two years. The mature plants form fertile flowers and fruits after a five-year cultivation period. Bulbs are the confirmed the standard for medicinal purpose.

### Ethnopharmacological properties

The dried bulbs of *Fritillaria* species (Bei Mu in Chinese) are consumed as an anti-tussive agent. The first record of the plant emerged in Wan Wu, an ancient herb book, under the name of Bei Mu, under without detailed botanical description, in the dynasty of Chunqiu Zhanguo Dynasty (BC 770–BC 221), followed by Shen Nong Ben Cao Jing between BC 221 and BC 202 [21]. The tuber of *Bolbostemma paniculatum* (Maxim) Frank. is used as Bei Mu and named as Tu Bei Mu. Two kinds of Bei Mu existed, namely, the crude materials Zhe Bei Mu and Tu Bei Mu with the clinical usage as Chinese medicine from AD 220 to AD 498; they were recorded in Ben Cao Jing Ji Zhu and Ming Yi Bie Lu [21]. The original plants were authenticated as species of *Fritillaria* in Xin Xiu Ben Cao and Ben Cao Tu Jing, which are two official herbal books. These Bei Mu materials were divided into Chuan Bei Mu and Zhe Bei Mu based on their geographical origins in Ben Cao Hui Yan in AD 1624 [21]. Ben Cao Cong Xin contains the morphological traits observed in Qing Dynasty (AD 1757), in which the base of the bulbs from Sichuan Province were flat-bottomed, whereas those produced from Zhejiang Province of China were the biggest [21]. In general, these original plants of Bei Mu were gradually defined with the development of plant taxonomy, and the conception of Bei Mu material and origin of *Fritillaria* species, have been formed since Ming Dynasty.

Nine *Fritillaria* species native to China are largely employed against several ailments in 11 national minorities, such as Tibetan, Mongolian, Miao, Lisu, Tujia, Miao, Kazakh, Uighur, Jingpo, De’ang, and Korean, with ethno-pharmacological reports from traditional practitioners. The bulbs, leaves, and seeds are equally utilized as herbal medicine in the Tibetan national minority, in which the bulbs are used for curing tracheitis and menometrorrhagia. The leaves are consumed for impetigo, and the seeds are effective remedy for the head and heat deficiency symptoms. The other 10 national minorities use the bulbs as a medicinal component given its different pharmacological properties; management of cough and phlegm is the main observation among these records of national minorities in China (Additional file 1: Table S1). In addition to China, other endemic species of *Fritillaria* contain similar alkaloids components, which may have similar pharmacological activities. *F. michailovskyi* is an endemic species in Turkey contain common steroidal alkaloids [22]. Additional file 1: Table S2 summarizes the traditional or ethnopharmacological records of the species included in this review. Coughs, asthma, and bronchitis are the main efficacies observed not only in Chinese Bei Mu but other congeneric species in other countries.

The medicinal potential of *Fritillaria* in the national minorities of China or other countries may present huge development capability in modern pharmacological research, especially certain high-altitude species threatened with extinction, which is caused by expanding human activities and habitat degradation. The further research of these species will provide scientific explanation for their traditional efficacy and be beneficial for the economic development of the regions.

## Structure and properties of phytochemicals in *Fritillaria* species

*Fritillaria* is rich in multiple secondary metabolites, whereas the alkaloids are the main compounds isolated and identified from the crude extracts in the bulbs [12], in addition to terpenoids, steroidal saponins, and phenylpropanoids. These extracted and authenticated constituents are summarized in Additional file 1: Tables S3–S9 and the representatives are illustrated with their chemical structure.

### Alkaloids

**Fig. 2:**
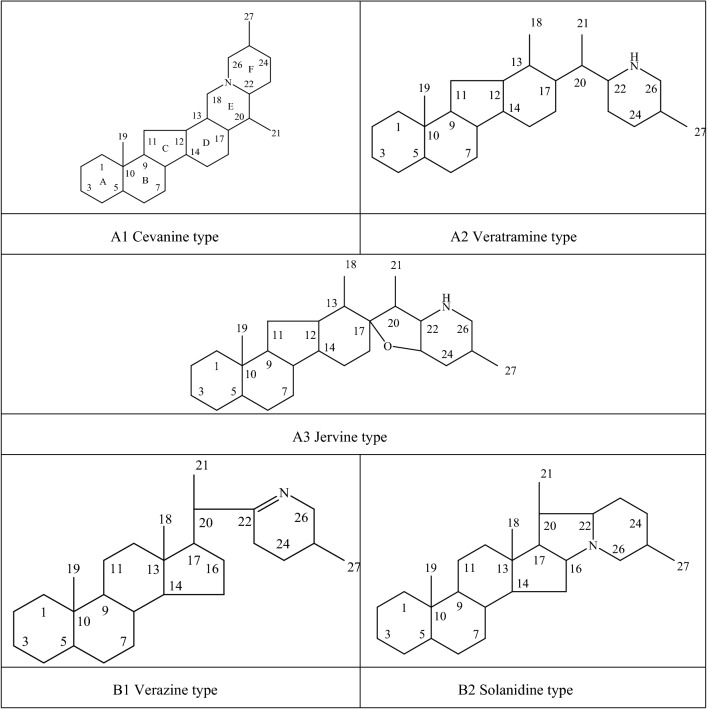
Basic skeletons of *Fritillaria* steroidal alkaloids

**Fig. 3:**
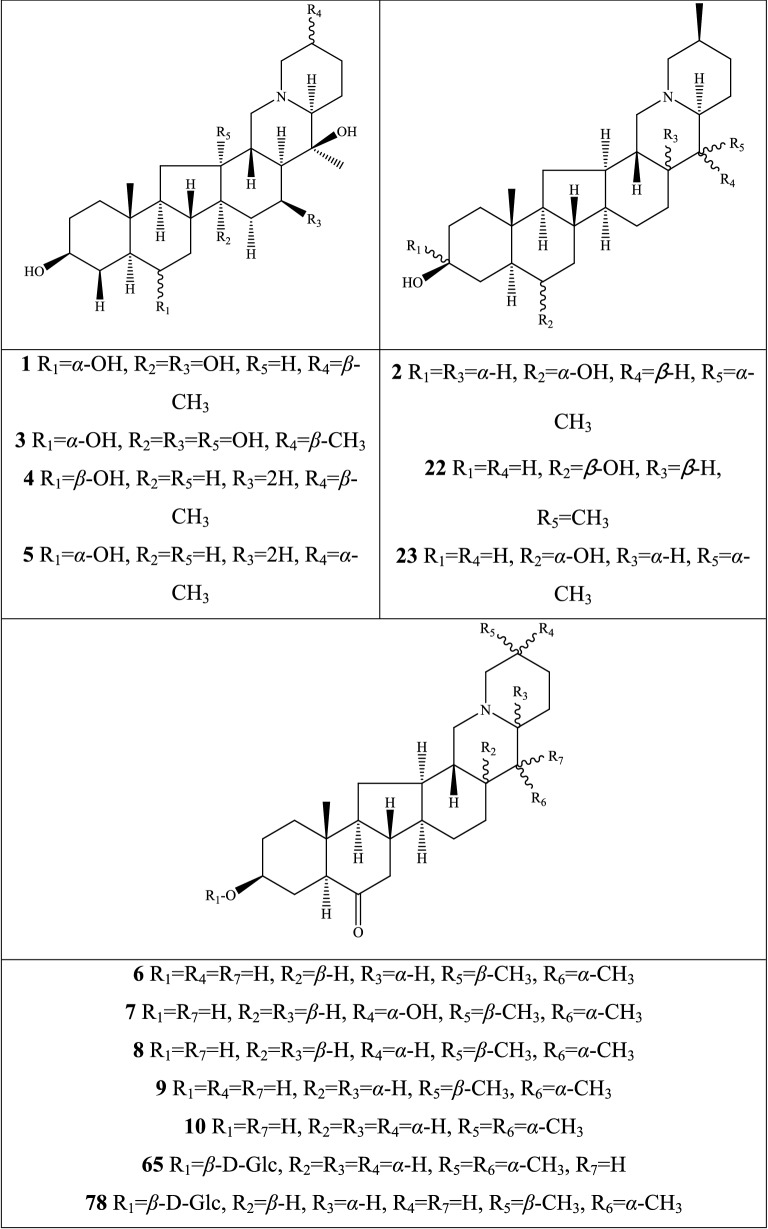

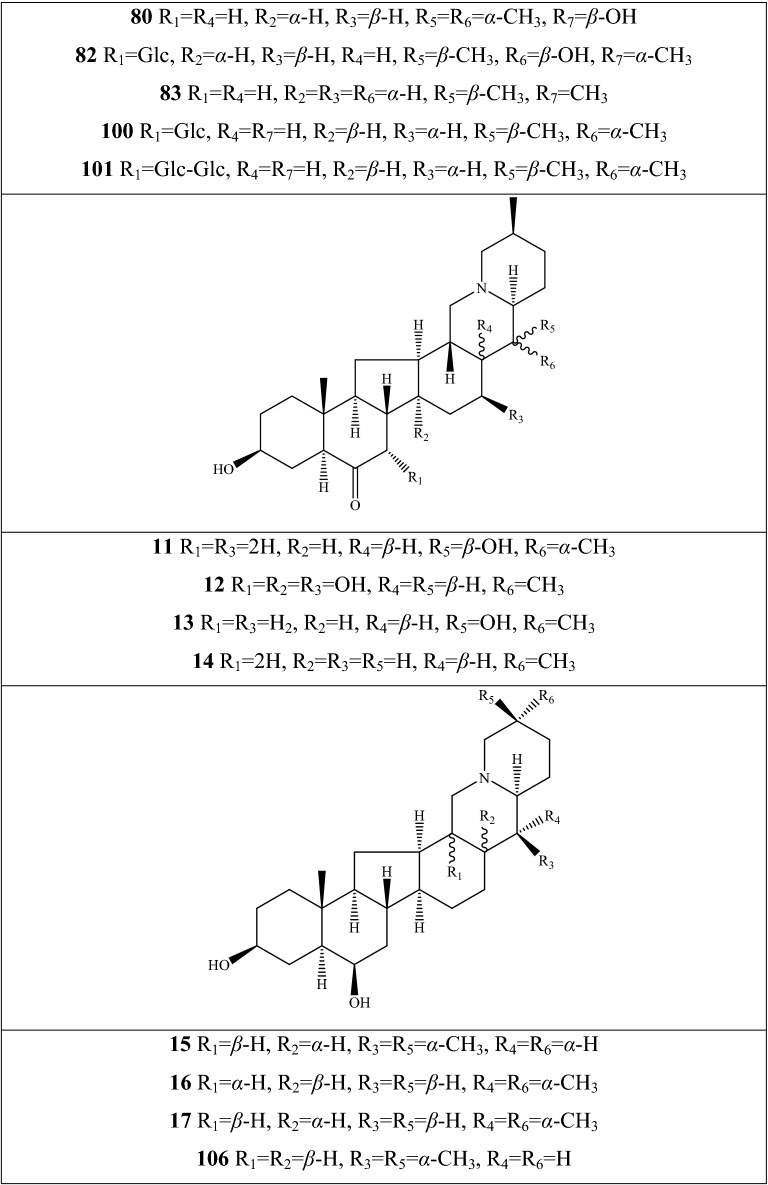

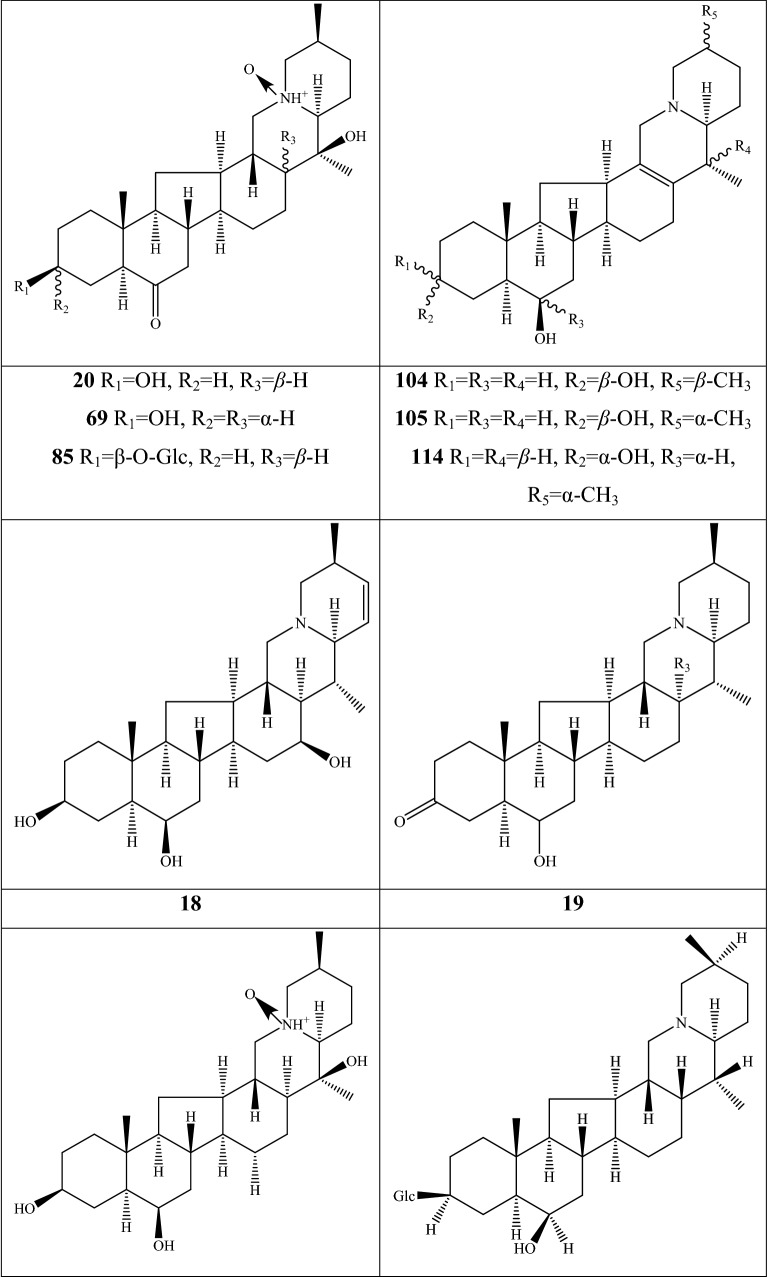

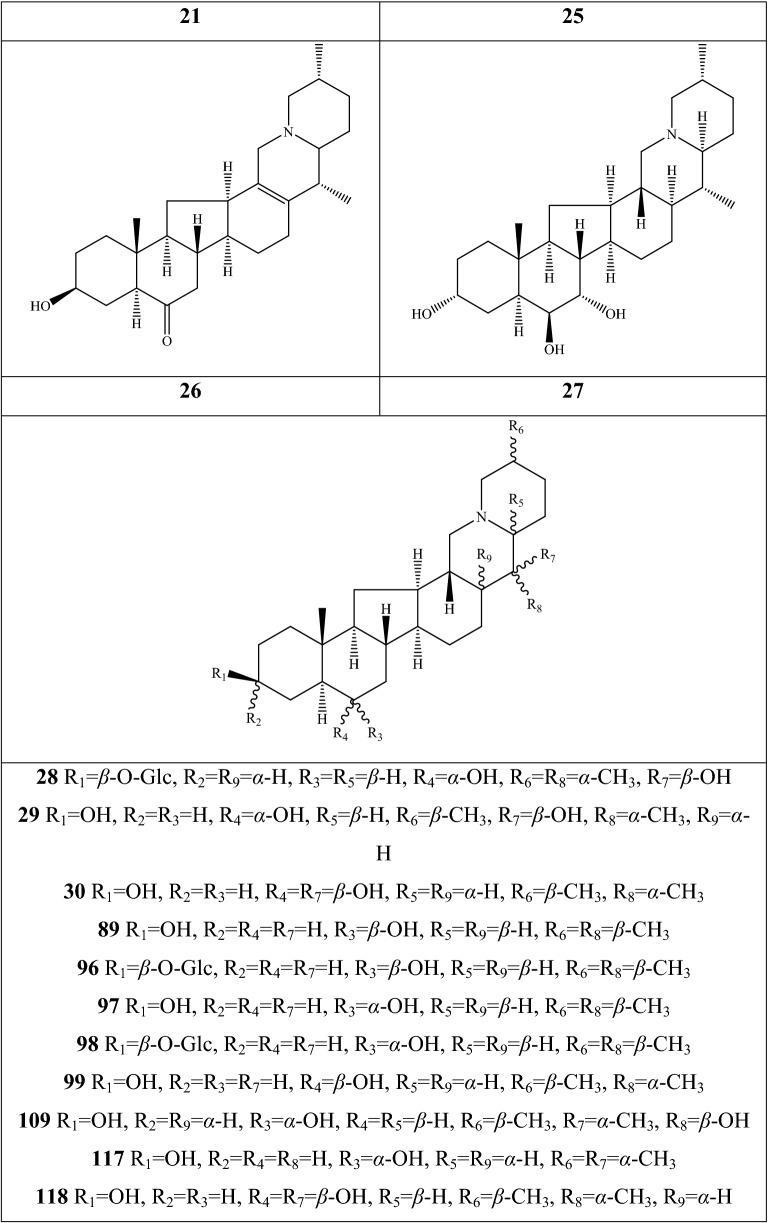

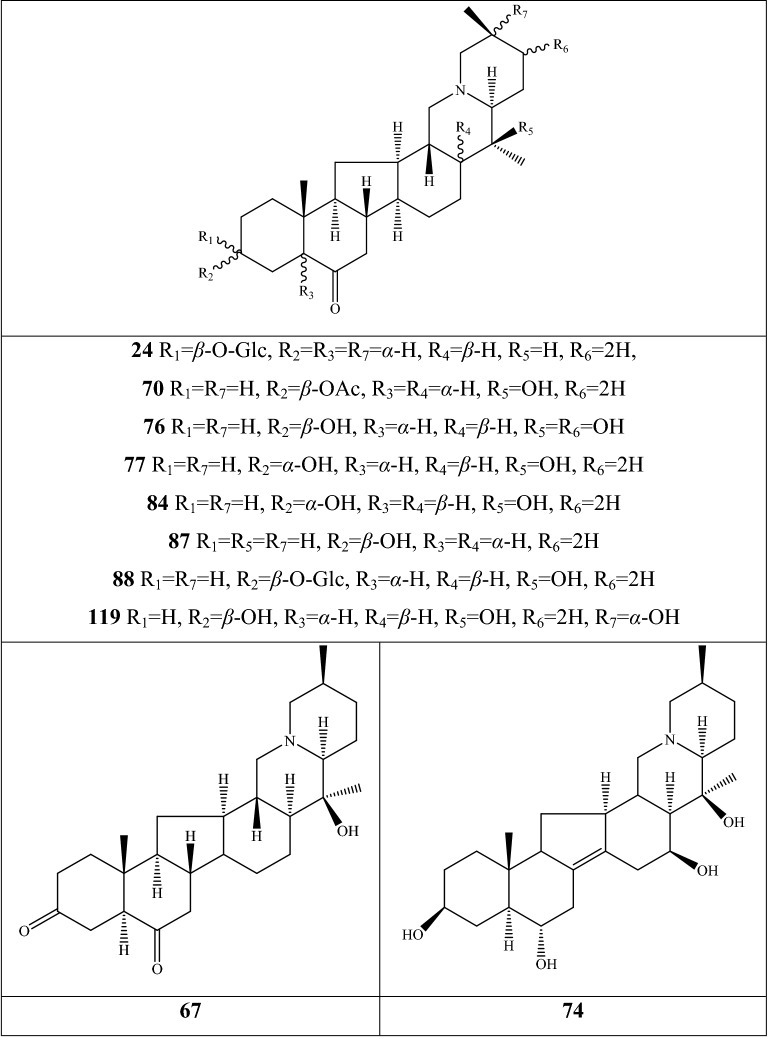

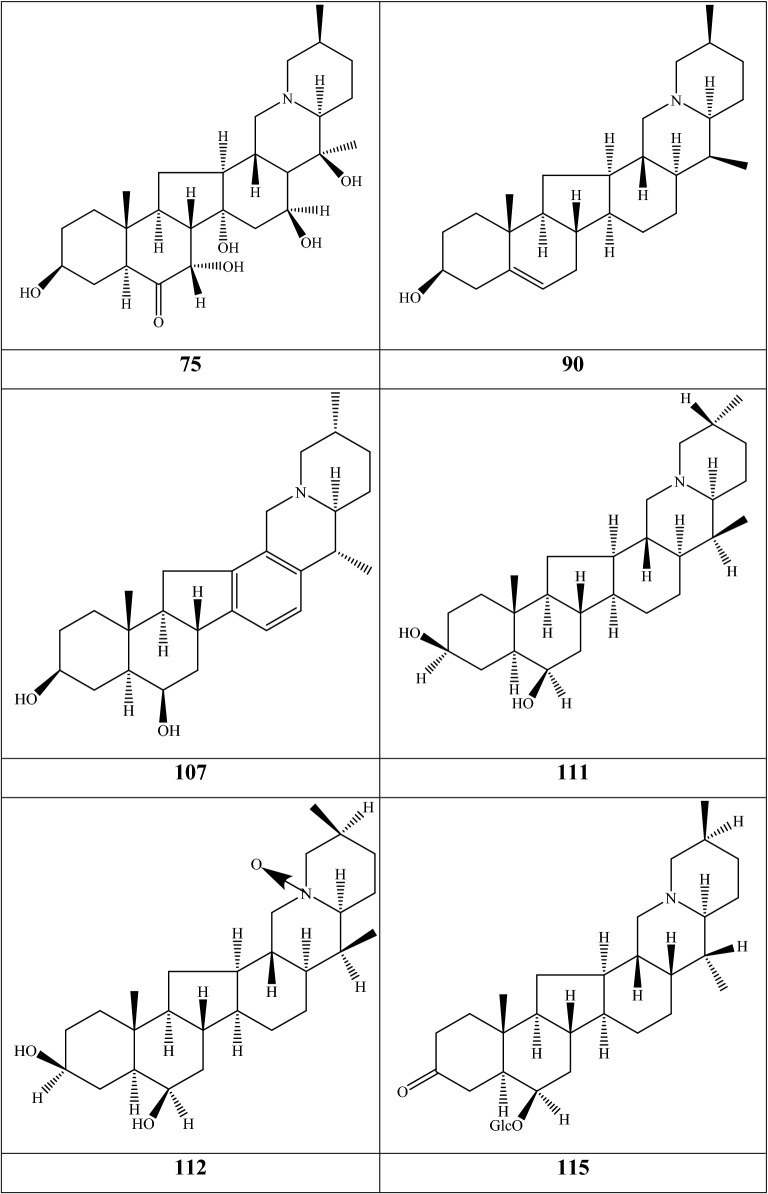

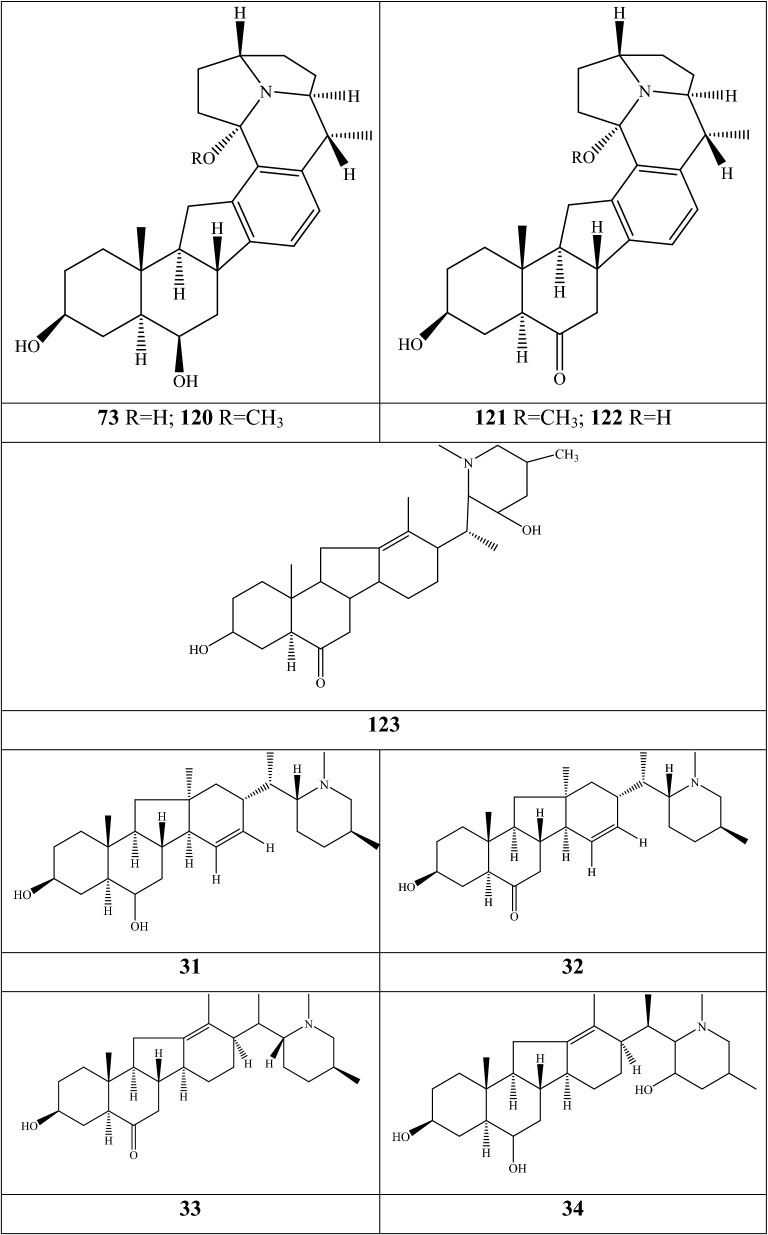

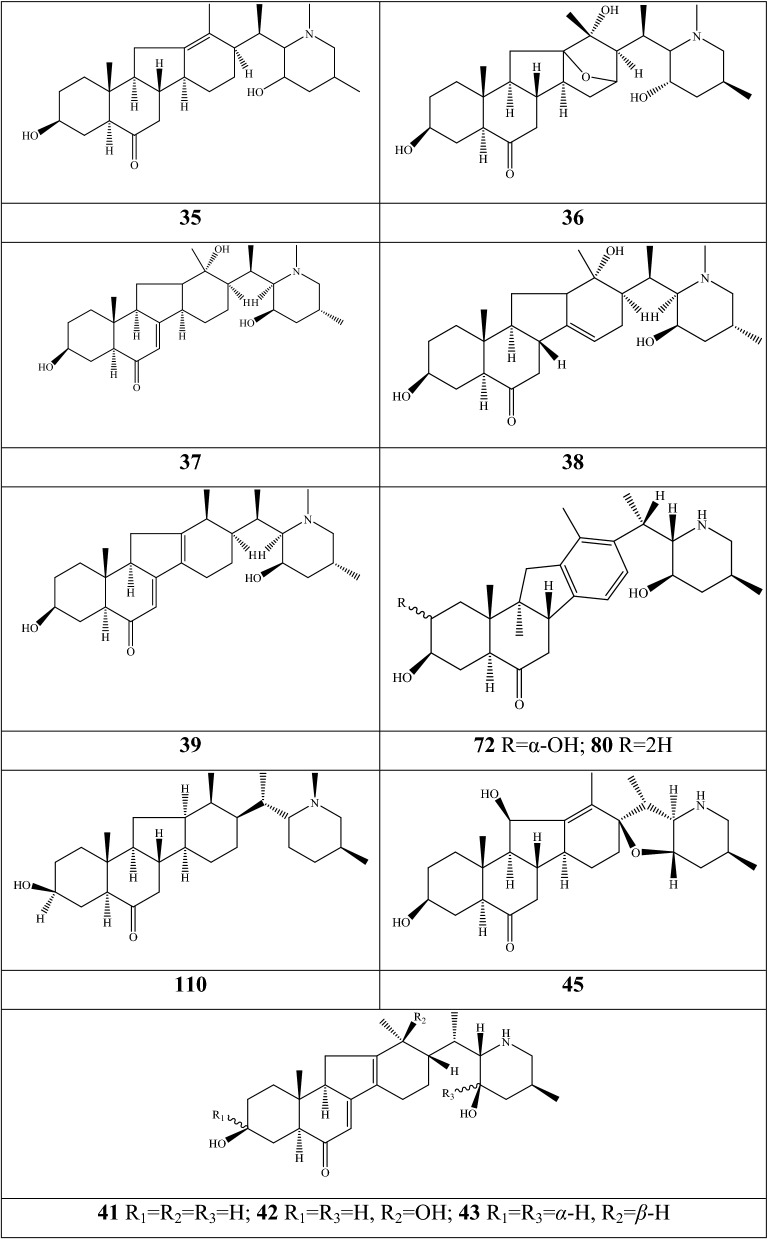

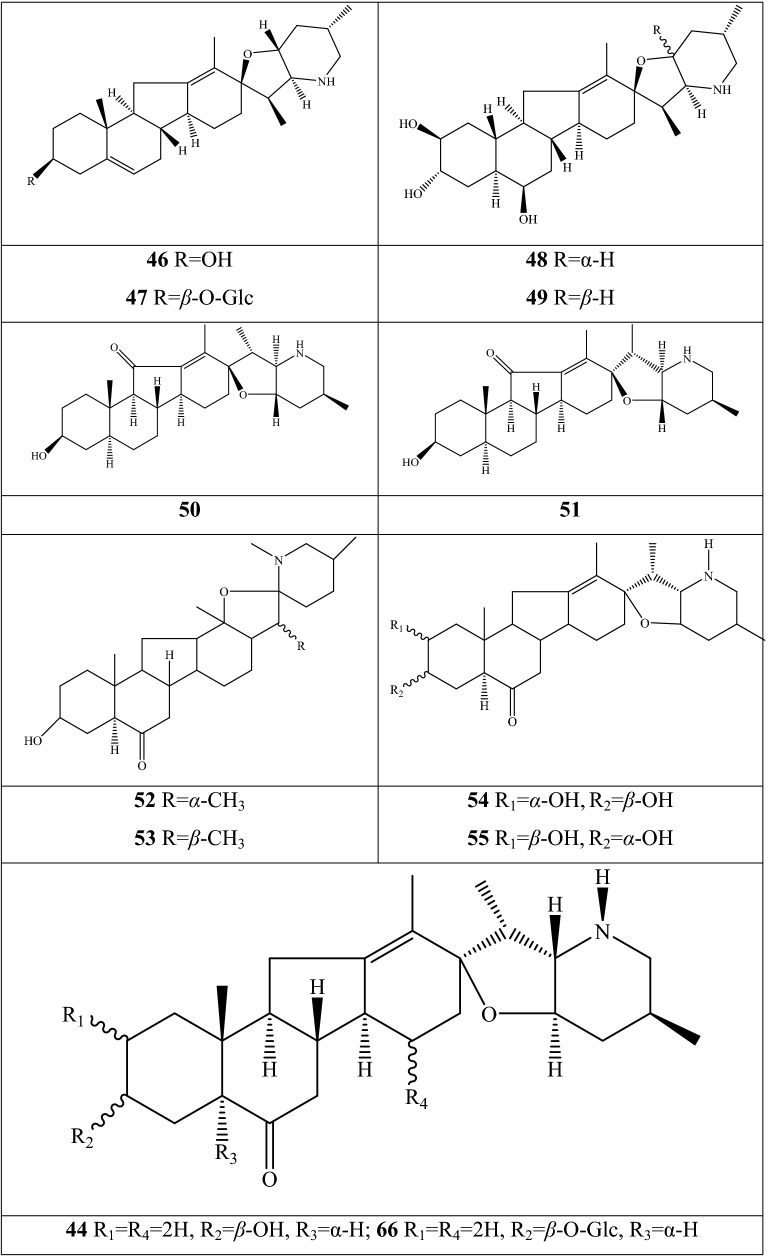

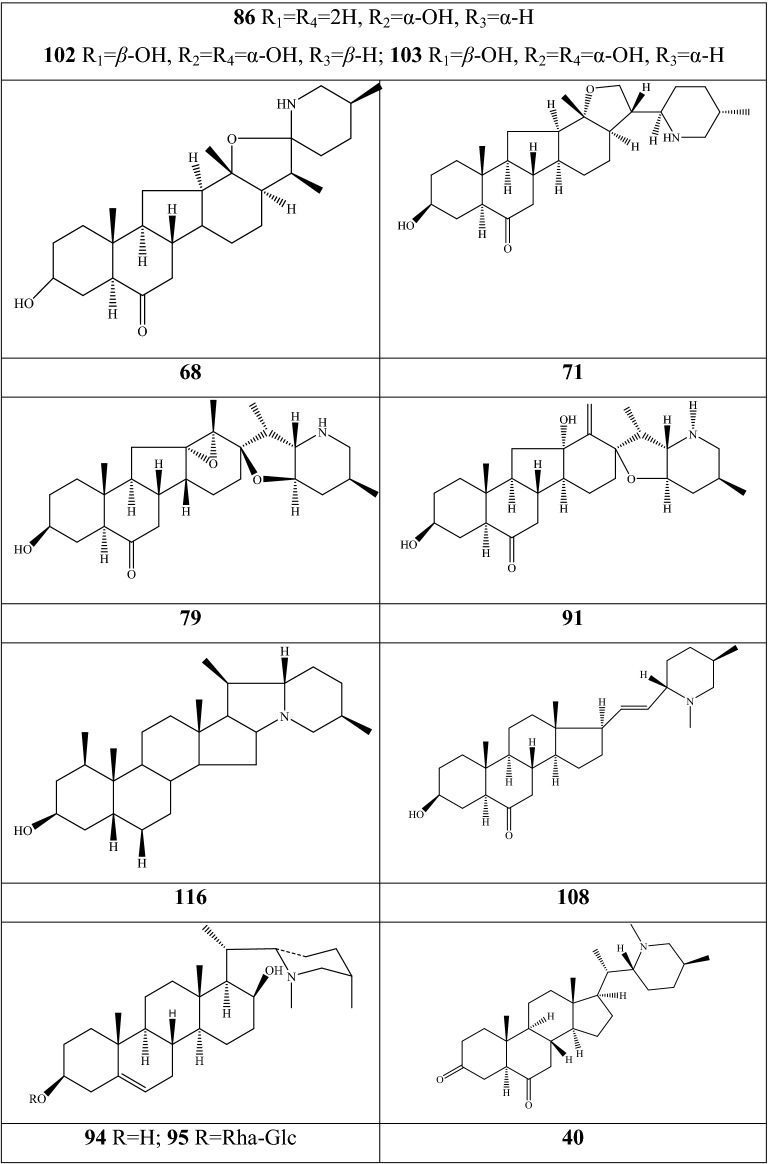

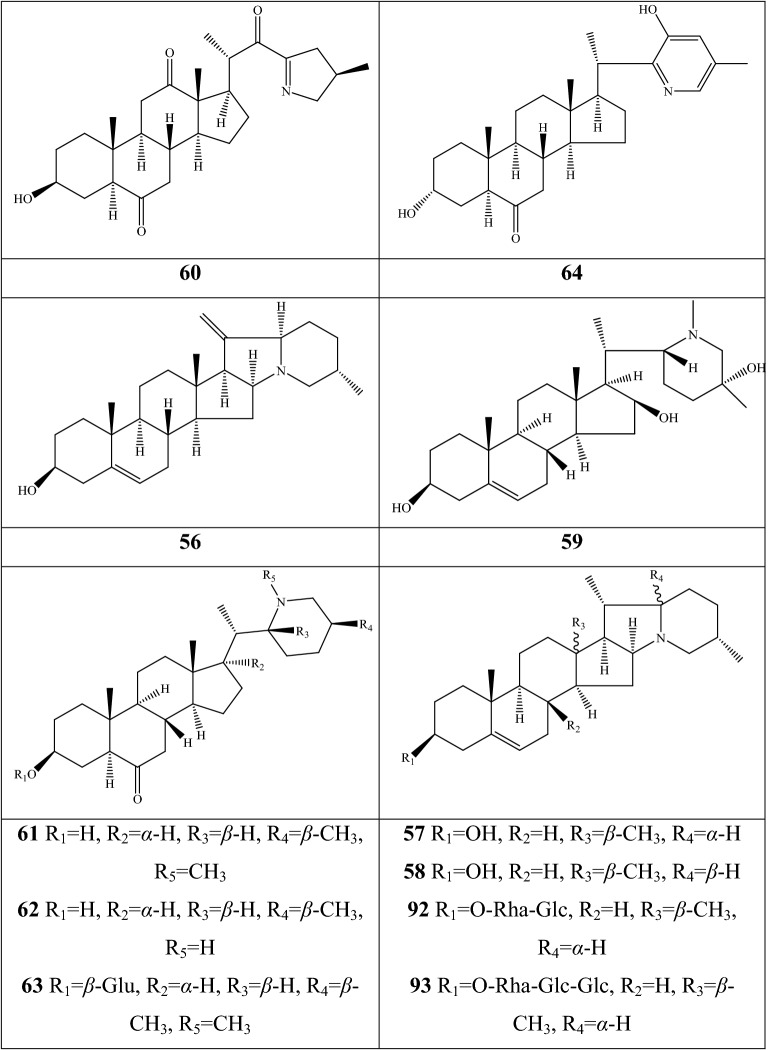

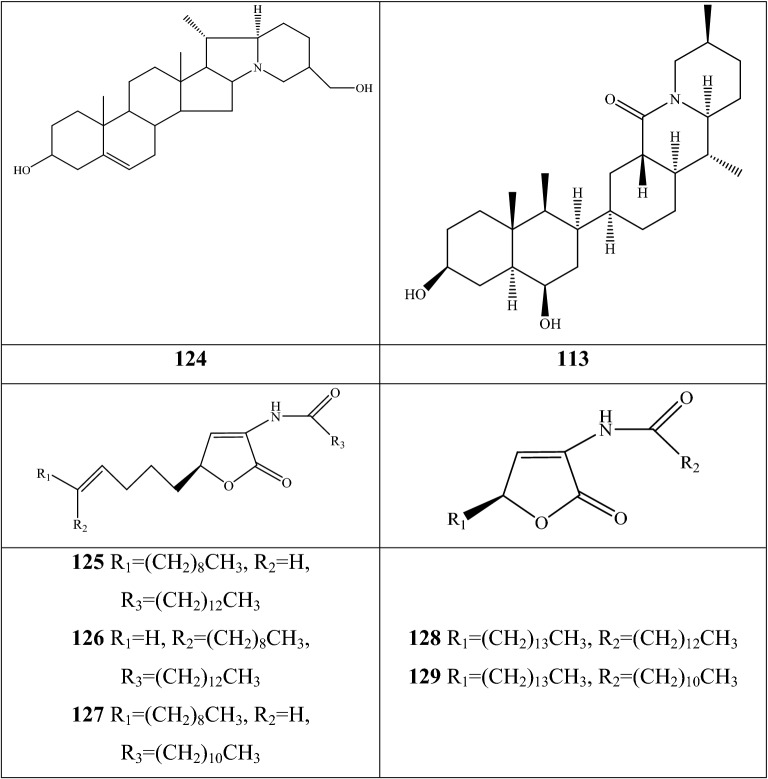
Structures of alkaloids in *Fritillaria* species

Alkaloids are organic components characterized by the basic nitrogen atoms. Based on the carbon framework, possessing a C_27_ cholestane carbon skeleton with carbocyclic and heterocyclic rings, the alkaloids extracted from *Fritillaria* species mainly consist of isosteroidal and steroidal types [12]. The isosteroidal alkaloids (*Veratrum* steroids), are further divided into cevanine (A1), veratramine (A2), and jervine (A3) types according to the linkage patterns between the E and F rings (Fig. 3). These alkaloids have a five-membered carbocycle. Meanwhile, *Solanum* steroidal alkaloids (comprise of solanidine B1 and verazine B2 type), which the nitrogen atom of the former is at the indolizidine ring, and that of the latter is in the piperidine ring (Fig. 2). Alkaloids have a six-membered carbocycle, a peculiar chemo-taxonomical significance, and are regarded as valuable markers in Liliaceae. The detailed chemical structures of 129 alkaloids (**1**–**129**) is shown in Fig. 3, and Additional file 1: Table S3 displays their occurrence in single taxa and botanical parts along with their trivial or semi-systematic names.

#### Isosteroidal alkaloids

More than 100 isosteroidal alkaloids, which account for 85% of the total alkaloids, have been isolated from the complex blend of secondary metabolites in different botanical parts of *Fritillaria* species. The results indicate that isosteroidal alkaloids are the main phytochemical profiles in the genus. Four chemical components (peimine **2**, sipeimine **11**, peiminine **13,** and peimisine **44**) exist in more than 10 *Fritillaria* species, and these four constituents can be regarded as indexes of the genus. The combination of peimine and peiminine is used as an evaluation index of Zhe Bei Mu, whereas peiminine is regarded as a key component for the quality control of Ping Bei Mu and Hu Bei Bei Mu, with different content criteria indicated in the Chinese Pharmacopeia. Verticinone 13 is easily oxidized from verticine, and the synthesized cholic acid-verticinone ester showed more potent activity than codeine phosphate [23]. Sipeimine is used for the quality assessment of Chuan Bei Mu, whereas the contents of the component combined with sipeimine-3β-d-Glc is an analytical marker for Yi Bei Mu. Three of the four index components are cevanine-type isosteroidal alkaloids.

A total of 24 species and 3 varieties (*F. taipaiensis* var. *ningxiaensis, F. unibracteata* var*. wabuensis*, and *F. ebeiensis* var. *purpurea*) contain cevanine-type isosteroidal alkaloids (Additional file 1: Table S1). These types of constituents have a *trans* configuration between that of A and B rings, except shinonomenine **90** (no hydrogen at the C_5_ position) [24] and fritirorine A **84** (*cis* configuration) [25] that are isolated from the bulbs of *F. tortifolia*, an endemic herb in the Xinjiang Province of China. The configuration of all cevanine-type isosteroidal alkaloids, which is currently authenticated currently between B and C, is the same as that of A and B rings except for benzo(7,8) fluoreno(2,1-*β*)quinolizine cevane-3,6,16,20-tetrol **74** in the bulbs of *F. ussuriensis* [26]. Inversely, rings C and D have *cis* configuration as long as hydrogen exists at C_12_ and C_14_, respectively. Six components (ussuriedine **73** and **74**, heilonine **107**, ussurienine **120**, ussurienone **121**, and ussuriedinone **122**) with saturated bonds exist between C_12_ and C_14_, and they are mostly extracted from *F. ussuriensis*, a cultivated species in Northeast China. Moreover, the steric configuration of methyl at the C_19_, C_21,_ and C_27_ positions varies with different constituents. C_3_ and C_6_ are common positions with a hydroxide radical or carbonyl, whereas the hydroxide radical at the C_3_ position is often connected with a glucopyranosyl, such as that in hupeheninoside **24** and zhebeininoside **28**, or multiple molecules, such as that walujewine E **101** that is only found in *F. walujewii*, which is distributed in the Xinjiang Uygur Autonomous Region of China [27]. 3-*O*-acetoxyverticinone **70**, as a new isosteroidal cevan-based alkaloid, was firs extracted from the bulbs of *F. hupehensis*, it contains a carboxyl connected to the hydroxide radical at the C_3_ position [28]. Additionally, three isosteroidal alkaloids, including hupehenizine **19**, verticinedinone **67**, and yubeiside **115**, were isolated and authenticated from *F. imperialis*, *F. anhuiensis*, and *F. yuminensis*, respectively, with a carbonyl at the C_3_ position [29–31]. Imperialine-N-oxide **20**, a nitric oxide, was the first chemical component extracted from the natural herbs of *F. pallidiflora* [32], followed by the isolation and authentication of four other similar constituents, namely, isoverticine-*β*-*N*-oxide **21** from *F. unibracteata* var. *wabuensis* [33], verticinone-*N*-oxide **69** from *F. shuchengensis* [34], and lichuanisinine **112** from *F. lichuanensis* [35]. Hydroxide radical and methyl are common substituents at the C_21_ position.

Bulbs from seven *Fritillaria* species were subjected to isolation and authentication research, Veratramine-type isosteroidal alkaloids from the bulbs have been reported. The type of alkaloids is characterized by the absence of ring E, a common aromatic ring D, and piperidine ring F. Hydroxide radical (*β*-OH) at the C_3_ position is the common characteristic and with a *trans*-configuration between A and B rings, similar to cevanine-type those. Most of these alkaloids types have *trans* configuration between B and C rings except for certain components without hydrogen at the C_8_ position, such as puqienine C **37** and puqienine E **39** isolated from the bulbs of *F. puqiensis* [36]. Impranine **31** and dihydroimpranine **32**, as new C-nor-D-homo alkaloids, were first and individually authenticated from *F. imperialis*, which is used for various ailments in Turkish folklore [37]. Puqienine F **36** is a novel veratramine alkaloid with a 12,16-epoxy ring, was authenticated from *F. puqiensis* and elucidated by X-ray crystallographic analyses [38]. Suchengbeisine **71** is the only veratramine alkaloid containing an epoxide ring adjacent to ring D; it was elucidated as (22R,25R)-13α,21-epoxy-5,6,12,13-tetrahydro-3β-hydroxy-5α-veratraman-6-one from *F. shuchengensis* in Anhui Province, China by intensive spectroscopic methods [34]. The type of alkaloids is species-dependent, and a specific constituent is found in a certain species.

Jervine-type alkaloids were isolated and identified from nineteen *Fritillaria* species with four botanical parts. Peimisine **44** and hupehenisine **50** were discovered from the aerial parts of *F. ussuriensis* and *F. hupehensis*, respectively. Other constituents of this alkaloid type were authenticated from the bulbs. The alkaloid types consist of hexacyclic components and a furan ring between C_17_ and C_22_, which form a bridge for the piperidine ring F and a six-membered ring D. Hydroxide radical commonly exists at the C_3_ position of ring A forming a trans the configuration between rings A, B, and C, which is similar to other isosteroidal alkaloids. Cycloposine **47** and peimisine-3-*O*-β-d-glucopyranoside **66** were isolated from *F. pallidiflora* [32], *F. unibracteata* [39], and *F. yuminensis* [40], in which the hydroxide radical was connected with glucopyranosyl. Yibeinone A **79** is a rare jervine alkaloid with 12α,13α-epoxy ring from *F. pallidiflora* authenticated as 22,26-imino-17,23-oxido-5α-jerv-6-oxo-3β,14β-diol-12α,13α-epoxy, [41]. Walujewine A **91** was isolated from the bulbs of *F. walujewii*, which is distributed in Xinjiang Province, China, it contains one olefinic carbon at the C_18_ position, which extremely differs from that of other jervine alkaloids [27].

#### Steroidal alkaloids

Verazine alkaloids have been extracted from six *Fritillaria* species and characterized as the steroidal skeleton combined with 22/23,26-epiminocholestane heterocyclic skeleton. Puqietinedione **40** [36], as a new steroidal alkaloid, was first isolated from the bulbs of *F. puqiensis*, whereas three other similar components, including puqietinone **61** [42], *N*-demethylpuqietinone **62**, and puqietinonoside **63** [43], were extracted from the same species. Hapepunine **94** and its glycosidal form (hapepunine-3-*O*-α-l-rhamnopyranosyl-(1 → 2)-β-d-glucopyranoside **95**) are newly discovered natural compounds that were isolated together from the aerial parts of *F. thunbergii* [44]. Similar to the extraction parts, pingbeinine **59** was discovered in the stems and leaves of *F. pallidiflora*, it has an α-orientation hydroxy at C_27_ and *β*-OH at C_15_ [45]. Delavidine **60** [46], ferisinine **64** [37]**,** and michainine **108** [22] exist in *F. delavayi*, *F. imperialis*, and *F. michailovskyi*, respectively. The latter two species are endemic species in Turkey.

Solanidine has a hexacyclic carbon framework comprising of a base steroidal skeleton and an indolizidine ring. Eight solanidine alkaloids were discovered from five *Fritillaria* species. Solanidine **57** and its glycoalkaloids (**92** and **93**) were discovered in *F. thunbergii*, *F. yuminensis*, and *F. cirrhosa*, which are three native species of China. Component **58** were the initial solanidine alkaloids with the 22-S configuration discovered in *F. anhuiensis*, isolated from natural materials [47].

High percentage of these chemical components were isolated from the bulbs part of nearly 30 species summarized in the review. An interesting finding showed that peimine, as an index component, is distributed in four botanical parts of *F. ussuriensis*, which implies that the non-medicinal parts may be a potential resource for industrial extraction [26, 45, 48, 49]. There is possible relationship between commercial potential and research frequency focusing on chemical components. Eleven *Fritillaria* species have been recorded in the Chinese Pharmacopeia, in which 9/11 (except for *F. przewalskii* and *F. taipaiensis*) were subjected to isolation and authentication of chemical components. The crude herbs, including Zhe Bei Mu and Yi Bei Mu are the most popular research objectives in the study of Bei Mu. The research focuses on the collection of both Bei Mu materials from certain Himalayan medicinal species or other high-altitude species, several of which are registered as proprietary medicines with a cross-border trade among China, Nepal, Australia, Canada, and Singapore [19]. Moreover, the narrow altitude range of several species is a restraining factor resulting in high-profit Bei Mu materials and expensive herbs that are popular research objectives [50].

#### Amide alkaloids

The current analytical strategy confirmed the presence of five amide alkaloids (fritenolide A–E **125**–**129**), and their occurrence was reported for the first time in *Fritillaria* species. The target species, *F. unibracteata*, is one of type of Chuan Bei Mu, and which enriched the types of alkaloids as unusual α-amino butenolides only recorded in marine organisms and fungus before [51].

### Terpenoids

**Fig. 4:**
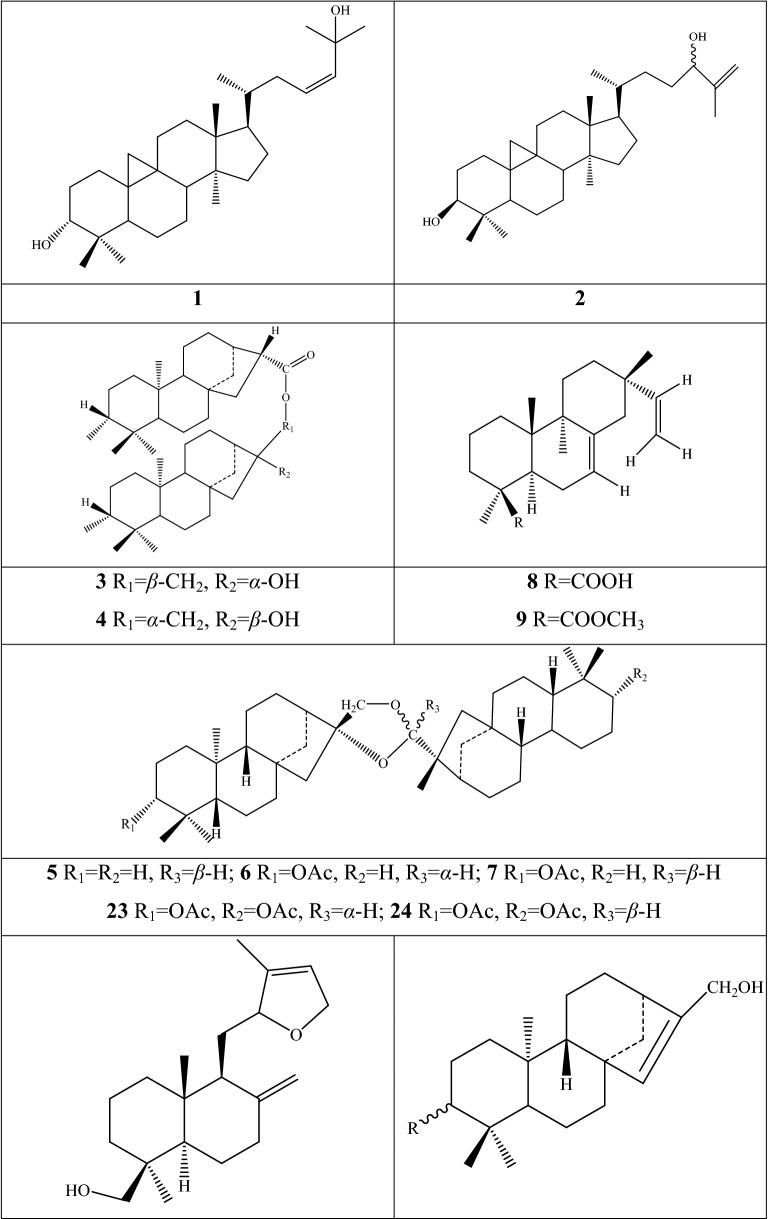

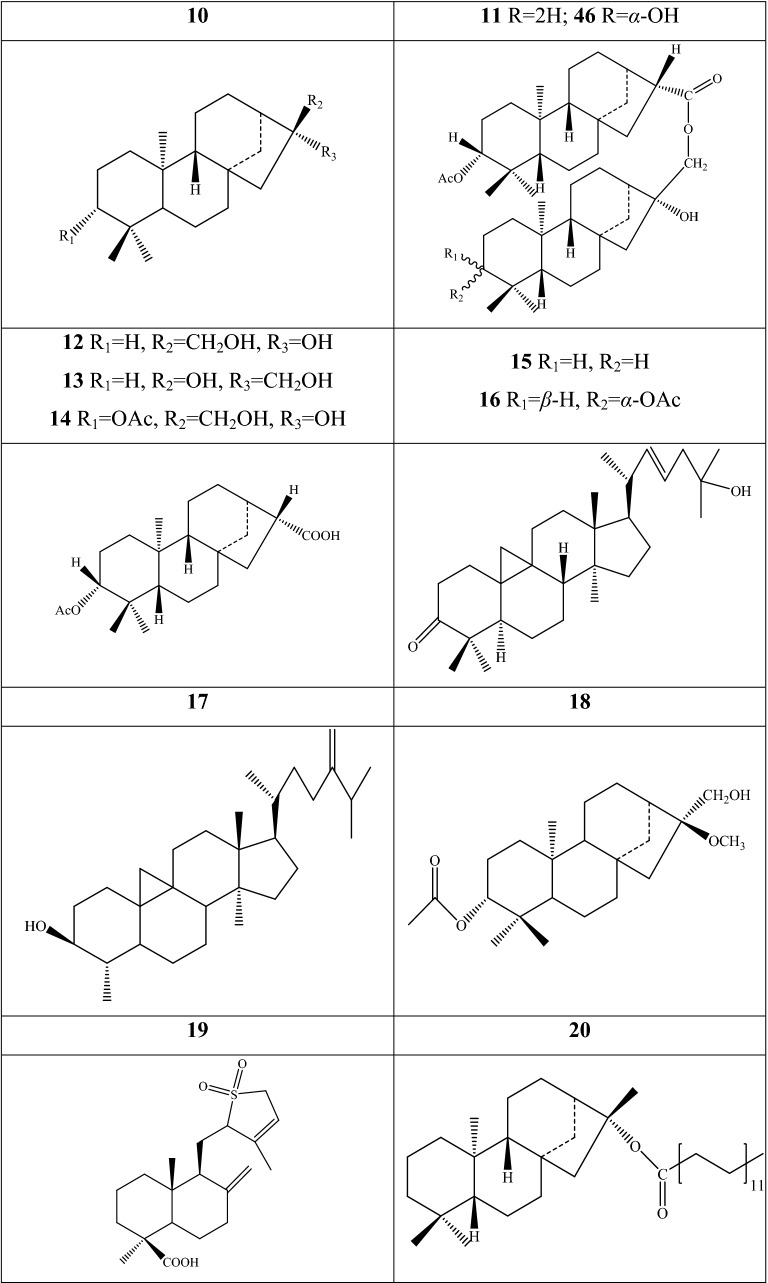

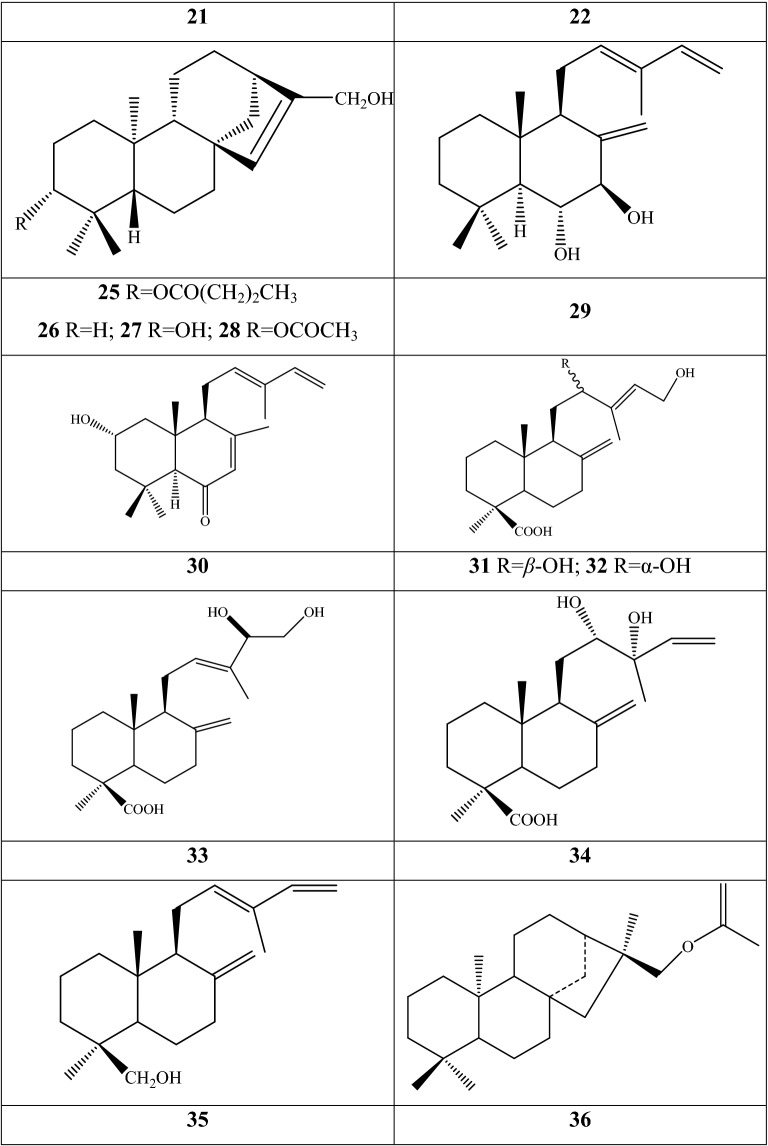

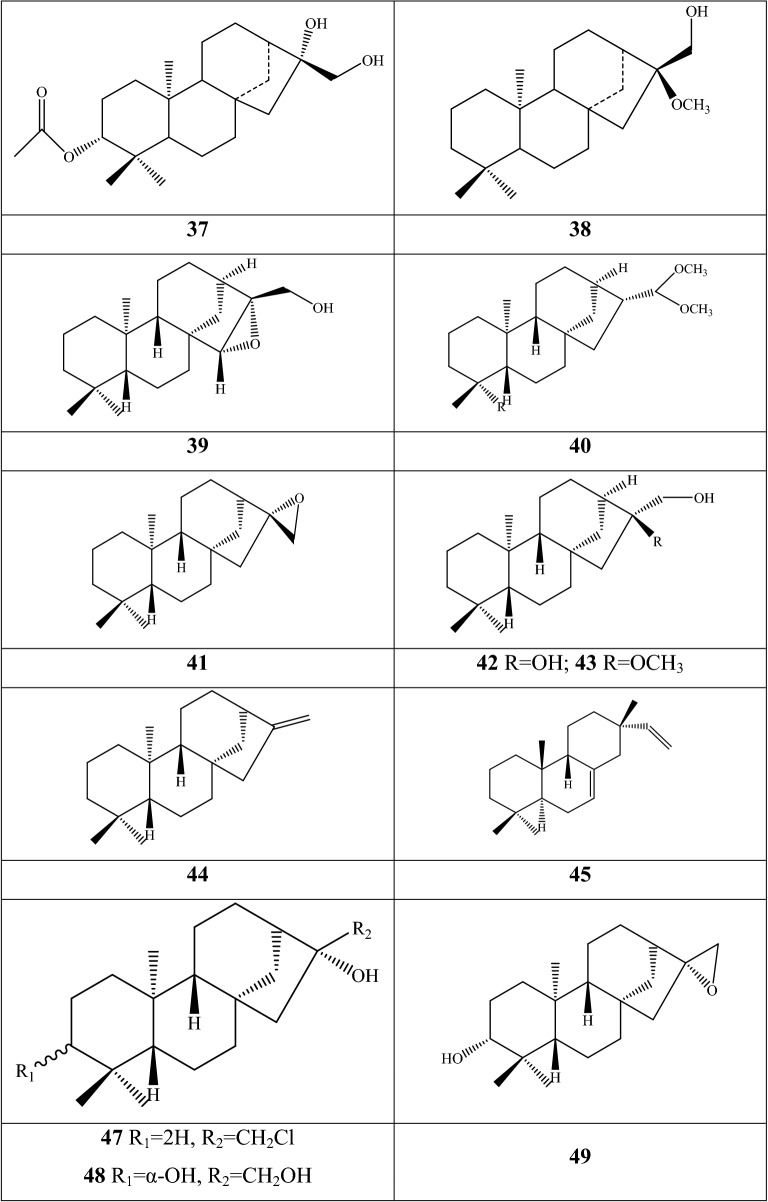
Structures of terpenoid in *Fritillaria* species

40,000 terpenoids compounds have been authenticated and characterized as the most abundant plant-specialized metabolites in the plant [52]. 49 terpenoids have been discovered from seven *Fritillaria* species. The occurrence of the types of metabolites in each species and botanical part are showed in Additional file 1: Table S4 and Fig. 4. Two new cycloartane triterpenoids (**1** and **2**) were isolated from the leaves and stems of *F. hupehensis* by chemical and spectroscopic techniques; compound **2** was the firstly found in the genus [53]. Another two similar triterpenoids were further discovered from the same botanical parts of the species, and their structure were elucidated as 25-hydroxyl-9,19-cycloart-22-ene-3-one **18** and cycloeucalenol **19** [54]. The bulbs contain other terpenoids constituents, several of which are dominantly characterized as diterpene type. Tetracyclic diterpenoids account for high percentage, with 22 components (**11**–**14**, **17**, **20**, **22**, **25**–**28**, **36**–**39**, **40**–**44**, and **46**–**49**) obtained from six species, encompassing *F. thunbergii*, *F. ebeiensis*, *F. ebeiensis* var*. purpurea*, *F. anhuiensis*, *F. hupehensis*, and *F. monanth*, which are native in China. Component **46** is a novel *ent*-kauran diterpenoid with chloro-substituted structure established as ent-kauran-16β-hydroxy-chloride from *F. ebeiensis* var*. purpurea* by spectral methods*. F. ebeiensis* was discovered the presence of nine diterpenoid dimers: fritillebin A **15**, fritillebin B **16**, fritillebin C **3**, fritillebin D **4**, fritillebinide A **5**, fritillebinide B **6**, fritillebinide C **7**, fritillebinide D **23**, and fritillebinide E **24**. Herein, component **6** was also described in a variety of *F. ebeiensis*, that is, *F. ebeiensis* var. *purpurea*, which is mainly distributed in the northwest region of Hubei Province, China. Two tricyclic diterpenoids (**8** and **9**) were detected in the ethanolic extract of *F. imperialis*, whereas one (**45**) was identified from the methanol extract of *F. thunbergii*; their structures were elucidated as isopimara-7,15-dien-19-oic acid **8**, isopimara-7,15-dien-19-methyl ester **9**, and isopimara-7,15-dien **45**, respectively. Nine bicyclic diterpenoids (**10**, **21**, **29**, **30**–**32**, **33**–**35**) have been reported in two Chinese species, namely, *F. anhuiensis*, and *F. ebeiensis*.

### Steroidal saponins

**Fig. 5:**
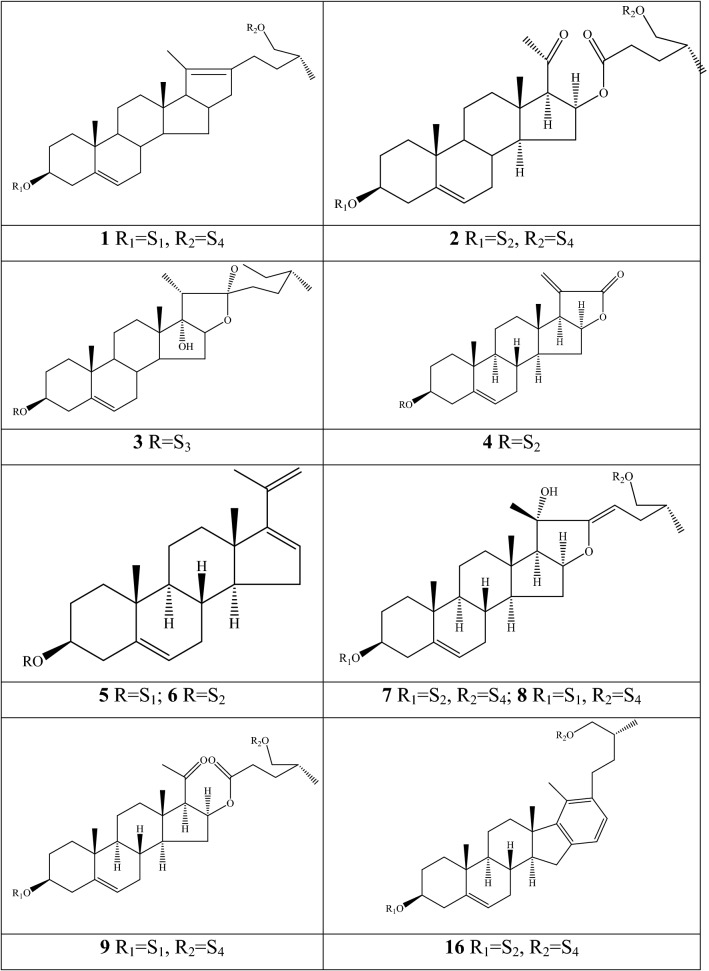

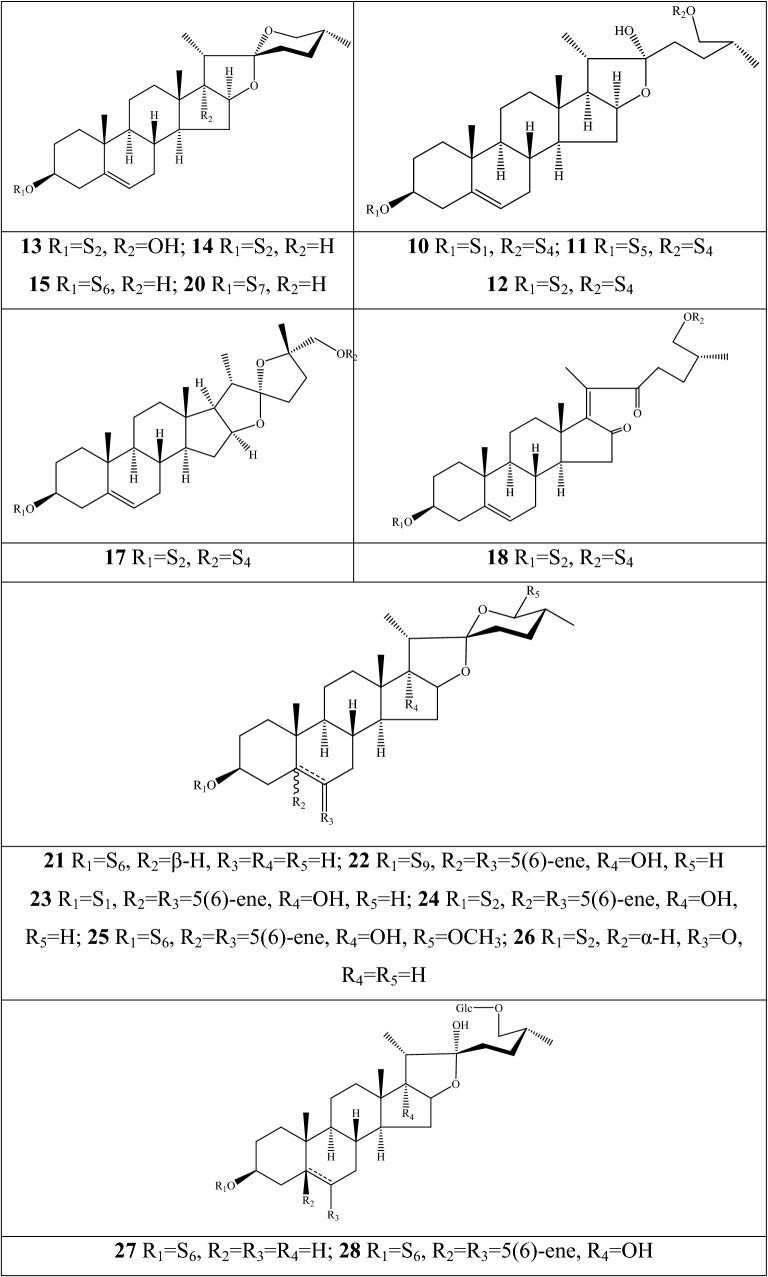

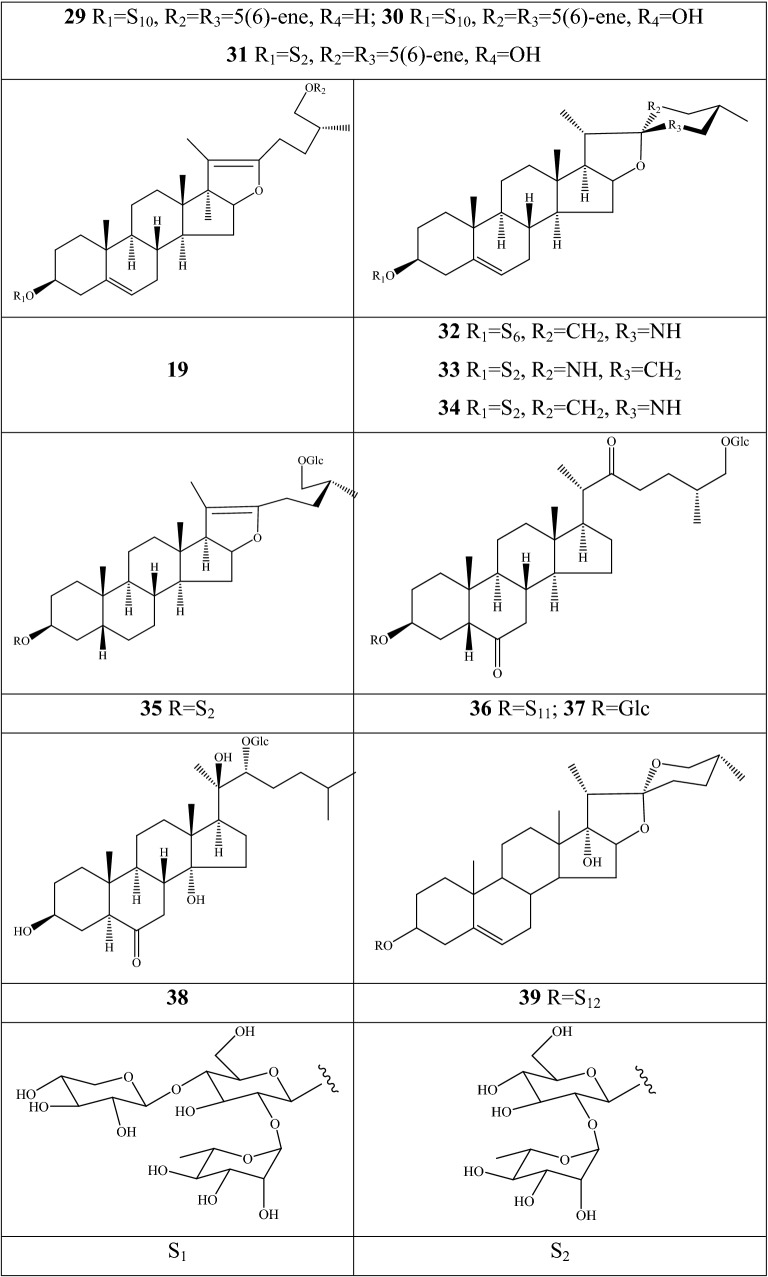

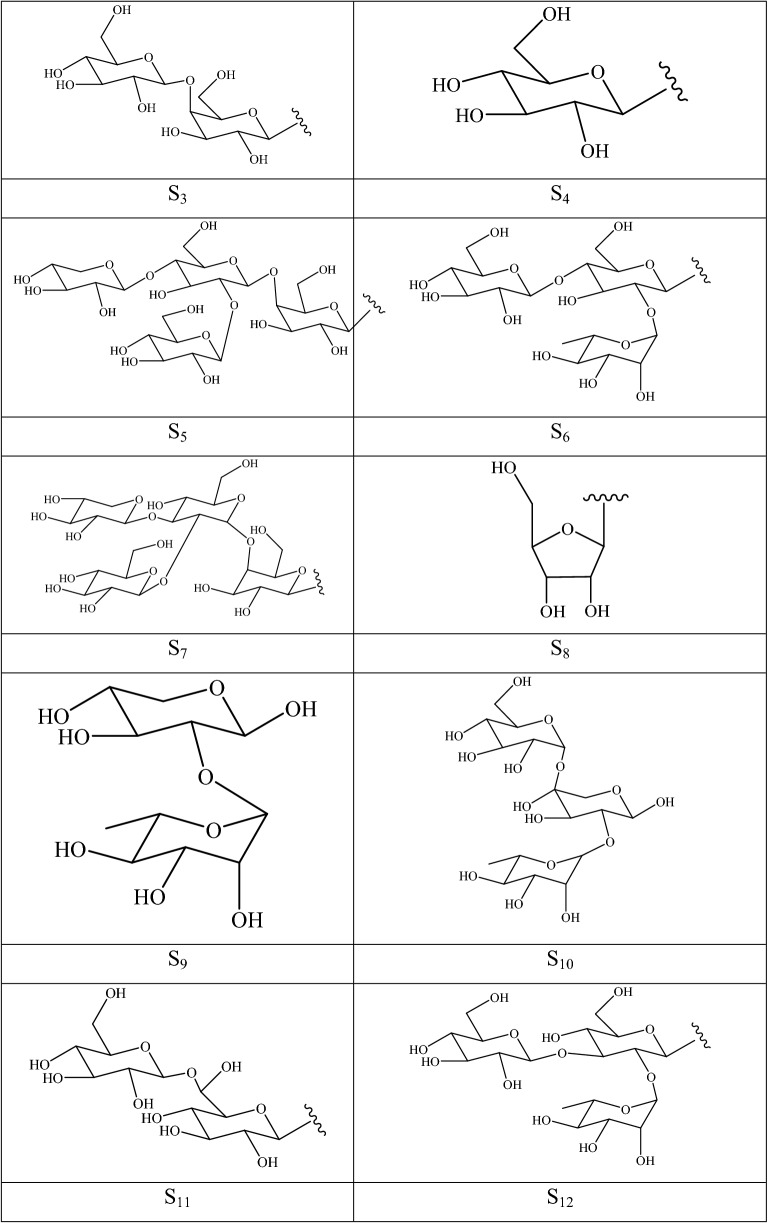
Structures of steroidal saponins in *Fritillaria* species

An impressive diversity of steroidal saponins have been discovered in the dried bulbs of *F. pallidiflora* and *F. meleagris*; no study reported obtaining these components from aerial parts (Additional file 1: Table S5 and Fig. 5). Herein, *F. pallidiflora* is a genuine-geographical Bei Mu species in Xinjiang Province of China, whereas *F. meleagris* extensively grows in Asian and northwestern Europe [55]. The early isolation and structural elucidation of steroidal saponins was dated from 2011, when three new constituents were identified as 26-*O*-β-d-glucopyranosyl-(25R)-furost-5,20(22)-dien-3β,26-diol-3-*O*-β-d-xylopyranosyl(1→4)-[α-l-rhamnopyranosyl(1→2)]-β-d-glucopyranoside, (25R)-spirost-5-ene-3β,17α-diol-3-*O*-β-d-glucopyranosyl(1→4)-β-d-galactopyranoside, and 26-*O*-β-d-glucopyranosyl-3β,26-dihydroxyl-20,22-seco-25(R)-furost-5-en-20,22-dione-3-*O*-α-l-rhamnopyranosyl(1→2)-β-d-glucopyranoside from *F. pallidiflora*, which were named as pallidifloside A **1**, pallidifloside B **2**, and pallidifloside C **3**, respectively [56]. Thereafter, pallidifloside D **4**, pallidifloside E **5**, pallidifloside G **8**, pallidifloside H **9**, and pallidifloside I **10** were obtained from the same species by the same research team [57]. 7 components (**6**, and **11–16**) were first reported in *Fritillaria*; especially the polyphyllin V and parispseudoside B, the main chemical components in *Paris*, and spongipregnoloside A was detected in nearly 10 *Paris* species [58]. The same constituents of *Fritillaria* and *Paris* can be interpreted as important clades among the 60 genera of Liliaceae. (25R)-∆^5(6)^-Isospirost-17α,3β-diol-3-*O*-β-d-glucopyranosyl-(1→3)-[α-l-rhamnopyranosyl-(1→2)]-β-d-glucopyranoside **39** is a novel compound that was first authenticated in *F. pallidiflora* [59], whereas constituents **17**–**20** were reported in one of the original plants of Yi Bei Mu. Matsuo and her co-authors [55] conducted a comprehensive investigation in terms of the extraction techniques of steroidal glycosides and their cytotoxic activities in *F. meleagris*. A total of eighteen steroidal saponins (**21**–**38**), with 10 compounds authenticated as new structures, were extracted from hot by methyl alcohol.

### Phenylpropanoids

**Fig. 6:**
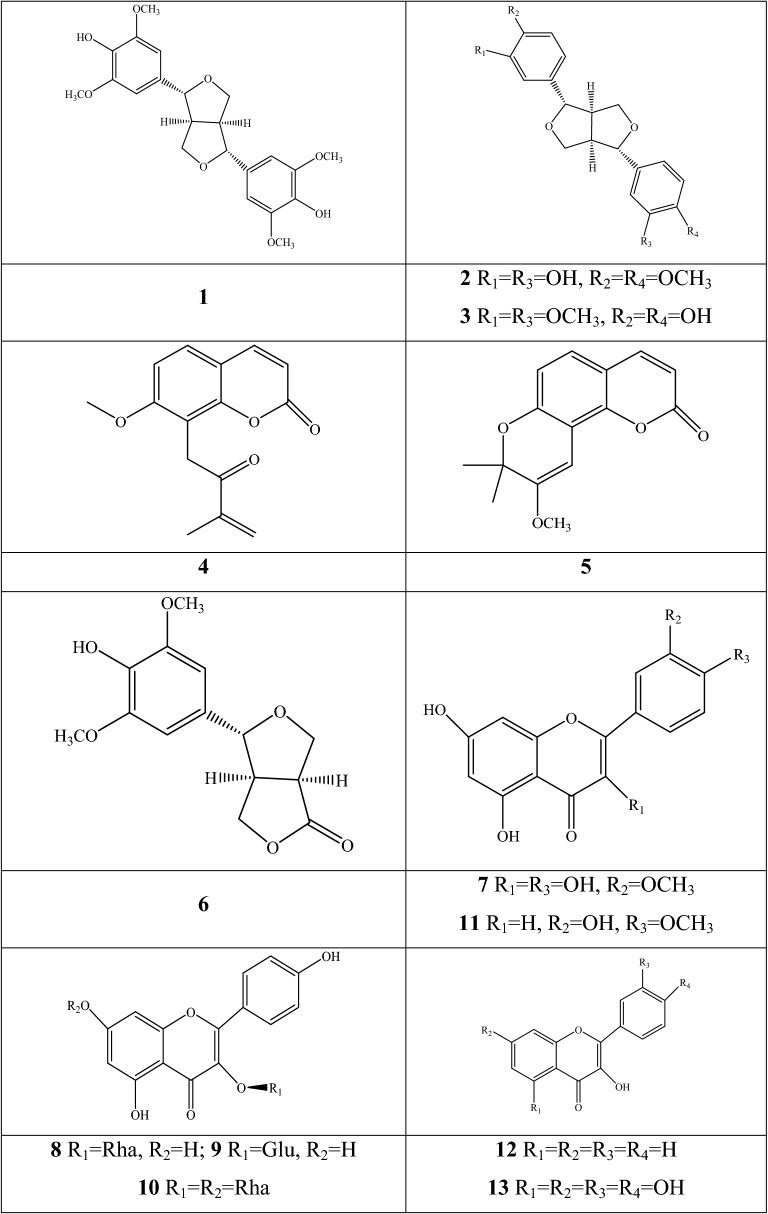
Structures of phenylpropanoids in *Fritillaria* species

The term phenylpropanoid describes a broad conception of herbal medicine possessing one or several C6–C3 skeletons; this group includes simple phenylpropanoids, lignans, coumarins, lignins, and flavonoids. Amongst, lignans, coumarins, and flavonoids have been isolated in *F. pallidiflora* and *F. thunbergii*, which were reported to yield 13 phenylpropanoid constituents from their hypogeal and aerial parts (Additional file 1: Table S6 and Fig. 6). Three bisepoxylignans were detected in the bulbs (syringaresinol **1** and pinoresinol **3** in *F. pallidiflora*), aerial parts (syringaresinol **1** in *F. thunbergii*), and flowers (clemaphenol A **2** in *F. pallidiflora*), and they were characterized by two tetrahydrofuran rings formed by two phenylpropanoid molecules. A single bisepoxylignan, that is, zhebeiresinol **6**, was described in the aerial parts of *F. thunbergii*. Two coumarins (murrayone **4** and 2′-methoxyseselin **5**) were extracted and autenticated from the bulbs of *F. pallidiflora*. Seven flavonol compounds (**7**–**13**) belonging to flavonoids exist in the bulbs and flowers of *F. pallidiflora* and flowers of *F. thunbergii*, in which flavonol **8**–**10** are glycosides connected with rhamnose or glucose at the C3 position.

### Fatty acids

Five species were isolated and authenticated, and six fatty acids were reported to originate from the bulbs of *Fritillaria* (Additional file 1: Table S7). Stearic acid was isolated from *F. michailovskyi* for the first time [22]. Shi and her co-authors isolated for the first time linoleic acid from *F. walujewii*, which also was the earliest isolation conducted on the genus [60]. Lignoceric acid and azelaic acid discovered in the bulbs of *F. hupehensis*, without records in other species of the genus until now [61]. Palmitic acid was detected in two species (*F. hupehensis* and *F. michailovskyi*) [22, 61], whereas laurostearic acid was reported in *F. pallidiflora* [59].

### Sterides

Nine species of the genus have been reported that their bulbs or aerial yielded β-sitosterol, which is widely distributed in the plant domain and an important components of plant cells (Additional file 1: Table S8). This plant-specialized component has been reported in the bulbs/aerials parts of *F. thunbergii* [62, 63], flowers of *F. pallidiflora* [64], and bulbs of *F. anhuiensis* [29], *F. unibracteata* [51], *F. michailovskyi* [22], *F. hupehensis* [65], *F. thunbergii* var. *chekiangensis* [66], *F. monantha* [67], and *F. walujewii* [60]. The C3-OH is connected to a glucose-forming daucostenine (β-sitosterol-glucoside), which was identified from bulbs of *F. anhuiensis* [29] and *F. michailovskyi* [22].

### Other metabolites

Apart from the six types of metabolites mentioned above, several other compounds have been identified in *Fritillaria* taxa, in which chemical components (**1**–**59**) were encompassed in four botanical parts of 11 species (Additional file 1: Table S9). Several common constituents related to the plant physiology have been isolated and identified since 2012, when a cyclic peptide was extracted from the bulbs of *F. anhuiensis* and whose structure was elucidated as cyclo-(Leu–Val) based on spectroscopic analysis [29]. The presence of this component was reported in Liliaceae for the first time. Recently, l-pyroglutamic acid, Cyclo (l-Pro-l-Ala) and Cyclo-(Phe-Val), as two new cyclic peptides, were obtained from the medicinal bulbs of *F. pallidiflora* in 2016 [64] and 2019 [68], respectively. Choline was first discovered in the bulbs of *F. walujewii*, which was also the earliest discovery in the genus [60]. Several nitrogenous bases, their glycosides, and nucleoside compounds were also discovered in certain species of *Fritillaria*, such as uridine, uracil, adenosine, thymidine, thymine, adenine, and guanosine, which take part in genetic evolution and other physiological activities. Followed by small-molecule alcohols (**13**–**22**), components **15**–**17** were separated by water steam distillation from volatile oil and authenticated by gas chromatography–mass spectrometry in the bulbs of *F. cirrhosa* [69]. Some esters (**23**–**37**) were also obtained from the bulbs or flowers. Certain acid components, such as cinnamic acid (**38** and **40**) [59, 68] and 4-(β-d-glucopyranosyloxy) benzoic acid [70], rather than fatty acid were extracted in certain *Fritillaria* species, such as the bulbs of *F. pallidiflora.* Ketones occur with four compounds and, comprised of **42**–**45**, were present in the flowers of *F. thunbergii* [71] and the bulbs of *F. cirrhosa* [69] and *F. pallidiflora* [68]. Hydrocarbons identified as 1-dodecene **46**, 4-octadecene **47**, and pentatriacontane **48** were detected in *F. cirrhosa* [69] and *F. michailovskyi* [22]. Other chemical components are displayed in Additional file 1: Table S9, in which gastrodin **49** is the index component for the quality control of *Gastrodia elata* Bl, whereas icariside D2 **50** (4-*O*-β-d-glucoside of tyrosol) is an effective constituent that was first isolated from *Epimedium diphyllum* [72]. An acidic water-soluble heteropolysaccharide, which is a macromolecule with good antioxidant activity and DNA protection effect, was extracted and purified from *F. unibracteata* var*. wabuensis*, which implies that the small-molecule compound is not the only active component in *Fritillaria* species [73]. In addition, the herbal medicine made from *Fritillaria* is rich in chemical components that were not characterized nor investigated in the study.

Alkaloids (isosteroidal, steroidal, and amide alkaloids) are certainly the main chemical constituents of *Fritillaria*. The portions of terpenoids and steroidal saponins account for a high percentage of these constituents and should be given attention in-deep investigation of the genus. Although vast literatures focused on the isolation and authentication of chemical components, limited studies reported the systematic protocol of individual compounds with the help of molecular or genetic methods. Sun et al. found those alkaloid biosynthesis genes in *F. cirrhosa* with the help of two common database, Gene Ontology and Kyoto Encyclopedia of Genes and Genomes databases [74]. In addition, de novo transcriptomics is regarded as a useful approach in interpreting genes of complex biochemical pathways to identify steroid alkaloid biosynthesis in *F. imperialis* [75]. In the future, researchers may pay attention to the investigation and validation of these biosynthesis genes of the main chemical components of *Fritillaria* species with the aid of genomics, proteomics, biochemical, and computational analyses. The biochemical pathways will decrease the production cost because of the long cultivation period of *Fritillaria* species. What’s more, the extraction of chemical components from the aerial parts would provide reference for the resource utilization of these species of the genus, because the aerial parts have been discarded after the bulbs were harvested.

## Pharmacological activities

The dried bulbs of *Fritillaria* species have been used as an anti-tussive drug and other respiratory diseases, such as expectoration and asthma, in traditional folk medicine since Han Dynasty of China (around AD 220). Thus, several studies focused on the respiratory diseases in vivo and in vitro. Aside from studies on the respiratory system, the increasing pharmacological research indicates that the chemical components or extracts from herbal medicines have potential antineoplastic, anti-inflammatory, antihypertensive, bacteriostasis, and anti-tumor effects (Additional file 1: Table S10).

### Respiratory diseases

**Fig. 7:**
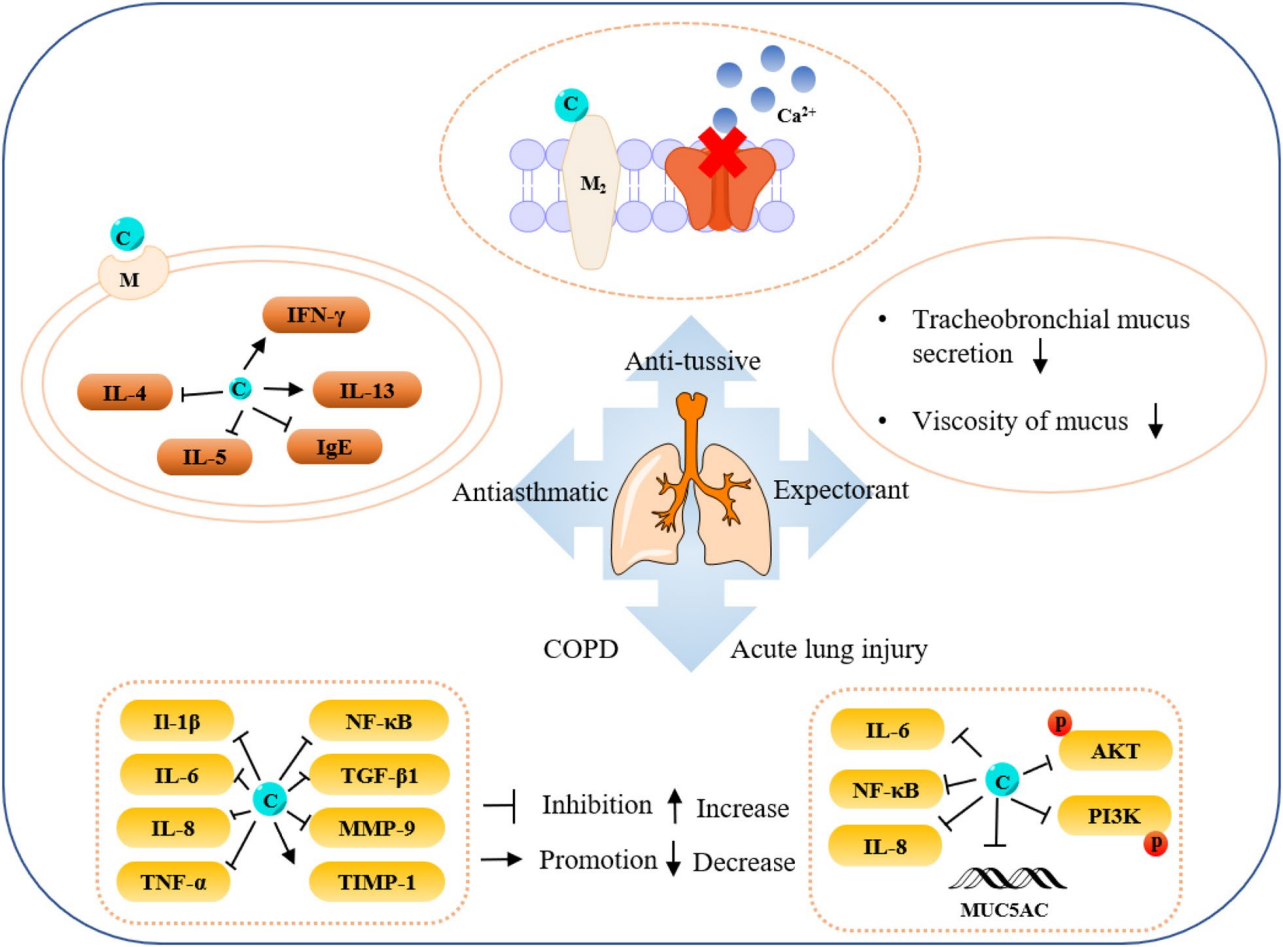
The action mechanism of respiratory diseases using chemical components from *Fritillaria* species

Respiratory diseases seriously affect the physical and mental well-being of patients with symptoms of sneezing, cough, and difficulty in breathing, which are leading causes of mortality and morbidity [76]. The natural botanical materials from *Fritillaria* have significant pharmacological effects on the respiratory system, including the alleviation of cough, phlegm, asthma, COPD, and acute lung injury (ALI) (Fig. 7).

#### Anti-tussive effect

The anti-tussive efficacy is consistent pharmacological activity between the traditional clinical usage and modern utilization in the daily life. Anti-tussive pharmacological comparison of 11 commercial *Fritillaria* species indicated that the total alkaloid of 11 *Fritillaria* species had significant or extremely significant effect on ammonia-induced cough in mice. The ethanol extracts of 9 *Fritillaria* species, except for *F. delavayi* and *F. pallidiflora*, also have significant anti-tussive effect [77]. Further studies confirmed that steroidal alkaloids are the main effective components in these species, and they play vital roles in anti-tussive activity. Individual investigation of four new steroidal alkaloids (**33**, **34**, **62**, and **63**) isolated from the bulbs of *F. puqiensis*, was performed their anti-tussive activity on mouse induced with ammonia liquor. The results indicated that four compounds reduced the coughing times and prolonged the latent period [43]. Other study obtained consistent performance with the induction studies of ammonia liquor [78–81]. Ammonia-induced cough experiment showed that four steroidal alkaloids (**2**, **8**, **11**, and **13**) from *F. cirrhosa* and three components (**4**, **20**, and **21**) from *F. wabuensis*, significantly inhibited cough frequency and increased the latent period of in mice [78, 79].

Ebeinone **26** of *F. imperialis* strongly exhibited a higher affinity for muscarinic M_2_ receptors than for M_3_ receptors in a study on guinea pigs in 1997, and it interacted allosterically with M_2_ receptors during a rat trail [82]. The same results were described for five alkaloids (**2**, **11**, **13**, **88**, and **61**), demonstrating significant elevation of the cAMP concentration in the human embryonic kidney cells transfected with muscarinic M_2_ receptor plasmid [83]. The action mechanism of imperialine **11** and sinpeinine A **6** was interpreted as their selective inhibitory on muscarinic M_2_ receptors, whereas the mechanism of 3β-acetyl-imperialine was performed by its selective antagonism of muscarinic M_3_ receptor [84]. Chan and co-authors compared the relaxant effects of five major steroidal alkaloids (**2**, **11**, **13**, **99**, and **61**); the former four components have a cevanine-type structure, whereas puqietinone **61** is a verazine alkaloid, as investigated by rat-isolated tracheal and bronchial preparations pre-contracted with carbachol. The results demonstrated the potential mechanisms of these constituents were competitive antagonism of muscarinic pathway and the inhibition of Ca^2+^ influx [85].

#### Expectorant effect

The expectorant effect can be observed in the mixed preparations of fresh pear and dried powders, which are normally cooked by the elderly for their therapeutic effects. The total alkaloids and saponins are the prominent compounds contributing to the expectorant effect. Compounds **2**, **11**, and **13** from *F. cirrhosa* and **4**, **11**, **20**, and **21** from *F. wabuensis* enhanced the phenol red output from a mouse tracheal [78, 79]. Expectorant effects are generally related to the relaxation of smooth muscles. The sputum volume was increased after the drug was fed in rats without a vagus nerve, thus which confirming that the bulbs of *Fritillaria* are a non–evil expectorant [86].

#### Anti-asthmatic effect

Asthma is an allergic disease caused by broad bronchial obstruction and exhalation dyspnea is the main symptom. The main inducements of bronchial obstruction are interpreted by three aspects, including bronchial smooth muscle contraction, excessive mucus secretion, and adhesion to the bronchial wall. The main anti-asthmatic mechanisms comprise the relaxation of bronchial smooth muscles, relief of trachea and bronchus spasm, and improvement of ventilation status. Current scientific research shows that the anti-asthmatic effect of *Fritillaria* is related to the antagonism of the tracheal M receptor [82, 83]. The water extract of *F. cirrhosa* exhibits obvious inhibitory effects on airway inflammation by several pathways, which includes the suppression of helper T cell-2 cytokines and immunoglobulin-E, histamine production, reduction of eosinophilic accumulation, and increase in interferon-γ (IFN-γ) production [87]. Yibeinones B–D (**80**–**82**) and imperialine (**11**) isolated from the bulbs of *F. pallidiflora* showed an evident concentration-dependent relaxation effect on the isolated tracheal preparation, whereas components **11** and **81** exhibited significant effects with pA2 values of 6.19 ± 0.02 and 8.41 ± 0.10, respectively [41].

#### Other respiratory diseases

The effects of bulbs from *Fritillaria* species have been extended to other respiratory diseases, such as COPD and ALI. Wang et al. investigated the performance of imperialine **11** on pulmonary function and inflammation in a COPD-like rat model, of which the model was established by the exposure to cigarette smoke and intratracheal administration using lipopolysaccharide (LPS). The obtained results displayed that the alkaloid mitigated pulmonary impairment and suppressed the inflammatory response by mediating the expression of related cytokines in lung tissues [88]. The therapeutic effect of peiminine **13** implied that the component can reduce the wet-to-dry ratio and the myeloperoxidase activity on LPS-induced ALI. IL-6 was inhibited after peiminine treatment. Peiminine also showed a significant inhibition trait for the LPS-induced IL-8 production in human lung adenocarcinoma cells (A549). Meanwhile, Western blot analysis displayed that peiminine remarkably suppressed the activity of the NF-κB pathway. In addition, peiminine disrupted lipid raft formation by attenuating the cholesterol content [89]. The extracts of *F. thunbergii* have been used as mucoregulators for controlling airway inflammatory diseases, which is related to the expression of mucin 5 subtype AC (MUC5AC). Three isosteroidal alkaloids (**2**, **72**, and **99**) inhibit the gene expression of MUC5AC by directly acting on airway epithelial cells [90].

### Antineoplastic effect

**Fig. 8:**
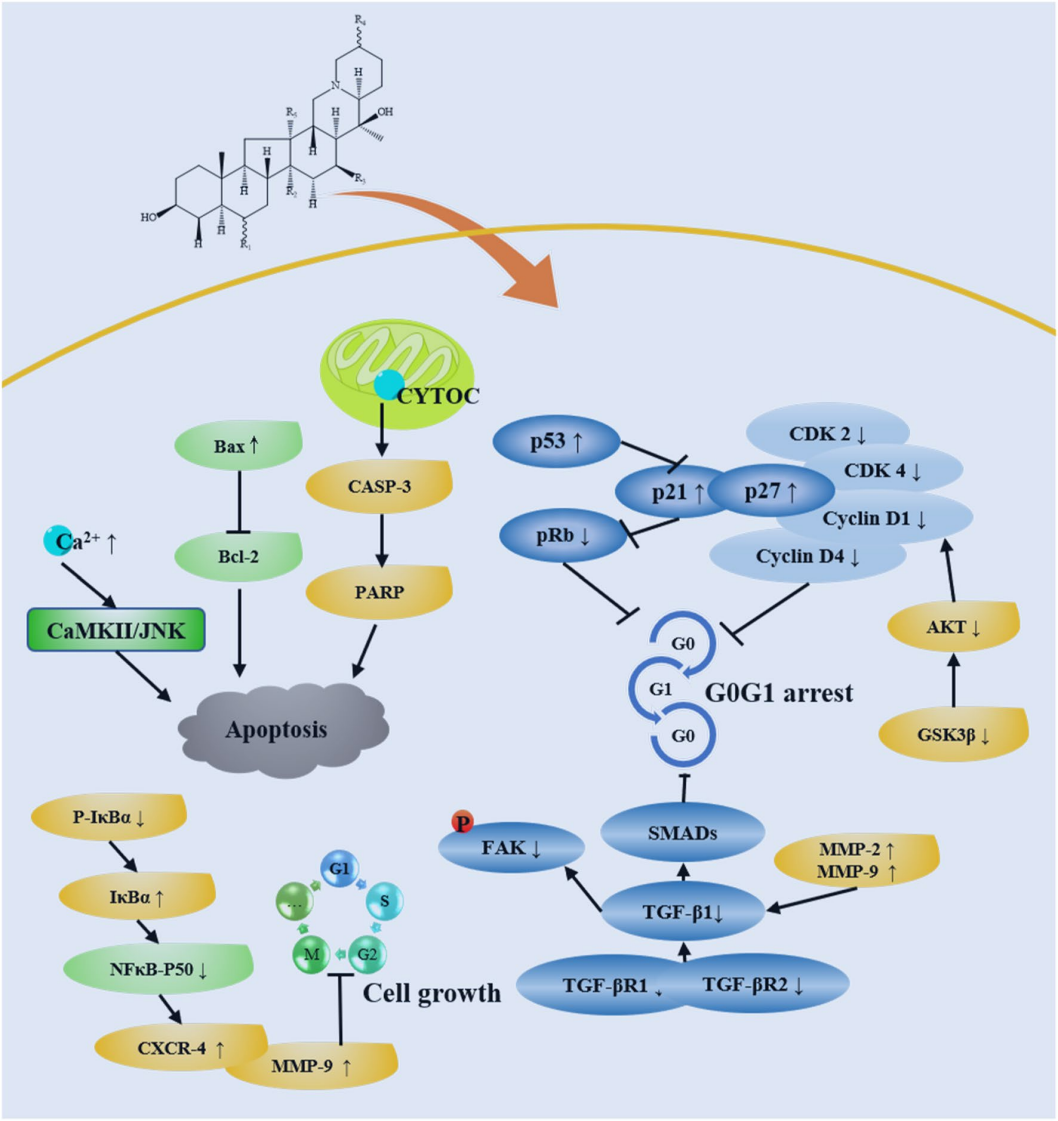
Antineoplastic effect of chemical components and extract in *Fritillaria* species

Plant-derived agents may became a potential resource with a vital role in antineoplastic treatment; the therapeutic properties of several species have been discovered in a variety of cancers or cancer cells, such as human promyelocytic leukemia cells (HL-60) [55, 91], A549 cells [55], oral keratinocytes [92], ovarian and endometrial cancer cell lines [93, 94], *S180* sarcoma and Lewis lung carcinoma [88, 95], HeLa cells [96], human colorectal carcinoma cells [97], glioblastoma (GBM) cells [98], non-small cell lung cancer (NSCLC) [99], and prostate cancer [100] (Fig. 8). These modern pharmacological results were consistent with the ancient efficacy showing that Bei Mu herbs can remove stasis and tumor. The antineoplastic effect is mainly performed by the proliferation inhibition of cancer cells. In recent years, several studies have been conducted to explore the potential mechanisms of anticancer activity using chemical components or extracts from *Fritillaria* herbs; These mechanisms include promotion of cell differentiation [91], induction of apoptosis and G_0_G_1_ cell cycle arrest [95] through a caspase pathway [88, 92], apoptosis induction without affecting the caspase-3 activity level [55], activation inhibition of NF-κB [93, 99], downregulation of TGF-β/SMAD signaling pathways [94], inhibition of tumor angiogenesis [88], induction of cellular stress and activating several anti- and pro-survival pathways [96], induction of autophagic cell death via activating autophagy-related signaling pathway AMPK-mTOR-ULK by promoting SQSTM1 (P62) [97, 98], and disruption of intracellular calcium homeostasis through Ca^2+^/calmodulin-dependent protein kinase II (CaMKII) and c-Jun N-terminal kinase (JNK) pathway [100]. Peimine, as a common component of 12 *Fritillaria* species, has showed reversal of the multidrug resistance of tumor cells in vitro and reversed tumor cells through in vitro multidrug resistance activity. Peimine was the first steroidal alkaloid reversing drug resistance of tumor cells, and its mechanism may be interpreted that of increasing concentration in drug-resistant cells and the expression inhibition of P-glycoprotein protein in drug-resistant cells [101].

The water extract of *F. cirrhosa* can significantly decreased cell growth and the invasive potential of ovarian and endometrial cancer cell lines. The mechanism was interpreted to be caused by the in activation of caspase-3, G_0_/G_1_ phase cell cycle arrest, downregulation of cyclins D1 and D3, induction of p27, decreased NF-κB DNA binding, expression reduction of phosphorylated IkBa, abrogated NF-κB activation, and downregulation of NF-κB-regulated metastasis-promoting proteins [93]. Investigation of the antitumor activity of different extraction fractions and isosteroidal alkaloids from *F. ussuriensis* showed that the chloroform extract and purified alkaloids exhibited stronger cytotoxic activity. Four main steroidal alkaloids (**2**, **11**, **44**, and **13**) were isolated and showed significant cytotoxicity; peimisine **44** can induce G_0_/G_1_ phase arrest and promote apoptosis [94]. Four cevanine-type steroidal alkaloids (**4**, **8**, **20**, and **21**) isolated from the total alkaloids of *F. pallidiflora* displayed significant cytotoxicity, with chuanbeinone **8** showing the highest activity against Lewis lung carcinoma cells. The results displayed that the antitumor activity was exhibited the compound in vivo, in which the increased expression of caspase-3 generated the performance of tumor angiogenesis and induced apoptosis [88].

Verticinone **13**, which exists in approximately 13 *Fritillaria* species, inhibited the growth of HL-60 cells by inducing their differentiate into granulocytes [91] and existed apoptosis induction via a caspase pathway [92]. Component **21**, (25R)-5β-Spirostan-3β-ylO-β-d-glucopyranosyl-(1→4)-*O*-[α-l-rhamnopyranosyl-(1→2)]-β-d-glucopyranoside, was authenticated as a steroidal saponin from the dried bulbs of *F. meleagris*; it induces the apoptotic cell death of HL-60 cells through several mechanisms. Component **32**, (22S,25S)-Spirosol-5-en-3β-ylO-β-d-glucopyranosyl-(1→4)-*O*-[α-l-rhamnopyranosyl-(1→2)]-β-d-glucopyranoside, can selectively induced apoptosis in A549 cells which can’t affect the activity level of caspase-3 [55]. Isopimara-7,15-Dien-19-oic acid, a terpenoid isolated and authenticated from the dried bulbs of *F. imperialis*; it induced cellular stress and activated several anti- and pro-survival pathways in HeLa cells [96]. Peiminine suggest a new strategy for GBM therapy, which induction of autophagic cell death is performed by activating the autophagy-related signaling pathway AMPK-mTOR-ULK and promoting SQSTM1 (P62) [97] as well as inhibiting GMB in vitro and in vivo via arresting the cell cycle and blocking autophagic flux [98]. Imperialine exerted anti-cancer effects against NSCLC, which potential mechanism was related to the NF-κB centered inflammation-cancer feedback loop [99]. In addition, the toxicity assays revealed that imperialine treatments did not significantly disturb the blood cell counts in mice nor exert any significant damage to the main organs, indicating a robust systemic safety [99]. Peimine can generated inhibition performance of the growth and motility of prostate cancer cells and is interpreted as the disruption of intracellular calcium homeostasis through the Ca^2+^/CaMKII/JNK pathway [100].

### Anti-inflammatory effect

**Fig. 9:**
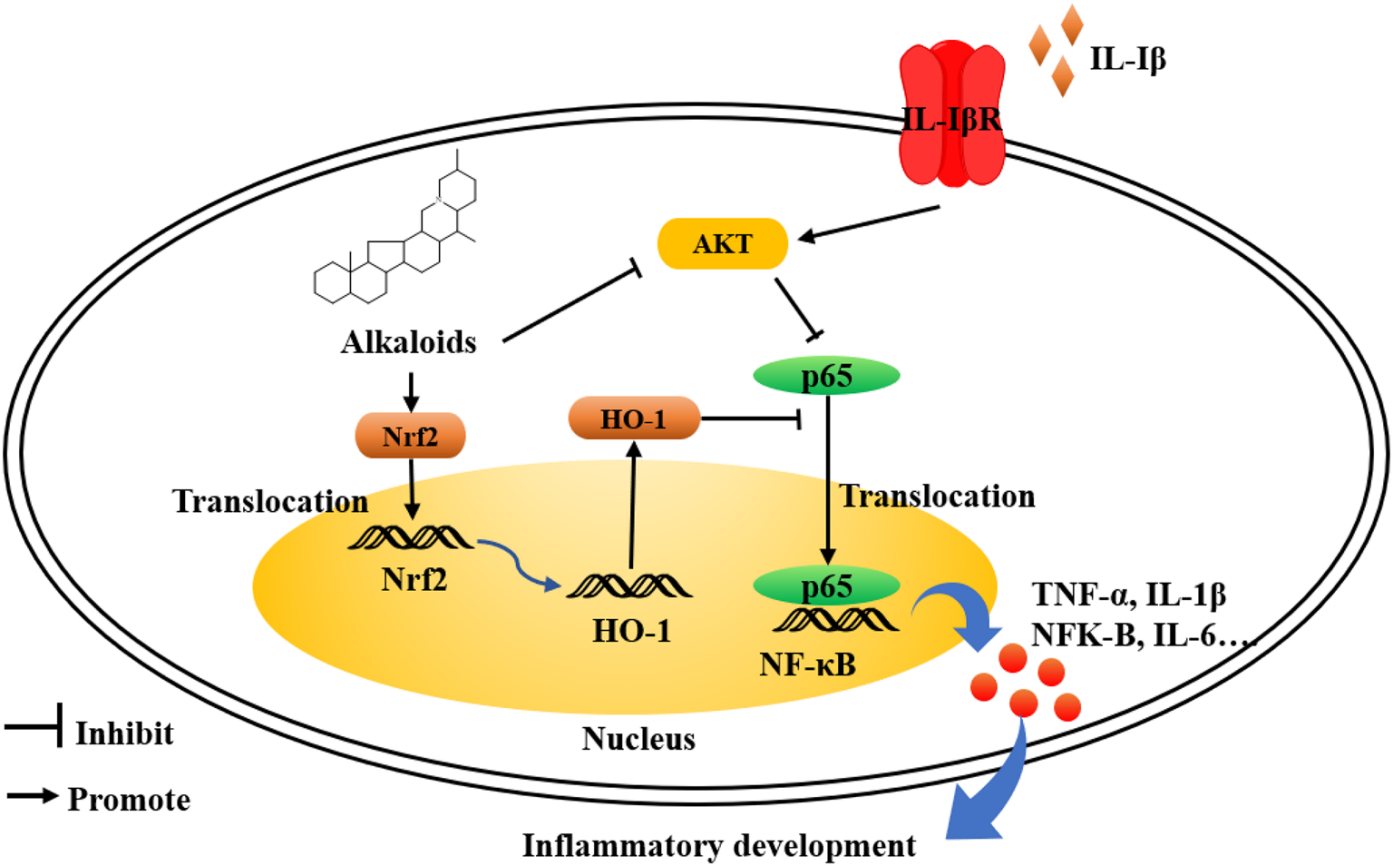
Anti-inflammatory effect of chemical components in *Fritillaria* species

The bulbs of *Fritillaria* have a certain therapeutic effect on COPD and ALI as mentioned above, and this phenomenon is closely related to their anti-inflammatory effect (Fig. 9). The ethanol extracts of two commercial species (*F. cirrhosa* and *F. pallidiflora*) inhibited the development of ear edema [81]. Chuanbeinone **8** and imperialine **11** have the same inhibition performance in ear edema of an anti-inflammatory assessment depending on a dose-dependent manner [78]. Four isosteroidal alkaloids (**4**, **11**, **20**, and **21**) obtained the same anti-inflammatory performance [79]. The alkaloid fraction of *F. cirrhosa* showed several anti-inflammatory activities (inhibition of acetic acid-induced capillary permeability accentuation, carrageenan-induced paw edema, and cotton pellet-induced granuloma formation; suppressing recruitment of inflammatory cell and production of cytokine in the bronchoalveolar lavage fluid from ALI mice) [102]. 12,15-Sulfonyl-8(17),13-labdadien-19-oic acid (terpenoid component **21**) isolated from *F. anhuiensis* obviously attenuated the NO production in a macrophage cell line of RAW264.7 cells stimulated with IFN-γ [103].

In addition, the anti-inflammatory mechanism of *Fritillaria* bulbs is interpreted as closely related to various signal pathways (the inhibition of NF-κB [104, 105], expression reduction of inflammatory cytokines [102, 104, 106, 107], inhibition of the mitogen-activated protein kinase (MAPK) pathway [108, 109].) Two main components (verticinone **13** and imperialine **11**) can suppress the production of pro-inflammatory cytokines with a dose-dependent manner. The crude polysaccharide, extracted from *F. hupehensis* by hot water, also displayed obviously inhibition performance to mouse ear swelling induced by xylene and the toe swelling induced by egg white, in which the inhibition performance was interpreted that the secretion of inflammatory cytokines, meanwhile dynamic balance between pro-inflammatory and anti-inflammatory factors [106]. Marker component (peimine **2**) suppressed IL-1β-induced inflammation in mouse chondrocytes and inhibited he LPS-induced RAW264.7 macrophages via inhibition of the MAPK pathway [108, 109]. What’s more, the chemical component can ameliorate murine osteoarthritis via inhibition of the AKT phosphorylation, nuclear transfer of NF-κB and activation of nuclear factor 2 (Nrf2)/heme oxygenase-1 in an osteoarthritis model [107].

### Antihypertensive effect

Antihypertensive effect is a novel pharmacological activity that is different from the traditional ethnopharmacological properties of *Fritillaria* species. The water extract of *F. ussuriensis* can prevent the increase of systolic blood pressure in N^G^-nitro-l-arginine methylester-induced hypertension and interpreted as the enhanced generation of vascular NO and amelioration of renal functions [110]. The comparison of different extracts of *F. ussuriensis* showed that intravenous injection of water extract can decrease the mean arterial pressure of anesthetized rats. The angiotensin converting enzyme (ACE) activities were significantly inhibited by ethylacetate and butanol extracts (half maximal inhibitory concentrations were 292 and 320 mg ml^−1^, respectively). The results showed that the extracts had a hypotensive effect via the ACE inhibition and release of NO/cyclic guanosine 3ʹ,5ʹ-monophosphate in the vascular tissue on rats [111]. Alkaloids components **2**, **13**, and **44**, which were isolated from the dried bulbs of *F. ussuriensis*, have ACE inhibition activity in a dose-dependent manner (50% inhibitory concentration values of 165.0, 312.8, and 526.5 μM, respectively) [112]. Three veratramine-type isosteroidal alkaloids (**34**, **35**, and **39**) isolated from *F. puqiensis* exhibited an inhibitory activity (inhibition ratios of 70.2% ± 0.5%, 24.7% ± 0.5% and 20.4% ± 2.8%, respectively, at the concentration of 200 µM) [42].

### Anti-cholinesterase (cholinomimetic) activity

Acetylcholinesterase (AchE) inhibition is considered a promising strategy for the treatment of Alzheimer’s disease [113]. Herein, the dichloromethane fraction of *F. michailovskyi* shows a positive butyrylcholinesterase (BchE) inhibitory activity [22]. Zhou compared the antimuscarinic effects of five *Fritillaria* species (*F. puqiensis*, *F. hupehensis*, *F. ebeiensis*, *F. pallidiflora*, and *F. delavayi*). The results showed that the methanol extracts of *F. unibracteata* generated the best antimuscarinic effects with the help of several steroidal alkaloids [105]. Five steroidal alkaloids (**11**, **15**, **16**, **18**, and **22**) isolated from the bulbs of *F. imperialis*, of which forticine **15**, persicanidine A **16**, and impericine **18** were isolated from the species, showed anti-AchE and anti-BchE inhibitory activity [114]. Moreover, delavine **22** exists in *F. delavayi*, and imperialine 11 is a common constituent of the genus; *N*-demethylpuqietinone **62** is present in *F. puqiensis*, hupeheninoside **25** in *F. lichuanensis*, ebeiedinone **9** in *F. ebeiensis* or other species, and yibeinoside A **6** in *F. pallidiflora* (or other species). Chuanbeinone **8** from *F. delavayi* (or other species) has anti-red blood cell AchE and anti-plasma BchE activities [115].

### Antibacterial and antiviral activities

Pharmacological research on herbs all over the world for the selection of bioactive constituents or plant extracts against fungi and bacteria may be an alternative protocol to combat antibiotic resistance [116]. Antibacterial investigation of the ethanol and aqueous extracts of *F. thunbergii* indicated remarkably inhibitory against six *Helicobacter pylori* strains (minimum inhibitory concentrations is close to 60.0 μg ml^−1^) [117]. β-Sitosterol-3-*O*-glucopyranoside (steride component **2**) exhibits antibacterial activity against three bacterial including *Bacillus subtilis*, *Staphylococcus aureus*, and *Micrococcus luteus* [118], that the MIC values are 50, 200, and 400 μg ml^−1^, respectively. In addition, the water extract of *F. thunbergii* exerts antiviral effects against influenza H1N1 virus without inducing toxicity in vitro, in ovo, or in vivo [119].

### Antioxidant effect

The antioxidant effect occurs as species- and fraction-dependent fluctuation in *Fritillaria* species. The antioxidant activity of *F. ussuriensis* extracts decreases with different polarity (crude flavonoid extract > crude saponin extract > ethanol extract) [120]. Three isosteroidal alkaloids (peimisine, peimine, and peiminine) that are abundantly found in *Fritillaria* bulbs exist as acidic water-soluble heteropolysaccharides (average molecular weight: ~ 7.44 kDa) in *F. unibracteata* var*. wabuensis*; the acidic fraction FPSP-H2-1 from *F. pallidiflora* process a strong antioxidant effect against 1,1-diphenyl-2-picrylhydrazyl and 2,2ʹ-azino-bis(3-ethylbenzothiazoline-6-sulfonic acid) free radicals [73, 121, 122]. The comparison analysis of six isosteroidal alkaloids (**2**, **11**, **13**, **22**, **44**, and **88**) with different chemical structures from *F. cirrhosa* supported the results, showing that five components (**2**, **13**, **22**, **44**, and **88**) exhibited more potent effect against cigarette smoke extract-induced oxidative stress than imperialine **11** by activating the Nrf2-mediated antioxidant pathway [123].

### Antinociceptive effect

Records of traditional medicine applications show that the bulbs of the *Fritillaria* can be used for the treatment of chest heat and pain in Mongolian national minorities. Verticinone **13**, which has been extracted and authenticated from approximately 13 *Fritillaria* species, can exert a good antinociceptive performance on inflammatory and cancer-related neuropathic pains probably via peripheral and central mechanisms [124]. *Fritillaria* herbs may be used as potential pain reliever, because peimine **2** not only blocks the Nav1.7 ion channel but also inhibits the Kv1.3 ion channel [125].

### Anti-allergy effect

*F. ussuriensis* significantly inhibited the passive cutaneous anaphylaxis reaction and the release of histamine from rat peritoneal mast cells after extracted by ethanol. The activity was conducted the mechanism and interpreted as the inhibition of IL-6, IL-8, and TNF-α and the MAPKs phosphorylation [126]. The investigation of individual compounds showed peiminine **13** as the active agent [127].

### Neuroprotective effect

Kaurane diterpene and two labdane diterpenes were isolated from dried bulbs of *F. ebeiensis*, a native species in China, prevented MPP^+^-induced neuronal cell death and displaying significant neuroprotective effect on human dopaminergic neuroblastoma SH-SY5Y cells [128, 129]. Peimisine-3-*O*-β-d-glucopyranoside **66** from *F. unibracteata* and *F. yuminensis* displayed moderate protective effect on the neurotoxicity of PC12 cell lines which suffer from rotenone [39]. LPS-induced Parkinson’s disease rat model has shown that peiminine inhibited the loss of dopaminergic neurons and microglial activation and obviously attenuated the behavioral dysfunction. In BV-2 cells, peiminine **13** significantly decreased the LPS-induced expression of pro-inflammatory mediators TNF-α, IL-6 and IL-1β, cyclooxygenase-2 and inducible nitric oxide synthase with the inhibition of the phosphorylation of extracellular signal-regulated kinase 1/2, AKT and NF-κB p65 [130].

### Anti-diabetic effect

The diabetes is a major concern worldwide caused by high-sugar intake or other induction factors. The relevant investigation of verticinone **13** demonstrated the component exhibited hypoglycemic effects by increasing insulin secretion and glucose uptake. What’s more, the component can inhibit of carbohydrate-hydrolyzing enzymes on β-TC6 pancreatic and C2C12 skeletal muscle cells [131].

In general, these summarized modern pharmacological activities are consistent with the traditional records of efficacy, which showed that the dried bulbs are mainly used for respiratory diseases including their anti-tussive, expectorant, and anti-asthmatic effects. The crude materials were gradually developed to exhibit broader activities, such as antinociceptive, anti-allergy, neuroprotective, and anti-diabetic effects, with a promising utilization. However, the detailed interpretation is unclear, and modern scientific methods should be used to further study the pharmacological action to clarify the multi-target and multi-channel mechanism of Bei Mu materials. It is necessary for researchers to conduct pharmacokinetics and pharmacodynamics, which will be beneficial for studying the action mechanism and developing the medicinal value of Bei Mu. A limited number of studies focused on pharmacokinetics and pharmacodynamics, such as the pharmacokinetics of ebeiedinone in mouse blood [132] and pharmacokinetic study of delavinone in mice [133].

## Toxicity

The dried bulbs of *Fritillaria* exhibit an extremely low toxicity. Li et al. [134] have summarized the toxicity properties of *F. thunbergii*, one of main medicinal herbs of Fritillaria, which indicated the dosage recorded in Pharmacopeia of China is safe and tremor and reduction of spontaneous motor activities were found in the 3 mg/kg group. The estimated median lethal dosage (LD_50_) of the water extract of *F. thunbergii* was 52.2 mg kg^−1^ body weight of mice in the acute toxicity tests, while a dose of 1 mg kg^−1^ body weight was no toxicity in the sub-chronic toxicity tests. The main adverse symptom was observed in body or head tremor and spontaneous motor activity reduction when the dosage is above the 1 mg kg^−1^ dose in male rats. There was no significant fluctuation observed in hematology, blood biochemistry, organ weight and organ histology [135]. The comparison analysis of acute toxicity between *F. cirrhosa* and *F. pallidiflora* indicated that the LD_50_ of the latter was 213.57 g kg^−1^ body weight, whereas the maximum feasible dose of the former was 452.14 g kg^−1^ in the studied mice. A histopathological analysis was performed, and the results reflect that inflammatory cell infiltration and cell edema in the liver, multinucleated giant cell proliferation in spleen, perivascular exudate and hemorrhage in lungs, and glomerulus atrophy in the kidney of mice after oral administrations of *F. pallidiflora* extracts. Only liver cell edema was observed in the *F. cirrhosa* group [80].

In addition, *Fritillaria* species have certain cytotoxicity to various tumor cells. The aqueous extract of *F. cirrhosa* induced mitotic aberrations and chromosomal instability in human colon epithelial NCM460 cells via the dysfunction pathway of mitotic checkpoint and cytokinesis failure [136, 137]. The aqueous and ethanolic extracts of *F. imperialis* presented cytotoxic, cytostatic, and pro-apoptotic activities against human liver cancer cells and breast cancer cells. The ethanolic extract was more potent than the aqueous extract [138].

## Analytical techniques for chemical evaluation of *Fritillaria* species

### Gas chromatography

Gas chromatography (GC) and its combination approach with mass spectrometry is a powerful tool for phytochemical analysis because of their high separation efficiency and short analysis time. The isosteroidal alkaloids in bulbs of *Fritillaria* were identified and determined by GC [81, 139] and GC–MS with pre-column derivatization [140]. However, given the polarity of isosteroidal alkaloids, they cannot be eluted from conventional GC columns, and derivatization processes are required prior to GC analysis. The constituents of volatile oil of *F. cirrhosa* were identified by GC–MS [69]. The metabolomic multivariable analysis using GC–MS dataset, including principle component analysis, partial least square discriminate analysis (PLS-DA), orthogonal PLS-DA, and heat map analysis were applied to assess the quality, distinguishing bulbs, stems, leaves, and flowers of *F. thunbergii* [141]. Moreover, 3-methyl-2-butene-1-thiol leading to the foxy odor in *F. imperialis* was identified using dynamic headspace gas chromatography–olfactometry and GC–MS [142].

### Liquid chromatography

High performance liquid chromatography (HPLC) equipped with various detector is a conventional technique applied to determine the isosteroidal alkaloids in the dried bulbs of *Fritillaria*, in addition to evaporative light scattering detection (ELSD) [143–149] and charged aerosol detector [150] used for the sulfur-fumigation process. Nucleosides and nucleobases in *Fritillaria* bulbs have been mentioned in the above chapters and these components in different *Fritillaria* species were simultaneously determined by a HPLC diode-array detector (DAD) [151–153]. In addition, HPLC coupled with an ELSD and DAD was utilized for the quantitative analysis of four alkaloids and nine nucleosides and nucleobases from *F. taipaiensis* that had been cultivated in the same field for 2–6 years [154]. The overall quality evaluation was established via the combination strategy between HPLC fingerprint and chemometrics [155]. What’s more, there are both HPLC fingerprints and DNA barcoding combined to analyze *Fritillaria* species [156]. In general, ELSD is not a commonly utilized detector, whereas the qualitative and quantitative analyses of isosteroidal alkaloids by conventional LC with DAD detector is difficult given the lack of chromophore groups. Isosteroidal alkaloid derivatives are suitable for ultraviolet and fluorescent determination, showing a high detection sensitivity and meeting the requirements of analysis. Therefore, the development of derivatization reagents is a key to the quality control of isosteroidal alkaloids of *Fritillaria*.

Liquid chromatography with several mass spectrometry [triple quadrupole (QQQ), ion trap (IT), and time-of-flight (TOF)] has been extensively used in the authentication and isolation of chemical components of herbal medicine because of its high efficiency and accurate identification. The combination technique can be comprehensively applied to analyze those components with different polarity, equipped with atmospheric pressure chemical ionization (APCI) and electrospray ionization (ESI). Although LC–MS can be used to analyze trace components, good sample pretreatment, including extraction and enrichment, is the key to discover trace active components. The steroidal alkaloids in *Fritillaria* species were identified and characterized using LC/ESI-TOF-MS [157–159], HPLC–MS method [158, 160], LC-ESI-QQQ-MS [159, 161], RRLC-Q-TOF-MS coupled with multivariate statistical analysis [162], and UPLC-QTOF-MS [163, 164] with metabolomic strategy, which were used to determine potential markers for quality control at different growth stages [165]. In addition, the major alkaloids in *F. hupehensis* were determined by HPLC-ELSD and HPLC-ESI-MS^n^ [166]. A new ionization technique, that is, wooden-tip ESI/MS has been applied to the chemical investigation of *Fritillaria* species. Wooden-tip ESI/MS [167], UPLC-Q-TOF/MS, and UPLC-TQ/MS [168], and matrix-assisted laser desorption/ionization mass spectrometry [169] combined with multivariate statistical analysis had been applied to discriminate officinal species of Fritillariae Bulbus and adulterated samples [167]. Alkaloids and flavonoids in five botanical parts (flowers, flower buds, stems, leaves, and bulbs) of *F. thunbergii* and isosteroidal alkaloids in vitro propagation of *F. cirrhosa* bulbs were identified by LC-LTQ-Orbitrap MS^n^ [170] and LC-LTQ MS^n^ [171], respectively. Other kinds of nucleosides and nucleobases were also authenticated and determined by HPLC–ESI–MS [172].

Pharmacokinetic investigations by LC–MS focused on isosteroidal alkaloids (such as peimine, peiminine, verticine, verticinone and isoverticine, puqietinone, imperialine, delavinone, and ebeiedinone) in rat [173–177], mouse [132, 133], and beagle dog [178]. Studies had also been conducted to investigate the tissue distribution and excretion of two components (peimine and peiminine) in rats on the basis of pharmacokinetics [179].

Apart from the analytical techniques mentioned above, a spectroscopy analytical method reflecting integrated metabolic information was used for the chemical assessment of *Fritillaria* species. The two-dimensional correlation analysis of Fourier transform-near infrared spectroscopy (NIR) has been successfully applied to quality control and assessment of Fritillariae Bulbus combined with scientific chemometrics [180]. NIR hyperspectral imaging is also a rapid and non-destructive technique to detect SO_2_ residual in the dried bulbs of *F. thunbergii*. Target analysis using chromatography is a quantitative analytical method, whereas non-deductive spectroscopy is a promising qualitative approach for further chemical assessment of *Fritillaria*.

## Conclusion and future perspective

Geographical distribution has shown that *Fritillaria* species are naturally distributed in Asian and Europe, with huge species diversity (Fig. 1). Immature cultivation techniques cannot meet the entire market demand, especially those for *F. cirrhosa* and *F. roylei*, even though there has been a large cultivated scale of other species. Therefore, comprehensive physiological and ecological research should focus on artificial breeding, which will also protect the wild resources from excessive excavation. Modern molecular assisted breeding may become an important topic for overcoming the cultivation obstacle of several high-profit species avoid the long growth period of their perennial counterparts.

There is no doubt that alkaloids are the prominent constituents in the medicinal parts of *Fritillaria* species, and they have become the evaluation index for ensuring whether the target objective can be used as a substitution for the endangered medicinal Bei Mu. However, natural herbal medicines are inherently complex mixtures, with varying constituents depending on species origins or botanical parts, and different from their pharmaceutical counterparts. In addition, the quantities and identities of the authenticated components are unclear. Moreover, pharmacological activity may be synergetic variation with these factors. It is worthwhile to note that some degradation and combination reactions occur during boiling and metabolism in the body and are therefore promising for investigation in the future experimental or clinical studies. Other non-alkaloids are also important constituents for medicinal purposes. The chemically modified components or gold nanoparticles, such as polysaccharide-zinc complex of *F. ussuriensis* [181] and *F. cirrhosa* gold nanoparticles [182], show a promising tendency, that the amount of substitution zinc determined the antioxidant activity. There are little research focusing the structure modification of chemical components isolated from *Fritillaria*, which should raise more attention in the further analytical study. From a serious perspective, the way in which the original plants are classified and authenticated is more important than the relative products from which they are prepared, and much of the research investigated in the further should be paid more attention, regardless of their similar chemical components.

The extensive pharmacological activity and clinical application value show the development and utilization potential of the bulbs *Fritillaria*. However, their mechanisms of action are still unclear. Therefore, modern scientific methods should be used to further study the pharmacological actions to clarify the multi-target and multi-channel mechanism of Bei Mu materials. The pharmacological investigation should focus on the potential metabolism of the separated components in future, which have been conducted the detailed research in animals, and explore the relationship between their pharmacokinetics and pharmacodynamics, in addition, new dosage forms, administration methods, such as the research and development of nano-preparations and inhalation preparations, and *Fritillaria* materials should be developed and applied.

## Supplementary Information

**Additional file 1: Table S1.** Traditional usage in national minorities of China. **Table S2.** Traditional usage of *Fritillaria* species in different countries. **Table S3.** Distribution information of alkaloids in *Fritillaria* species. **Table S4.** Distribution information of terpenoids in *Fritillaria* species. **Table S5.** Distribution information of steroidal saponins in *Fritillaria* species. **Table S6.** Distribution information of phenylpropanoids in *Fritillaria* species. **Table S7.** Distribution information of fatty acids in *Fritillaria* species. **Table S8.** Distribution information of steride**s** in *Fritillaria* species. **Table S9.** Distribution information of other components in *Fritillaria* species. **Table S10.** Pharmacological activities of *Fritillaria* species.

## Data Availability

Data sharing is not applicable to this article as no datasets were generated or analyzed during the current study.

## Abbreviations

COPD: Chronic obstructive pulmonary disease
COVID-19: Coronavirus disease 19
BC: Before Christ
AD: Anno Domini
ALI: Acute lung injury
LPS: Lipopolysaccharide
MUC5AC: Mucin 5 subtype AC
GBM: Glioblastoma
NSCLC: Non-small cell lung cancer
CaMKII: Ca^2^^+^/calmodulin-dependent protein kinase II
JNK: C-Jun N-terminal kinase
Nrf2: Nuclear factor 2
ACE: Angiotensin converting enzyme
AchE: Acetylcholinesterase
BchE: Butyrylcholinesterase
LD_50_: Lethal dosage
GC: Gas chromatography
MS: Mass spectra
HPLC: High performance liquid chromatography
ELSD: Evaporative light scattering detection
DAD: Diode-array detector
QQQ: Triple quadrupole
IT: Ion trap
TOF: Time-of-flight
APCI: Atmospheric pressure chemical ionization
ESI: Electrospray ionization
PLS-DA: Partial least square discriminate analysis
NIR: Near infrared spectroscopy
