# Supplementary material for: Natural drug sources for respiratory diseases from Fritillaria: chemical and biological analyses
Source: Chin Med. 2021 May 31;16:40. doi: 10.1186/s13020-021-00450-1 (PMC8165352; doi:10.1186/s13020-021-00450-1)
Supplement: Supplementary file 1 — Additional file 1: Table S1. Traditional usage in national minorities of China. Table S2. Traditional usage of Fritillaria species in different countries. Table S3. Distribution information of alkaloids in Fritillaria species. Table S4. Distribution information of terpenoids in Fritillaria species. Table S5. Distribution information of steroidal saponins in Fritillaria species. Table S6. Distribution information of phenylpropanoids in Fritillaria species. Table S7. Distribution information of fatty acids in Fritillaria species. Table S8. Distribution information of sterides in Fritillaria species. Table S9. Distribution information of other components in Fritillaria species. Table S10. Pharmacological activities of Fritillaria species. [file 13020_2021_450_MOESM1_ESM.docx]

**Natural drug sources for respiratory diseases from *Fritillaria*: chemical and biological analyses**

Table S1 Traditional usage in national minorities of China

| Scientific name | National minority | Efficacy |
| --- | --- | --- |
| *F. cirrhosa* D. Don | Tibetan | The bulbs were used for curing tracheitis, menometrorrhagia, the leaves were used for Huangshui disease, and the seeds were used for some disease at head and deficiency-heat symptom. |
|  | Mongolian | The bulbs were used for chest heat and pain, Yin deficiency and dry cough, expectoration with blood, lung heat and cough of children. |
|  | Miao | The bulbs were used for dry cough because of Yin deficiency and expectoration with blood. |
|  | Lisu | The bulbs were used for treatment of cough under fatigue, expectoration with blood, cardiothoracic depression, lung wilt, lung carbuncle, tumor, breast carbuncle. |
|  | Tujia | The bulbs were used for cough, tracheitis, hemoptysis. |
| *F. delavayi* Franch | Tibetan | The bulbs were used for curing poisoning and cough because of lung heat, the leaves were used for Huangshui disease, and the seeds were used for some disease at head and deficiency-heat symptom. |
|  | Mongolian | The same as those of *F. cirrhosa.* |
| *F. unibracteata* Hsiao et K.C. Hsia | Tibetan | The same as those of *F. delavayi.* |
|  | Mongolian | The bulbs were used for treatment of chest heat and pain, dry cough because of Yin deficiency, expectoration with blood, and cough caused by lung heat in children. |
|  | Miao | The same as those of *F. cirrhosa.* |
| *F. karelinii* Baker | Kazakh | The bulbs were used for chronic bronchitis and upper respiratory infection of children. |
|  | Uighur | The bulbs were used for treatment of chronic cough asthma. |
| *F. maximowiczii* Freyn | Mongolian | The bulbs were used for curing cough leaded by lung heat, lung tingling, chronic bronchitis, asthma, indigestion, cold. |
| *F. pallidiflora* Schrenk | Mongolian | The same as those of *F. maximowiczii.* |
|  | Kazakh | The bulbs were used for urgent/chronic bronchitis and upper respiratory infection of children. |
|  | Uighur | The bulbs were used for treatment of chronic cough asthma and carbuncle cough. |
| *F. thunbergii* Miq. | Mongolian | The bulbs were used for curing cure colds, coughs, ulcers, lymph node tuberculosis, carbuncle. |
|  | Jingpo | Bulb cures upper respiratory tract infection, bronchitis, lung abscess, gastric and duodenal ulcer, goiter. |
|  | Deang | The same as those of Jingpo of the species |
| *F. ussuriensis* Maxim | Korean | The bulbs were used for lung deficiency, cough, cough due to exogenous wind-heat, phlegm depression. |
| *F. walujewii* Regel | Kazakh | The same as those of *F. pallidiflora.* |
|  | Uighur | The same as those of *F. pallidiflora.* |

Table S2 Traditional usage of *Fritillaria* species in different countries.

| Scientific name | Local names | Medicinal part and native country | Traditional/ethnopharmacological records | References |
| --- | --- | --- | --- | --- |
| *F. anhuiensis* S.C. Chen et S.F. Yin | An Hui Bei Mu (Chinese) | Bulbs, China | Be used for coughs, asthma, bronchitis | [1] |
| *F.* *cirrhosa* D. Don | Chuan Bei Mu (Chinese); Sen-Baimo (Japanese) | Bulbs, China | Be used for treating respiratory diseases such as cough, expectoration and asthma. | [2] |
| *F.* *delavayi* Franch |  |  |  |  |
| *F.* *unibracteata* Hsiao et K.C. Hsia |  |  |  |  |
| *F. unibracteata* var*. wabuensis* |  |  |  |  |
| *F.* *ebeiensis* G.D. Yu et G.Q. Ji | E Bei Bei Mu (Chinese) | Bulbs, China | Be used as Chinese folk medicine for clearing heat, moisturizing lungs, and throat diseases | [3] |
| *F. ebeiensis* var. *purpurea* G.D. Yu et P. Li | Zi Hua E Bei Bei Mu (Chinese) | Bulbs, China | Similar to that of *F. ebeiensis* | [4] |
| *F. hupehensis* Hsiao et K.C. Hsia | Hu Bei Bei Mu (Chinese) | Bulbs, China | -- |  |
| *F.* *imperialis* L. | Ashk-e-maryam (Farsi) | Bulbs, Iran | Be used as an anti-cough and spasmodic drug, and for  treatment of various diseases such as asthma, bronchitis, Alzheimer, sciatica and wound healing | [5] |
|  | -- | Bulbs, Turky | Be used for the treatment of various ailments such as sore throat, cough, asthma, bronchitis, scrofula, gland tumor, dysuria and haemoptysis. | [6] |
| *F.* *karelinii* Baker | Sha Bei Mu | Bulbs, China | Similar to Chuan Bei Mu | [7] |
| *F.* *lichuanensis* P. Li et C.P. Yang | Li Chuan Bei Mu (Chinese) | Bulbs, China | -- | [8] |
| *F.* *maximowiczii* Freyn | Rinyou-Baimo (Janpanese) | Bulbs, China | A substitute for the bulbs of other *Fritillaria* species | [9] |
| *F.* *meleagris* L. | Snake’s head fritillary | Bulbs, Asian and northwestern Europe | -- | [10] |
| *F.* *michailovskyi* Fomin | -- | Bulbs, Turkey | -- | [11] |
| *F.* *monatha* Migo | Peng Ze Bei Mu | Bulbs, China | Similar to Chuan Bei Mu | [12] |
| *F. ningguoensis* S.C. Chen et S.F. Yin | Ning Guo Bei Mu | Bulbs, China | Similar to Chuan Bei Mu and Zhe Bei Mu | [13] |
| *F.* *pallidiflora* Schrenk | Yi Bei Mu | Bulbs, China | Be used for antitussive, antiasthmatic, and expectorant drugs | [14] |
| *F.* *puqiensis* G.D. Yu et G.Y. Chen | Pu Xi Bei Mu | Bulbs, China | Similar to Bei Mu | [15] |
| *F.* *roylei* Hooker | -- | Bulbs, Western temperate Himalaya from Kashmir to Kumaon | Used in the treatment of asthma, bronchitis and tuberculosis | [16] |
| *F.* *shuchengensis* S.C. Chen et S.F. Yin | An Hui Bei Mu, Shu Cheng Bei Mu | Bulbs, China | Antitussive | [17] |
| *F.* *taipaiensis* var. *ningxiaensis* Y.K. Yang et J.K. Wu | Ning Xia Bei Mu | Bulbs, China | Be used for cough, expectoration | [18] |
| *F.* *thunbergii* Miq. | Zhe Bei Mu (Chinese), Setu-Baimon (Janpanese) | Bulbs, China | Be used for clearing heat, nourishing lung, removing phlegm, cough, expectoration | [9] |
| *F. thunbergii* var*.* *chekiangensis* | Dong Bei Mu, Dongyang Bei Mu | Bulbs, China | Similar to Zhe Bei Mu | [19] |
| *F.* *tortifolia* X.Z. Duan et X.J. Zheng | Tuo Li Bei Mu | Bulbs, China | Similar to Bei Mu | [20] |
| *F.* *ussuriensis* Maxim | Ping Bei Mu | Bulbs, China | Similar to Chuan Bei Mu | [21] |
| *F. walujewii* Regel | Xin Jiang Bei Mu | Bulbs, China | Be used for clearing heat, nourishing lung, removing phlegm, cough, expectoration, removing stasis | [22] |
| *F. yuminensis* X.Z. Duan | Yumin Bei Mu | Bulbs, China | Be used for antitussive, antiasthmatic, and expectorant drugs | [23] |

Table S3 Distribution information of alkaloids in *Fritillaria* species

| Name | Species | Botanical parts | Types | References |
| --- | --- | --- | --- | --- |
| 1 Pingpeimine A | *F.* *ussuriensis* Maxim | Bulbs, stems, leaves, flowers | A1 | [24,25,26] |
| 2. peimine (verticine) | *F. ussuriensis* Maxim | Bulbs, stems, leaves, flower | A1 | [24,25,27] |
|  | *F. thunbergii* Miq. | Aerial parts and bulbs |  | [28] |
|  | *F. delavayi* Franch. | Bulbs |  | [29] |
|  | *F. anhuiensis* S.C. Chen et S.F. Yin | Bulbs |  | [1] |
|  | *F. ningguoensis* S.C. Chen et S.F. Yin | Bulbs |  | [13] |
|  | *F. hupehensis* Hsiao et K.C. Hsia | Bulbs |  | [30] |
|  | *F. ebeiensis* G.D. Yu et G.Q. Ji | Bulbs |  | [31] |
|  | *F. thunbergii* var. *chekiangensis* | Bulbs |  | [19] |
|  | *F. ebeiensis* var. *purpurea* G.D. Yu et P. Li | Bulbs |  | [32] |
|  | *F.* *monantha* Migo | Bulbs |  | [33] |
|  | *F. cirrhosa* D. Don | Bulbs |  | [34] |
|  | *F. unibracteata* var. *wabuensis* | Bulbs |  | [35] |
| 3 Pingpeimine B | *F. ussuriensis* Maxim | Stems, leaves, bulbs | A1 | [21,36] |
| 4 Isoverticine | *F. taipaiensis* var. *ningxiaensis* Y.K. Yang et J.K. Wu | Bulbs | A1 | [13,18] |
|  | *F. unibracteata* var. *wabuensis* | Bulbs |  | [37] |
|  | *F. anhuiensis* S.C. Chen et S.F. Yin | Bulbs |  | [1] |
|  | *F. ningguoensis* S.C. Chen et S.F. Yin | Bulbs |  | [13] |
|  | *F. thunbergii* var. *chekiangensis* | Bulbs |  | [19] |
| 5 Zhebeinine | *F. thunbergii* Miq. | Bulbs | A1 | [38] |
|  | *F. hupehensis* Hsiao et K.C. Hsia | Bulbs |  | [38] |
| 6 Yibeinoside A (delavinone, sinpeinine A) | *F. pallidiflora* Schrenk | Bulbs | A1 | [39] |
|  | *F. walujewii* Regel | Bulbs |  | [40] |
|  | *F. puqiensis* G.D. Yu et G.Y. Chen | Bulbs |  | [41] |
|  | *F. delavayi* Franch. | Bulbs |  | [29,42] |
|  | *F. tortifolia* X.Z. Duan et X.J. Zheng | Bulbs |  | [43] |
|  | *F. yuminensis* X.Z. Duan | Bulbs |  | [23] |
|  | *F. cirrhosa* D. Don | Bulbs |  | [34] |
| 7 Taipaienine | *F. taipaiensis* var. *ningxiaensis* Y.K. Yang et J.K. Wu | Bulbs | A1 | [18,44] |
| 8 Chuanbeinone | *F. taipaiensis* var. *ningxiaensis* Y.K. Yang et J.K. Wu | Bulbs | A1 | [18] |
|  | *F. delavayi* Franch. | Bulbs |  | [29,45] |
|  | *F. cirrhosa* D. Don | Bulbs |  | [34] |
|  | *F. unibracteata* var. *wabuensis* | Bulbs |  | [35] |
| 9 Eduardine (ebeiedinone) | *F. thunbergii* Miq. | Bulbs | A1 | [46] |
|  | *F. puqiensis* G.D. Yu et G.Y. Chen | Bulbs |  | [41] |
|  | *F. anhuiensis* S.C. Chen et S.F. Yin | Bulbs |  | [41] |
|  | *F. ussuriensis* Maxim | Bulbs |  | [27] |
|  | *F. tortifolia* X.Z. Duan et X.J. Zheng | Bulbs |  | [43] |
| 10 Puqiedinone | *F. puqiensis* G.D. Yu et G.Y. Chen | Bulbs | A1 | [47] |
| 11 Imperialine (sipeimine) | *F. ussuriensis* Maxim | Bulbs | A1 | [26] |
|  | *F. pallidiflora* Schrenk | Bulbs |  | [48] |
|  | *F. delavayi* Franch. | Bulbs |  | [42] |
|  | *F. taipaiensis* var. *ningxiaensis* Y.K. Yang et J.K. Wu | Bulbs |  | [18] |
|  | *F. walujewii* Regel | Bulbs |  | [40] |
|  | *F. unibracteata* var. *wabuensis* | Bulbs |  | [49] |
|  | *F. roylei* Hooker | Bulbs |  | [50] |
|  | *F. tortifolia* X.Z. Duan et X.J. Zheng | Bulbs |  | [43] |
|  | *F. yuminensis* X.Z. Duan | Bulbs |  | [23] |
|  | *F. hupehensis* Hsiao et K.C. Hsia | Bulbs |  | [30] |
|  | *F. cirrhosa* D. Don | Bulbs |  | [34] |
| 12 Pingpeimine C | *F. ussuriensis* Maxim | Stems, leaves,  bulbs | A1 | [36,51] |
| 13 Verticinone (peiminine, zhebeinone) | *F. taipaiensis* var. *ningxiaensis* Y.K. Yang et J.K. Wu | Bulbs | A1 | [18] |
|  | *F. thunbergii* Miq. | Aerial parts and bulbs |  | [28] |
|  | *F. lichuanensis* P. Li et C.P. Yang | Bulbs |  | [8] |
|  | *F. delavayi* Franch. | Bulbs |  | [29] |
|  | *F. hupehensis* Hsiao et K.C. Hsia | Bulbs, stems and leaves |  | [52] |
|  | *F. anhuiensis* S.C. Chen et S.F. Yin | Bulbs |  | [1] |
|  | *F. ningguoensis* S.C. Chen et S.F. Yin | Bulbs |  | [13] |
|  | *F. ebeiensis* G.D. Yu et G.Q. Ji | Bulbs |  | [31] |
|  | *F. ebeiensis* var. *purpurea* G.D. Yu et P. Li | Bulbs |  | [32] |
|  | *F. thunbergii* var. *chekiangensis* | Bulbs |  | [19] |
|  | *F. monantha* Migo | Bulbs |  | [33] |
|  | *F. cirrhosa* D. Don | Bulbs |  | [34] |
|  | *F. unibracteata* var. *wabuensis* | Bulbs |  | [35] |
| 14 Hupehenirine | *F. hupehensis* Hsiao et K.C. Hsia | Bulbs | A1 | [53] |
|  | *F. lichuanensis* P. Li et C.P. Yang | Bulbs |  | [8] |
| 15 Forticine | *F. imperialis* L. | Bulbs | A1 | [54] |
| 16 Persicanidine | *F. imperialis* L. | Bulbs | A1 | [54] |
| 17 Puqiedine | *F. puqiensis* G.D. Yu et G.Y. Chen | Bulbs | A1 | [55] |
| 18 Impericine | *F. imperialis* L. | Bulbs | A1 | [54] |
| 19 Hupehenizine | *F. hupehensis* Hsiao et K.C. Hsia | Bulbs | A1 | [53] |
|  | *F. lichuanensis* P. Li et C.P. Yang | Bulbs |  | [8] |
| 20 Imperialine-*β*-N-oxide | *F. pallidiflora* Schrenk | Bulbs | A1 | [48] |
|  | *F. unibracteata* var. *wabuensis* | Bulbs |  | [37] |
| 21 Isoverticine-*β*-*N*-oxide | *F. unibracteata* var. *wabuensis* | Bulbs | A1 | [56] |
| 22 Delavine | *F. delavayi* Franch. | Bulbs | A1 | [42] |
|  | *F. imperialis* L. | Bulbs |  | [54] |
| 23 Hupehenine (5α,14α-cevanine-3α,6α-diol) | *F. hupehensis* Hsiao et K.C. Hsia | Bulbs, stems and leaves | A1 | [52,53] |
|  | *F. pallidiflora* Schrenk | Bulbs |  | [57] |
|  | *F. monantha* Migo | Bulbs |  | [33] |
| 24 Hupehenizioiside | *F. lichuanensis* P. Li *et* C.P. Yang | Bulbs | A1 | [8] |
|  | *F. tortifolia* X. Z. Duan et X. J. Zheng | Bulbs |  | [43] |
|  | *F. yuminensis* X.Z. Duan | Bulbs |  | [23] |
| 25 Hupeheninoside | *F. lichuanensis* P. Li *et* C.P. Yang | Bulbs | A1 | [8] |
| 26 Ebeinone | *F. unibracteata* var. *wabuensis* | Bulbs | A1 | [37] |
|  | *F. ebeiensis* G.D. Yu et G.Q. Ji | Bulbs |  | [31] |
|  | *F. ebeiensis* var. *purpurea* G.D. Yu et P. Li | Bulbs |  | [32] |
| 27 3α-puqiedin-7-ol | *F. puqiensis* G.D. Yu et G.Y. Chen | Bulbs | A1 | [55] |
| 28 Zhebeininoside | *F. shuchengensis* S.C. Chen et S.F. Yin | Bulbs | A1 | [17] |
| 29 3-*O*-acetylverticine ((3*β*,5α,6α)-6,20-dihydroxycevan-3-yl acetate) | *F. hupehensis* Hsiao et K.C. Hsia | Bulbs | A1 | [38] |
| 30 Ebeinine | *F. hupehensis* Hsiao et K.C. Hsia | Bulbs | A1 | [58] |
|  | *F. tortifolia* X.Z. Duan et X.J. Zheng | Bulbs |  | [43] |
|  | *F. ebeiensis* G.D. Yu et G.Q. Ji | Bulbs |  | [31] |
|  | *F. ebeiensis* var. *purpurea* G.D. Yu et P. Li | Bulbs |  | [32] |
| 31 Impranine | *F. imperialis* L. | Bulbs | A2 | [6] |
| 32 Dihydroimpranine | *F. imperialis* L. | Bulbs | A2 | [6] |
| 33 Korsevine | *F. imperialis* L. | Bulbs | A2 | [6] |
| 34 Puqienine A | *F. puqiensis* G.D. Yu et G.Y. Chen | Bulbs | A2 | [15] |
| 35 Puqienine B | *F. puqiensis* G.D. Yu et G.Y. Chen | Bulbs | A2 | [15] |
| 36 Puqienine F | *F. puqiensis* G.D. Yu et G.Y. Chen | Bulbs | A2 | [59] |
| 37 Puqienine C | *F. puqiensis* G.D. Yu et G.Y. Chen | Bulbs | A2 | [55] |
| 38 Puqienine D | *F. puqiensis* G.D. Yu et G.Y. Chen | Bulbs | A2 | [55] |
| 39 Puqienine E | *F. puqiensis* G.D. Yu et G.Y. Chen | Bulbs | A2 | [55] |
| 40 Puqietinedione | *F. puqiensis* G.D. Yu et G.Y. Chen | Bulbs | B1 | [55] |
| 41 (3*β*,5*α*,13*α*,23*β*)-7,8,12,14-tetradehydro-5,6,12,13-tetrahydro-3,23-dihydroxyveratraman-6-one | *F. hupehensis* Hsiao et K.C. Hsia | Bulbs | A2 | [38] |
| 42 (3*β*,5*α*,13*α*,23*β*)-7,8,12,14-tetradehydro-5,6,12,13-tetrahydro-3,13,23-trihydroxyveratraman-6-one | *F. hupehensis* Hsiao et K.C. Hsia | Bulbs | A2 | [38] |
| 43 3*β*,23*β*-dihydroxy-7,12(14)-dien-5α-veratramin-6-one | *F. hupehensis* Hsiao et K.C. Hsia | Bulbs | A2 | [58] |
| 44 Peimisine (ebeiensine) | *F. ussuriensis* Maxim | Bulbs, stems, leaves, flower | A3 | [24,25,26] |
|  | *F. pallidiflora* Schrenk | Bulbs |  | [48,60] |
|  | *F. taipaiensis* var. *ningxiaensis* Y.K. Yang et J.K. Wu | Bulbs |  | [18] |
|  | *F. puqiensis* G.D. Yu et G.Y. Chen | Bulbs |  | [55] |
|  | *F. delavayi* Franch. | Bulbs |  | [29] |
|  | *F. anhuiensis* S.C. Chen *et* S.F. Yin | Bulbs |  | [1] |
|  | *F. tortifolia* X.Z. Duan et X.J. Zheng | Bulbs |  | [43] |
|  | *F. walujewii* Regel | Bulbs |  | [61] |
|  | *F. ningguoensis* S.C. Chen et S.F. Yin | Bulbs |  | [13] |
|  | *F. hupehensis* Hsiao et K.C. Hsia | Bulbs |  | [30] |
|  | *F. thunbergii* Miq. | Bulbs |  | [62] |
|  | *F. monantha* Migo | Bulbs |  | [33] |
|  | *F. cirrhosa* D. Don | Bulbs |  | [34] |
|  | *F. unibracteata* var. *wabuensis* | Bulbs |  | [35] |
| 45 Yibeissine | *F. pallidiflora* Schrenk | Bulbs | A3 | [60] |
| 46 Cyclopamine | *F. pallidiflora* Schrenk | Bulbs | A3 | [48] |
| 47 Cycloposine | *F. pallidiflora* Schrenk | Bulbs | A3 | [48] |
| 48 Kuroyurinidine | *F. maximowiczii* Freyn | Bulbs | A3 | [9] |
|  | *F. yuminensis* X.Z. Duan | Bulbs |  | [23] |
|  | *F. camtschatcensis* | Bulbs |  | [63] |
| 49 23-isokuroyurinidine | *F. maximowiczii* Freyn | Bulbs | A3 | [9] |
| 50 Hupehenisine | *F. hupehensis* Hsiao et K.C. Hsia | Bulbs, stems and leaves | A3 | [52,53] |
| 51 Songbeisine | *F. walujewii* Regel | Bulbs | A3 | [64] |
| 52 Pengbeimine B | *F. monatha* Migo | Bulbs | A3 | [65] |
| 53 Pengbeimine D | *F. monatha* Migo | Bulbs | A3 | [65] |
| 54 Pengbeisine A | *F. monatha* Migo | Bulbs | A3 | [66] |
|  | *F. yuminensis* X.Z. Duan | Bulbs |  | [23] |
| 55 Pengbeisine B | *F. monatha* Migo | Bulbs | A3 | [66] |
| 56 (22S,25S)-solanid-5,20(21)-dien-3*β*-ol | *F. anhuiensis* S.C. Chen et S.F. Yin | Bulbs | B2 | [67] |
| 57 Solanidine | *F. thunbergii* Miq. | Aerial parts | B2 | [68,69] |
|  | *F. camtschatcensis* | Bulbs and aerial part |  | [70] |
| 58 (22S,25S)-solanid-5-en-3*β*-ol | *F. anhuiensis* S.C. Chen et S.F. Yin | Bulbs | B2 | [67] |
| 59 Pingbeinine | *F. pallidiflora* Schrenk | Stems, leaves | B1 | [71] |
| 60 Delavidine | *F. delavayi* Franch. | Bulbs | B1 | [29] |
| 61 Puqietinone | *F. puqiensis* G.D. Yu et G.Y. Chen | Bulbs | B1 | [72] |
| 62 N-demethylpuqietinone | *F. puqiensis* G.D. Yu et G.Y. Chen | Bulbs | B1 | [15] |
| 63 Puqietinonoside | *F. puqiensis* G.D. Yu et G.Y. Chen | Bulbs | B1 | [15] |
| 64 Fetisinine | *F. imperialis* L. | Bulbs | B1 | [6] |
| 65 Puqiedinone-3-*O*-*β*-D-glucopyranoside | *F. unibracteata* Hsiao et K. C. Hsia | Bulbs | A1 | [73] |
| 66 Peimisine-3-*O*-*β*-D-glucopyranoside | *F. unibracteata* Hsiao et K. C. Hsia | Bulbs | A3 | [73] |
|  | *F. yuminensis* X.Z. Duan | Bulbs |  | [23] |
| 67 Verticinedinone | *F. anhuiensis* S.C. Chen et S.F. Yin | Bulbs | A1 | [1] |
|  | *F. thunbergii* var*. chekiangensis* | Bulbs |  | [19] |
| 68 Pengbeimine A | *F. anhuiensis* S.C. Chen et S.F. Yin | Bulbs | A3 | [1] |
|  | *F. monatha* Migo | Bulbs |  | [74] |
| 69 Verticinone-N-oxide | *F. shuchengensis* S.C. Chen et S.F. Yin | Bulbs | A1 | [17] |
| 70 3-*O*-acetoxyverticinone ((3*β*,5α)-20-hydroxy-6-oxocevan-3-yl cetate) | *F. hupehensis* Hsiao et K.C. Hsia | Bulbs | A1 | [38] |
| 71 Suchengbeisine | *F. shuchengensis* S.C. Chen et S.F. Yin | Bulbs | A2 | [17] |
| 72 Pingbeimunone A | *F. ussuriensis* Maxim | Bulbs | A2 | [27] |
| 73 Ussuriedine | *F. ussuriensis* Maxim | Bulbs | Other | [27] |
| 74 Benzo(7,8) fluoreno(2,1-*β*)quinolizine cevane-3,6,16,20-tetrol | *F. ussuriensis* Maxim | Bulbs | A1 | [27] |
| 75 Pingbeimine C | *F. ussuriensis* Maxim | Bulbs | A1 | [27] |
| 76 5α, 14α, 17*β*-cevanin-6-oxo-3*β*, 20*β*, 24*β*-triol | *F. pallidiflora* Schrenk | Bulbs | A1 | [57] |
| 77 Yubeinine | *F. pallidiflora* Schrenk | Bulbs | A1 | [57] |
|  | *F. tortifolia* X.Z. Duan et X.J. Zheng | Bulbs |  | [43] |
|  | *F. yuminensis* X.Z. Duan | Bulbs |  | [23] |
| 78 Yibeinoside | *F. pallidiflora* Schrenk | Bulbs | A1 | [57] |
| 79 Yibeinone A | *F. pallidiflora* Schrenk | Bulbs | A3 | [75] |
| 80 Yibeinone B | *F. pallidiflora* Schrenk | Bulbs | A2 | [75] |
| 81 Yibeinone C | *F. pallidiflora* Schrenk | Bulbs | A1 | [75] |
| 82 Yibeinone D | *F. pallidiflora* Schrenk | Bulbs | A1 | [75] |
| 83 Dongbeinine | *F. pallidiflora* Schrenk | Bulbs | A1 | [75] |
| 84 Frititorine A | *F. tortifolia* X.Z. Duan et X.J. Zheng | Bulbs | A1 | [43] |
| 85 Frititorine B | *F. tortifolia* X.Z. Duan et X.J. Zheng | Bulbs | A1 | [43] |
| 86 Frititorine C | *F. tortifolia* X.Z. Duan et X.J. Zheng | Bulbs | A3 | [43] |
| 87 Imperialinol | *F. tortifolia* X.Z. Duan et X.J. Zheng | Bulbs | A1 | [43] |
| 88 Imperialine-3-*O*-*β*-D-glucoside | *F. tortifolia* X.Z. Duan et X.J. Zheng | Bulbs | A1 | [43] |
|  | *F. yuminensis* X.Z. Duan | Bulbs |  | [23] |
|  | *F. walujewii* Regel | Bulbs |  | [61] |
|  | *F. unibracteata* var. *wabuensis* | Bulbs |  | [35] |
| 89 Tortifoline | *F. yuminensis* X.Z. Duan | Bulbs | A1 | [23] |
|  | *F. tortifolia* X.Z. Duan et X.J. Zheng | Bulbs |  | [20] |
|  | *F. walujewii* Regel | Bulbs |  | [61] |
| 90 Shinonomenine | *F. tortifolia* X.Z. Duan et X.J. Zheng | Bulbs | A1 | [20] |
| 91 Walujewine A | *F. walujewii* Regel | Bulbs | A3 | [61] |
| 92 Solanidine-3-O-α-L-rhamnopyranosyl-(1→2)-β-D-glucopyranoside | *F. yuminensis* X.Z. Duan | Bulbs | B2 | [23,69] |
| 93 Solanidine-3-O-α-L-rhamnopyranosyl-(1→2)-[β-D-glucopyranosyl-(1→4)]-β-D-glucopyranoside | *F. yuminensis* X.Z. Duan | Bulbs | B2 | [23,69] |
|  | *F. cirrhosa* D. Don | Bulbs |  | [34] |
| 94 Hapepunine | *F. thunbergii* Miq. | aerial parts | B1 | [69] |
| 95 Hapepunine-3-O-α-L-rhamnopyranosyl-(1→2)-β-D-glucopyranoside | *F. thunbergii* Miq. | aerial parts | B1 | [69] |
| 96 Walujewine B | *F. walujewii* Regel | Bulbs | A1 | [61] |
| 97 Walujewine C | *F. walujewii* Regel | Bulbs | A1 | [61] |
| 98 Walujewine D | *F. walujewii* Regel | Bulbs | A1 | [61] |
| 99 Ebeiedine | *F. walujewii* Regel | Bulbs | A1 | [61] |
| 100 Hepehenizioiside | *F. walujewii* Regel | Bulbs | A1 | [61] |
| 101 Walujewine E | *F. walujewii* Regel | Bulbs | A1 | [61] |
| 102 Karelinine | *F. karelinii* Baker | Bulbs | A3 | [7] |
| 103 5-epikarelinine | *F. karelinii* Baker | Bulbs | A3 | [7] |
| 104 27-epiebeienine | *F. karelinii* Baker | Bulbs | A1 | [7] |
| 105 Ebeienine | *F. karelinii* Baker | Bulbs | A1 | [7] |
| 106 Persicanidine B | *F. karelinii* Baker | Bulbs | A1 | [7] |
| 107 Heilonine | *F. karelinii* Baker | Bulbs | A1 | [7] |
| 108 Michainine | *F. michailovskyi* Fomin | Bulbs | B1 | [11] |
| 109 Wanpeinine A | *F. anhuiensis* S.C. Chen et S.F. Yin | Bulbs | A1 | [76] |
| 110 Ningpeisine | *F. ningguoensis* S.C. Chen et S.F. Yin | Bulbs | A2 | [13] |
| 111 Lichuanine ((20S,25R)-5α, 14α-cevanine-3β, 6β-diol) | *F. lichuanensis* P. Li et C.P. Yang | Bulbs | A1 | [77] |
| 112 Lichuanisinine ((20S,25S)-5α,14α-cevanine-3β,6β-diol-N-oxide) | *F. lichuanensis* P. Li et C.P. Yang | Bulbs | A1 | [77] |
| 113 Hupehenidine | *F.* *ebeiensis* G.D. Yu et G.Q. Ji | Bulbs, stems and leaves | other | [31,52] |
| 114 Ziebeimine | *F. ebeiensis* var. *purpurea* G.D. Yu et P. Li | Bulbs | A1 | [32] |
| 115 Yubeiside | *F. yuminensis* X.Z. Duan | Bulbs | A1 | [78] |
| 116 Demissidine [(22R, 25S)-solanidane-3β-ol] | *F. cirrhosa* D. Don | Bulbs | B2 | [79] |
| 117 Petilidine | *F. cirrhosa* D. Don | Bulbs | A1 | [34] |
|  | *F. walujewii* Regel | Bulbs |  | [80] |
| 118 Yibeirine (5α,14α,17α,22β-cevanin-3β,6β,20β-triol) | *F. pallidiflora* Schrenk | Bulbs | A1 | [81] |
| 119 Yibeisine (5α,14α,17β-cevanin-6-oxo-3β,20β,25α-triol) | *F. pallidiflora* Schrenk | Bulbs | A1 | [81] |
| 120 Ussurienine | *F. ussuriensis* Maxim | Bulbs | A1 | [82] |
| 121 Ussurienone | *F. ussuriensis* Maxim | Bulbs | A1 | [82] |
| 122 Ussuriedinone | *F. ussuriensis* Maxim | Bulbs | A1 | [82] |
| 123 Pengbeimine D | *F. monatha* Migo | Bulbs | A2 | [74] |
| 124 Camtschatcanidine | *F. camtschatcensis* | Bulbs | B2 | [70] |

Table S4 Distribution information of terpenoids in *Fritillaria* species

| Name | Species | Botanical parts | References |
| --- | --- | --- | --- |
| 1 (23Z)-9,19-cycloart-23-ene-3α,25-diol | *F. hupehensis* Hsiao et K.C. Hsia | Stems and leaves | [83,84] |
| 2 9,19-cycloart-25-ene-3*β*,24ξ-diol | *F. hupehensis* Hsiao et K.C. Hsia | Stems and leaves | [83,84] |
| 3 Fritillebin C | *F. ebeiensis* G.D. Yu et G.Q. Ji | Bulbs | [85] |
| 4 Fritillebin D | *F. ebeiensis* G.D. Yu et G.Q. Ji | Bulbs | [85] |
| 5 Fritillebinide A | *F. ebeiensis* G.D. Yu et G.Q. Ji | Bulbs | [4,86] |
| 6 Fritillebinide B | *F. ebeiensis* G.D. Yu et G.Q. Ji | Bulbs | [86,87] |
|  | *F. ebeiensis* var*. purpurea* G.D. Yu et P. Li | Bulbs | [4] |
| 7 Fritillebinide C | *F. ebeiensis* G.D. Yu et G.Q. Ji | Bulbs | [87] |
| 8 Isopimara-7,15-dien-19-oic acid | *F. imperialis* | Bulbs | [88] |
| 9 Isopimara-7,15-dien-19-methyl ester | *F. imperialis* | Bulbs | [88] |
| 10 12,15-epoxy-8(17),13-labdadien-19-ol | *F. anhuiensis* S.C. Chen et S.F. Yin | Bulbs | [89] |
| 11 *Ent*-kauran-15-en-17-ol | *F. ebeiensis* G.D. Yu et G.Q. Ji | Bulbs | [90] |
|  | *F. anhuiensis* S.C. Chen et S.F. Yin | Bulbs | [1] |
|  | *F. monanth* Migo | Bulbs | [12] |
| 12 *Ent*-kauran-16*β*,17-diol | *F. ebeiensis* G.D. Yu et G.Q. Ji | Bulbs | [90] |
|  | *F. anhuiensis* S.C. Chen et S.F. Yin | Bulbs | [1] |
|  | *F. hupehensis* Hsiao et K.C. Hsia | Bulbs | [91] |
|  | *F. thunbergii* Miq. | Bulbs | [62] |
| 13 *Ent*-kauran-16α,17-diol | *F. ebeiensis* G.D. Yu et G.Q. Ji | Bulbs | [90] |
|  | *F. anhuiensis* S.C. Chen et S.F. Yin | Bulbs | [1] |
|  | *F. hupehensis* Hsiao et K.C. Hsia | Bulbs | [91] |
|  | *F. monanth* Migo | Bulbs | [12] |
| 14 Fritillebeinol (*ent*-3*β*-acetoxy-kauran-16β,17-diol) | *F. ebeiensis* G.D. Yu et G.Q. Ji | Bulbs | [90] |
| 15 Fritillebin A | *F. ebeiensis* G.D. Yu et G.Q. Ji | Bulbs | [86] |
| 16 Fritillebin B | *F. ebeiensis* G.D. Yu et G.Q. Ji | Bulbs | [86] |
| 17 Fritillebic acid | *F. ebeiensis* G.D. Yu et G.Q. Ji | Bulbs | [86] |
| 18 25-hydroxyl-9,19-cycloart-22-ene-3-one | *F. hupehensis* Hsiao et K.C. Hsia | Stems and leaves | [83] |
| 19 Cycloeucalenol | *F. hupehensis* Hsiao et K.C. Hsia | Stems and leaves | [83] |
| 20 *Ent*-3β-acetoxy-16α-methoxykauran-17-ol | *F. anhuiensis* S.C. Chen et S.F. Yin | Bulbs | [92] |
| 21 12,15-sulfonyl-8(17),13-labdadien-19-oic acid | *F. anhuiensis* S.C. Chen et S.F. Yin | Bulbs | [92] |
| 22 Fritillahupehin | *F. hupehensis* Hsiao et K.C. Hsia | Bulbs | [93,94] |
| 23 Fritillebinide D | *F. ebeiensis* G.D. Yu et G.Q. Ji | Bulbs | [95] |
| 24 Fritillebinide E | *F. ebeiensis* G.D. Yu et G.Q. Ji | Bulbs | [95] |
| 25 *Ent*-3β-butanoyloxykaur-15-en-17-ol | *F. ebeiensis* G.D. Yu et G.Q. Ji | Bulbs | [96] |
| 26 *Ent*-kaur-15-en-17-ol | *F. ebeiensis* G.D. Yu et G.Q. Ji | Bulbs | [96] |
| 27 *Ent*-kaur-15-en-3β,17-diol | *F. ebeiensis* G.D. Yu et G.Q. Ji | Bulbs | [96] |
|  | *F. anhuiensis* S.C. Chen et S.F. Yin | Bulbs | [1] |
| 28 *Ent*-kauran-3β-acetoxy-15-en-17-ol | *F. ebeiensis* G.D. Yu et G.Q. Ji | Bulbs | [96] |
|  | *F. anhuiensis S.C. Chen et S.F. Yin* | Bulbs | [1] |
| 29 6α,7β-dihydroxy-labda-8(17),12(E),14-triene | *F. ebeiensis* G.D. Yu et G.Q. Ji | Bulbs | [3] |
| 30 6-oxo-2α-hydroxy-labda-7,12(E), 14-triene | *F. ebeiensis* G.D. Yu et G.Q. Ji | Bulbs | [3] |
| 31 12R,15-dihydroxy-8(17),13(E)labdadien-19-oic acid | *F. anhuiensis* S.C. Chen et S.F. Yin | Bulbs | [1] |
| 32 12S,15-dihydroxy-8(17),13(E)labdadien-19-oic acid | *F. anhuiensis* S.C. Chen et S.F. Yin | Bulbs | [1] |
| 33 14,15-dihydroxylabda-8(17),12-dien-19-oic acid | *F. anhuiensis* S.C. Chen et S.F. Yin | Bulbs | [1] |
| 34 12R,13RS-dihydroxy-8(17),14-labdadien-19-oic-acid | *F. anhuiensis* S.C. Chen et S.F. Yin | Bulbs | [1] |
| 35 8(17),12,14-labdantrien-18-ol | *F. anhuiensis* S.C. Chen et S.F. Yin | Bulbs | [1] |
| 36 *Ent*-kauran-17-acetoxy-16β-ol | *F. anhuiensis* S.C. Chen et S.F. Yin | Bulbs | [1] |
| 37 *Ent*-kauran-3β-acetoxy-16β,17-diol | *F. anhuiensis* S.C. Chen et S.F. Yin | Bulbs | [1] |
| 38 *Ent*-kauran-16α-methoxy-17-ol | *F. anhuiensis* S.C. Chen et S.F. Yin | Bulbs | [1] |
| 39 Fritillarinol A | *F. thunbergii* Miq. | Bulbs | [97] |
| 40 Fritillarinol B | *F. thunbergii* Miq. | Bulbs | [97] |
| 41 16α,17-epoxy-ent-kaurane | *F. thunbergii* Miq. | Bulbs | [97] |
| 42 16β,17-dihydroxyl-ent-kaurane | *F. thunbergii* Miq. | Bulbs | [97] |
| 43 16β-methoxy-17-hydroxyl-ent-kaurane | *F. thunbergii* Miq. | Bulbs | [97] |
| 44 (−)-ent-kaur-16-ene | *F. thunbergii* Miq. | Bulbs | [97] |
| 45 Isopimara-7,15-dien | *F. thunbergii* Miq. | Bulbs | [97] |
| 46 *Ent*-kauran-15-en-3α,17-diol | *F. monanth* Migo | Bulbs | [12] |
| 47 Fritillaziebinol (ent-kauran-16β-hydroxy-chloride) | *F. monanth* Migo | Bulbs | [12] |
|  | *F. ebeiensis* var*. purpurea* G.D. Yu et P. Li | Bulbs | [98] |
| 48 *Ent*-kauran-3α,16α,17-triol | *F. monanth* Migo | Bulbs | [12] |
| 49 *Ent*-16,17-epoxy-kauran-3α-ol | *F. monanth* Migo | Bulbs | [12] |

Table S5 Distribution information of steroidal saponins in *Fritillaria* species

| Name | Species | References |
| --- | --- | --- |
| 1 Pallidifloside A | *F. pallidiflora* Schrenk | [99] |
| 2 Pallidifloside B | *F. pallidiflora* Schrenk | [99] |
| 3 Pallidifloside C | *F. pallidiflora* Schrenk | [99] |
| 4 Pallidifloside D | *F. pallidiflora* Schrenk | [100] |
| 5 Pallidifloside E | *F. pallidiflora* Schrenk | [100] |
| 6 Spongipregnoloside A | *F. pallidiflora* Schrenk | [100] |
| 7 Smilaxchinoside C | *F. pallidiflora* Schrenk | [100] |
| 8 Pallidifloside G | *F. pallidiflora* Schrenk | [100] |
| 9 Pallidifloside H | *F. pallidiflora* Schrenk | [100] |
| 10 Pallidifloside I | *F. pallidiflora* Schrenk | [100] |
| 11 Timosaponin H1 | *F. pallidiflora* Schrenk | [100] |
| 12 Protobioside | *F. pallidiflora* Schrenk | [100] |
| 13 Polygonatoside B3 | *F. pallidiflora* Schrenk | [100] |
| 14 Polyphyllin V | *F. pallidiflora* Schrenk | [100] |
| 15 Deltonin | *F. pallidiflora* Schrenk | [100] |
| 16 Parispseudoside B | *F. pallidiflora* Schrenk | [101] |
| 17 (22S,25S)-26-*O*-*β*-D-glucopyranosyl-22,25-epoxyfurost-5-en-3*β*,26-diol-3-*O*-[α-L-rhamnopyranosyl(1→2)]-β-D-glucopyranoside | *F. pallidiflora* Schrenk | [101] |
| 18 (25R)-26-[*β*-D-glucopyranosyl]oxy]-3*β*-[(*O*-α-L-rhamnopyranosyl-(1→2)-*β*-D-glucopyranosyl)oxy]-cholesta-5,17-diene-16,22-dione | *F. pallidiflora* Schrenk | [101] |
| 19 26-*O*-*β*-D-glucopyranosyl-3,26-dihydroxy-(25R)-5*β*-furost-12-on-20(22)-ene-3-*O*-α-L-rhamnopyranosyl-(1→2)-*β*-D-glucopyranoside | *F. pallidiflora* Schrenk | [101] |
| 20 Aspidistrin | *F. pallidiflora* Schrenk | [101] |
| 21 (25R)-5*β*-spirostan-3*β*-yl *O*-β-D-glucopyranosyl-(1→4)-*O*-[α-L-rhamnopyranosyl-(1→2)]-*β*-D-glucopyranoside | *F. meleagris* L. | [10] |
| 22 (25R)-17α-hydroxyspirost-5-en-3*β*-yl *O*-α-L-rhamnopyranosyl-(1→2)-*β*-D-xylopyranoside | *F. meleagris* L. | [10] |
| 23 (25R)-17α-hydroxy-spirost-5-en-3*β*-yl *O*-α-L-rhamnopyranosyl-(1→2)-*β*-D-glucopyranoside | *F. meleagris* L. | [10] |
| 24 (25R)-17α-hydroxyspirost-5-en-3*β*-yl *O*-β-D-glucopyranosyl-(1→4)-*O*-[α-L-rhamnopyranosyl-(1→2)]-*β*-D-glucopyranoside | *F. meleagris* L. | [10] |
| 25 (25R,26R)-26-methoxyspirost-5-en-3*β*-yl *O*-β-D-glucopyranosyl-(1→4)-*O*-[α-L-rhamnopyranosyl-(1→2)]-*β*-D-glucopyranoside | *F. meleagris* L. | [10] |
| 26 (25R)-3*β*-[(*O*-α-L-rhamnopyranosyl-(1→2)-*β*-D-glucopyranosyl)oxy]-5α-spirostan-6-one | *F. meleagris* L. | [10] |
| 27 (25R)-26-[(*β*-D-glucopyranosyl)oxy]-22α-hydroxy-5*β*-furostan-3*β*-yl *O*-*β*-D-glucopyranosyl-(1→4)-*O*-[α-L-rhamnopyranosyl-(1→2)]-*β*-D-glucopyranoside | *F. meleagris* L. | [10] |
| 28 (25R)-26-[(β-D-glucopyranosyl)oxy]-17α,22α-dihydroxyfurost-5-en-3*β*-yl-*β*-D-glucopyranosyl-(1→4)-*O*-[α-L-rhamnopyranosyl-(1→2)]-*β*-D-glucopyranoside | *F. meleagris* L. | [10] |
| 29 (25R)-26-[(β-D-glucopyranosyl)oxy]-22α-hydroxyfurost-5-en-3*β*-yl *O*-*β*-D-glucopyranosyl-(1→4)-*O*-[α-L-rhamnopyranosyl-(1→2)]-*β*-D-xylopyranoside | *F. meleagris* L. | [10] |
| 30 (25R)-26-[(β-D-glucopyranosyl)oxy]-17α,22α-dihydroxyfurost-5-en-*β*-yl *O*-*β*-D-glucopyranosyl-(1→4)-*O*-[α-L-rhamnopyranosyl-(1→2)]-*β*-D-xylopyranoside | *F. meleagris* L. | [10] |
| 31 (25R)-26-[(*β*-D-glucopyranosyl)oxy]-17α,22α-dihydroxyfurost-5-en-3*β*-yl *O*-α-L-rhamnopyranosyl-(1→2)-*β*-D-glucopyranoside | *F. meleagris* L. | [10] |
| 32 (22S,25S)-spirosol-5-en-3β-yl *O*-β-D-glucopyranosyl-(1→4)-O-[α-L-rhamnopyranosyl-(1→2)]-*β*-D-glucopyranoside | *F. meleagris* L. | [10] |
| 33 (22R,25R)-spirosol-5-en-3*β*-yl *O*-α-L-rhamnopyranosyl-(1→2)-*β*-D-glucopyranoside | *F. meleagris* L. | [10] |
| 34 (22S,25S)-spirosol-5-en-3*β*-yl *O*-α-L-rhamnopyranosyl-(1→2)-*β*-D-glucopyranoside | *F. meleagris* L. | [10] |
| 35 (25R)-26-[(*β*-D-glucopyranosyl)oxy]-5*β*-furost-20(22)-en-3*β*-yl *O*-*β*-D-glucopyranosyl-(1→4)-*O*-[α-L-rhamnopyranosyl-(1→2)]-*β*-D-glucopyranoside | *F. meleagris* L. | [10] |
| 36 (25R)-3β-[(*O*-*β*-D-glucopyranosyl-(1→6)-*β*-D-glucopyranosyl)oxy]-26-[(*β*-D-glucopyranosyl)oxy]-5α-cholestane-6,22-dione | *F. meleagris* L. | [10] |
| 37 (25R)-3β-[(*β*-D-glucopyranosyl)oxy]-26-[(*β*-D-glucopyranosyl)oxy]-5α-cholestane-6,22-dione | *F. meleagris* L. | [10] |
| 38 (20R,22R)-22-[(*β*-D-glucopyranosyl)oxy]-3*β*,14α,20-trihydoxy-5α-cholestan-6-one | *F. meleagris* L. | [10] |
| 39 (25R)-△^5(6)^-isospirost-17α,3*β*-diol-3-*O*-*β*-D-glucopyranosyl-(1→3)-[α-L-rhamnopyranosyl-(1→2)-*β*-D-glucopyranoside | *F. pallidiflora* Schrenk | [102] |

Table S6 Distribution information of phenylpropanoids in *Fritillaria* species

| Name | Species | Botanical parts | References |
| --- | --- | --- | --- |
| 1 Syringaresinol | *F. thunbergii* Miq. | Aerial parts | [68] |
|  | *F. pallidiflora* Schrenk | Bulbs | [103] |
| 2 Clemaphenol A | *F.* *pallidiflora* Schrenk | Flowers | [104] |
| 3 Pinoresinol | *F. pallidiflora* Schrenk | Bulbs | [103] |
| 4 Murrayone | *F. pallidiflora* Schrenk | Bulbs | [57] |
| 5 2′-methoxyseselin | *F. pallidiflora* Schrenk | Bulbs | [81] |
| 6 Zhebeiresinol | *F. thunbergii* Miq. | Aerial parts | [105] |
| 7 Isorhamnetin | *F. thunbergii* Miq. | Flowers | [106] |
| 8 Kaempferol-3-O-α-L-rhamnoside | *F. thunbergii* Miq. | Flowers | [106] |
| 9 Kaempferol-3-O-α-L-glucoside | *F. thunbergii* Miq. | Flowers | [106] |
| 10 Kaempferitrin | *F. thunbergii* Miq. | Flowers | [106] |
| 11 Diosmetin | *F. pallidiflora* Schrenk | Bulbs | [57] |
| 12 Dihydroflavonol | *F. pallidiflora* Schrenk | Flowers | [104] |
| 13 Quercetin | *F. pallidiflora* Schrenk | Flowers | [104] |

Table S7 Distribution information of fatty acids in *Fritillaria* species

| Name | Species | Botanical parts | References |
| --- | --- | --- | --- |
| palmitic acid | *F. hupehensis* Hsiao et K.C. Hsia | Bulbs | [93] |
|  | *F. michailovskyi* Fomin | Bulbs | [11] |
|  | *F. cirrhosa* D. Don | Bulbs | [107] |
| lignoceric acid | *F. hupehensis* Hsiao et K.C. Hsia | bulbs | [93] |
| azelaic acid | *F. hupehensis* Hsiao et K.C. Hsia | bulbs | [93] |
| stearic acid | *F. michailovskyi* Fomin | Bulbs | [11] |
| laurostearic acid | *F. pallidiflora* Schrenk | Bulbs | [102] |
| linoleic acid | *F. walujewii* Regel | Bulbs | [22] |

Table S8 Distribution information of steride**s** in *Fritillaria* species

| Name | Species | Botanical parts | References |
| --- | --- | --- | --- |
| 1 β-sitosterol | *F. thunbergii* Miq. | Bulbs and aerial parts | [62,68] |
|  | *F. pallidiflora* Schrenk | Flowers | [104] |
|  | *F. anhuiensis* S.C. Chen et S.F. Yin | Bulbs | [1] |
|  | *F. unibracteata* Hsiao et K. C. Hsia | Bulbs | [108] |
|  | *F.* *michailovskyi* Fomin | Bulbs | [11] |
|  | *F. hupehensis* Hsiao et K.C. Hsia | Bulbs | [91] |
|  | *F. thunbergii* var. *chekiangensis* | Bulbs | [19] |
|  | *F. monantha* Migo | Bulbs | [33] |
|  | *F. walujewii* Regel | Bulbs | [22] |
| 2 Daucosterol (β-sitosterol-glucoside) | *F. anhuiensis* S.C. Chen et S.F. Yin | bulbs | [1,11] |
|  | *F.* *michailovskyi* Fomin | Bulbs | [1,11] |
|  | *F. thunbergii* Miq. | Bulbs | [62] |
|  | *F. thunbergii* var. *chekiangensis* | Bulbs | [19] |
|  | *F. pallidiflora* Schrenk | Bulbs | [102] |
|  | *F. walujewii* Regel | Bulbs | [22] |
| 3 7-ketositosterol | *F. unibracteata* Hsiao et K. C. Hsia | bulbs | [108] |

Table S9 Distribution information of other components in *Fritillaria* species

| Name | Species | Botanical parts | References |
| --- | --- | --- | --- |
| **1** L-Pyroglutamic acid | *F. pallidiflora* Schrenk | Bulbs | [104] |
| **2** Cyclo-(Leu–Val) | *F. anhuiensis* S.C. Chen et S.F. Yin | Bulbs | [1] |
| **3** Cyclo (*L*-Pro-*L*-Ala) | *F. pallidiflora* Schrenk | Bulbs | [103] |
| **4** Cyclo-(Phe-Val) | *F. pallidiflora* Schrenk | Bulbs | [103] |
| **5** Choline | *F. walujewii* Regel | Bulbs | [22] |
| **6** Uridine | *F. pallidiflora* Schrenk | Bulbs | [101] |
|  | *F. puqiensis* G.D. Yu et G.Y. Chen | Bulbs | [109] |
|  | *F. walujewii* Regel | Bulbs | [22] |
| **7** Uracil | *F. pallidiflora* Schrenk | Bulbs | [101] |
|  | *F. puqiensis* G.D. Yu et G.Y. Chen | Bulbs | [109] |
| **8** Adenosine | *F. pallidiflora* Schrenk | Bulbs | [106] |
|  | *F. michailovskyi* Fomin | Bulbs | [11] |
|  | *F. yuminensis* X.Z. Duan | Bulbs | [78] |
|  | *F. ussuriensis* Maxim | Stems and leaves | [110] |
|  | *F. cirrhosa* D. Don | Bulbs | [79] |
|  | *F. puqiensis* G.D. Yu et G.Y. Chen | Bulbs | [109] |
|  | *F. walujewii* Regel | Bulbs | [22] |
| **9** Thymidine | *F. cirrhosa* D. Don | Bulbs | [79] |
| **10** Thymine | *F. puqiensis* G.D. Yu et G.Y. Chen | Bulbs | [109] |
| **11** Adenine | *F. puqiensis* G.D. Yu et G.Y. Chen | Bulbs | [109] |
| **12** Guanosine | *F. michailovskyi* Fomin | Bulbs | [11] |
| **13** 1-Heptadecanol | *F. thunbergii* Miq. | Flower | [106] |
| **14** 3,7,11,15-tetramethylhexadeca-2E,6E,10E,14-tetraen-1-ol | *F. anhuiensis* S.C. Chen et S.F. Yin | Bulbs | [1] |
| **15** 1-hexadecanol | *F. cirrhosa* D. Don | Bulbs | [107] |
| **16** 1-octadecene | *F. cirrhosa* D. Don | Bulbs | [107] |
| **17** 1-eicosanol | *F. cirrhosa* D. Don | Bulbs | [107] |
| **18** Glycerol | *F. pallidiflora* Schrenk | Bulbs | [104] |
| **19** 3-octadecen-1-ol | *F. michailovskyi* Fomin | Bulbs | [11] |
| **20** Galacitiol | *F. ussuriensis* Maxim | Bulbs | [110] |
| **21** (Z)-2-(9-octadecenyloxy)-ethanol | *F. cirrhosa* D. Don | Bulbs | [107] |
| **22** 1-O-β-D-glucopyranosyl-(2S,3R,4E,8Z)-2-[(2-hydroxyoctadecanoyl) amido]-4,8-octadecadiene-1,3-diol | *F. pallidiflora* Schrenk | Bulbs | [57] |
| **23** Monoheptadecanoin | *F. thunbergii* Miq. | Flower | [106] |
| **24** palmitic acid glyceryl ester | *F. anhuiensis* S.C. Chen et S.F. Yin | Bulbs | [1] |
| **25** 9-Octadecynoic acid methyl ester | *F. cirrhosa* D. Don | Bulbs | [107] |
| **26** Z-2-Tetradecen-1-ol acetate | *F. cirrhosa* D. Don | Bulbs | [107] |
| **27** bis-(2-Ethylhexyl) phthalate | *F. cirrhosa* D. Don | Bulbs | [107] |
| **28** 9-octadecenoic acid methyl ester | *F. pallidiflora* Schrenk | Bulbs | [103] |
| **29** Methyl ferulate | *F. pallidiflora* Schrenk | Bulbs | [103] |
| **30** 1-*O*-feruloylglycerol | *F. pallidiflora* Schrenk | Bulbs | [103] |
| **31** 2,3-*O*-diferuloylglycerol | *F. pallidiflora* Schrenk | Bulbs | [103] |
| **32** 1,3-*O*-diferuloylglycerol | *F. pallidiflora* Schrenk | Bulbs | [103] |
| **33** *bis* (diethylene glycol) phthalate | *F. pallidiflora* Schrenk | Bulbs | [103] |
| **34** Lauric acid 1-monoglyceride | *F. pallidiflora* Schrenk | Bulbs | [102] |
| **35** Methyl octadecanoate | *F. unibracteata* Hsiao et K. C. Hsia | Bulbs | [108] |
| **36** 1,3,3-trimethyl-1-phthalanol | *F. cirrhosa* D. Don | Bulbs | [107] |
| **37** α-Monopalmitin | *F. pallidiflora* Schrenk | Bulbs | [57] |
| **38** *cis*-cinnamic acid | *F. pallidiflora* Schrenk | Bulbs | [103] |
| **39** *trans*-isoferulic acid | *F. pallidiflora* Schrenk | Bulbs | [103] |
| **40** *Trans*-cinnamic acid | *F. pallidiflora* Schrenk | Bulbs | [102] |
| **41** 4-(*β*-D-glucopyranosyloxy) benzoic acid | *F. pallidiflora* Schrenk | Bulbs | [101] |
| **42** 5,7-dihydroxy-2-(4-hydroxy-3-methoxyphenyl)-3-methoxy-4H-chromen-4-one | *F. thunbergii* Miq. | Flower | [106] |
| **43** 4,7-dimethyl-benzofuran | *F. cirrhosa* D. Don | Bulbs | [107] |
| **44** 3-methyl-4-phenyl-3-buten-2-one | *F. cirrhosa* D. Don | Bulbs | [107] |
| **45** Acetovanillone | *F. pallidiflora* Schrenk | Bulbs | [103] |
| **46** 1-dodecene | *F. cirrhosa* D. Don | Bulbs | [107] |
| **47** 4-octadecene | *F. michailovskyi* Fomin | Bulbs | [11] |
| **48** Pentatriacontane | *F. cirrhosa* D. Don | Bulbs | [107] |
| **49** gastrodin | *F. pallidiflora* Schrenk | Bulbs | [101] |
| **50** Icariside D2 | *F. pallidiflora* Schrenk | Bulbs | [101] |
| **51** Hexadecyl-oxirane | *F. cirrhosa* D. Don | Bulbs | [107] |
| **52** 3-methoxy-4-(palmitoyloxy)benzaldehyde | *F. unibracteata* Hsiao et K. C. Hsia | Bulbs | [108] |
| **53** 1,4-diphenylbutane | *F. pallidiflora* Schrenk | Bulbs | [103] |
| **54** 4-hydroxy-3-methoxy-benzaldehyde | *F. pallidiflora* Schrenk | Bulbs | [103] |
| **55** 9-β-D-erythrofuranosyladenine | *F. michailovskyi* Fomin | Bulbs | [11] |
| **56** Succinic acid | *F. ussuriensis* Maxim | Bulbs | [110] |
| **57** 1-O-β-D-glucopyranosyl-(2S,3S,4R,8Z)-2-[(2′R)-(2′-hydroxydocosanoyl) amido]-1,3,4-octadecanetriol-8-ene | *F. pallidiflora* Schrenk | Bulbs | [81] |
| **58** Triethylamine hydrochloride | *F. walujewii* Regel | Bulbs | [22] |
| **59** 2, 5 dimethoxy-1, 4-benzoquinoe | *F. thunbergii* Miq. | Stems and leaves | [68] |

Table S10 Pharmacological activities of *Fritillaria* species

| **Biological activities** | **Extract & Chemical components** | **Simple description** | ***In vivo or in vitro*** | **References** |
| --- | --- | --- | --- | --- |
| **Anti-tussive** | puqienine A, puqienine B, N-demethylpuqietinone, puqietinonoside, puqietinone | Prolonged the latent period and reduced the cough times | *In vivo* | [15] |
|  | imperialine, chuanbeinone, verticinone, and verticine | Inhibited cough frequency and increased latent period of cough in mice induced by ammonia | *In vivo* | [111] |
|  | imperialine, verticine, verticinone, ebeiedine, and puqietinone | The mechanism was related to the competitive antagonism in muscarinic pathway and the inhibition of influx of calcium ions | *In vivo* | [112] |
|  | imperialine, imperialine-β-N-oxide, isoverticine, and isoverticine-β-N-oxide | Inhibited cough frequency and increased latent period of cough in mice induced by ammonia | *In vivo* | [113] |
|  | alcohol extracts of *F. cirrhosa* and *F. pallidiflora* | Inhibited cough frequency and increased latent period of cough in mice induced by ammonia | *In vivo* | [114] |
|  | crude alkaloid and water extracts *F. thunbergii*, *F. cirrhosa*, *F. hupehensis* and *F. pallidiflora* | Showed a dose-dependent tracheobronchial relaxation with different potencies correlated with their tracheobronchial relaxation effects | *In vivo* | [115] |
|  | ebeinone | Exhibited a higher affinity for muscarinic M_2_ receptors than for M_3_ receptors in the guinea-pig | *In vivo* | [116] |
|  | imperialine and sinpeinine A | Selective inhibitory effects on muscarinic M_2_ receptors | *In vitro* | [117] |
|  | 3β-acetylimperialine the derivative of imperialine | Selective muscarinic M_3_ receptors antagonism | *In vitro* | [117] |
|  | verticine, verticinone, imperialine, imperialine-3β-D-glucoside and puqietinone | Elevated the cAMP concentration in the HEK cells transfected with muscarinic M_2_ receptor plasmid | *In vitro* | [118] |
| **Expectorant** | imperialine, verticinone and verticine | Enhanced mice’s tracheal phenol red output in expectorant assessment | *In vivo* | [111] |
|  | imperialine, imperialine-β-N-oxide, isoverticine and isoverticine-β-N-oxide | Enhanced mice’s tracheal phenol red output in expectorant assessment | *In vivo* | [4] |
|  | alcohol extracts of *F. cirrhosa* and *F. pallidiflora* | Enhanced mice’s tracheal phenol red output in expectorant assessment | *In vivo* | [5] |
| **Antiasthmatic** | water extract of *F. cirrhosa* | Inhibitory effects on airway inflammation by suppression of Th2 cytokines (IL-4, IL-5 and IL-13), IgE, histamine production, reduction eosinophilic accumulation and increase of interferon-γ production | *In vivo* | [119] |
|  | yibeinones B-D and imperialine | Showed an apparent concentration-dependent relaxation of isolated tracheal preparation | *In vivo* | [75] |
| **Other respiratory diseases** | imperialine | Mitigated pulmonary functional and structural impairment and suppressed inflammatory response in a COPD-like rat model by mediating expression of related cytokines in lung tissues of the COPD-like rats, such as IL-1β, IL-6, IL-8, TNF-α, NF-κB, TGF-β1, MMP-9, and TIMP-1. | *In vivo* | [120] |
|  | peiminine | In vivo, peiminine reduced the Wet-to-Dry ratio and the myeloperoxidase activity and inhibited IL-6. In vitro, peiminine significantly inhibited LPS-induced IL-8 production in A549 lung epithelial cells via inhibition of NF-κB and AKT and PI_3_K phosphorylation | *In vivo* & *in vitro* | [119] |
|  | verticine, ebeiedine and suchengbeisine | Inhibited the gene expression and production of MUC5AC mucin, by directly acting on airway epithelial cells | *In vitro* | [121] |
| **Antineoplastic** | verticinone | Inhibited the growth of HL-60 cells by inducing these cells to differentiate toward granulocytes and minimized the effective dose of all-trans retinoic acid | *In vitro* | [122] |
|  | verticinone | Induced apoptosis through a caspase pathway mediated by mitochondrial damage in immortalized keratinocytes and oral cancer cells and G_0_G_1_ cell cycle arrest | *In vitro* | [123] |
|  | 5β-spirostanol glycoside | Induced apoptotic cell death in HL-60 cells through different mechanisms of action | *In vitro* | [10] |
|  | water extract of *F. cirrhosa* | NF-κB activation | *In vitro* | [124] |
|  | chloroform extract and the purified total alkaloids of *F. ussuriensis* | Exhibited stronger cytotoxic activity, induced G_0_/G_1_ phase arrest and increased apoptosis | *In vitro* & *in vivo* | [125] |
|  | water extract of *F. cirrhosa* | Block endometrial cancer growth by downregulating TGF-β/SMAD signaling pathway | *In vitro* | [126] |
|  | chloroform extracts**-**total alkaloid-chuanbeinone, imperialine-β-N-oxide, isoverticine and isoverticine-β-N-oxide of *F. pallidiflora* | Exhibited significant antitumor activity *in vivo*, while notably inhibited tumor angiogenesis and inducing apoptosis characterized by an increased expression of caspase-3 | *In vitro* & *in vivo* | [120] |
|  | isopimara-7,15-Dien-19-oic acid | Induce cellular stress in HeLa cells and activate several anti- and prosurvival pathway. | *In vitro* | [127] |
|  | peiminine | Induced autophagic cell death via activating autophagy-related signaling pathway AMPK-mTOR-ULK by promoting SQSTM1(P62) | *In vivo* | [128] |
|  | peiminine | Inhibited glioblastoma *in vitro* and *in vivo* via arresting the cell cycle and blocking autophagic flux | *In vitro* & *in vivo* | [129] |
|  | Imperialine | Suppressed bothnon-small cell lung cancer (NSCLC) tumor and associated inflammation through an inflammation-cancer feedback loop | *In vitro* & *in vivo* | [130] |
|  | peimine | Inhibited the growth and motility of prostate cancer cells and induced apoptosis by disruption of intracellular calcium homeostasis through Ca^2+^/CaMKII/JNK pathway | *In vitro* & *in vivo* | [131] |
| **anti-inflammatory** | imperialine, chuanbeinone | Inhibited the development of ear edema in a dose-dependent manner in anti-inflammatory assessment | *In vivo* | [111] |
|  | imperialine, imperialine-β-N-oxide, isoverticine, and isoverticine-β-N-oxide | Inhibited the development of ear edema in anti-inflammatory evaluation assay | *In vivo* | [113] |
|  | alcohol extract of *F. cirrhosa & F. pallidiflora* | Inhibited the development of ear edema in anti-inflammatory evaluation assay | *In vivo* | [114] |
|  | 12,15-sulfonyl-8(17),13-labdadien-19-oic acid | Attenuated nitric oxide production of a macrophage cell line of Raw 264.7 cells stimulated with IFN-γ | *In vitro* | [92] |
|  | verticinone and imperialine | Inhibition of the NF-κB activation signaling pathway | *In vitro* | [132] |
|  | puqiedine, zhepeiresinol, 2-monopalmitin, N-demethylpuqietinone, and isoverticine | Decreased the expression of NF-κB | *In vitro* | [133] |
|  | total alkaloid fraction of *F. cirrhosa* | Inhibited acetic acid-induced capillary permeability accentuation, carrageenan-induced paw edema, cotton pellet-induced granuloma formation, suppressed inflammatory cells recruitment and cytokine production in the bronchoalveolar lavage fluid from LPS-induced ALI mice, and attenuated pathological changes in the lung tissues of ALI mice. | *In vivo* | [134] |
|  | crude polysaccharide extracted *F. hupenesis* | Inhibited mouse ear swelling induced by xylene and inhibited toe swelling induced by egg white and had good anti-inflammatory activity against LPS-induced inflammation, reducing the expression of inflammatory cytokines | *In vivo* | [135] |
|  | peimine | Suppressed IL-1β-induced inflammation in mouse chondrocytes by inhibiting the MAPK pathway | *In vitro* | [136] |
|  | peimine | Inhibited IL-1β induced inflammatory response in mouse articular chondrocytes and ameliorated murine osteoarthritis, inhibited the AKT phosphorylation, NF-κB and activated Nrf2/HO-1 signaling pathways both *in vitro* and *in vivo*. | *In vitro* & *in vivo* | [137] |
|  | imperialine, verticinone, verticine, peimisine and delavine | Down-regulated the level of inflammatory mediators via mediation of MAPK phosphorylation in LPS-induced RAW264.7 macrophages | *In vitro* | [138] |
| **Antihypertensive** | water extract, ethylacetate and butanol extracts of *F. ussuriensis* | Via the inhibition of angiotensin converting enzyme activity and direct release of NO/cGMP in the vascular tissue | *In vivo* | [139] |
|  | verticinone, verticine, peimisine | Inhibited angiotensin I converting enzyme activity in a dose-dependent manner, displaying 50% inhibitory concentration values | *In vitro* | [140] |
|  | water extract of *F. ussuriensis* | Prevented the increase of systolic blood pressure in the L-NAME-induced hypertension that may have been caused by enhanced generation of vascular NO and amelioration of renal functions | *In vivo* | [141] |
|  | puqienine E, puqienine B and puqienine A | Exhibited inhibitory activity against angiotensin converting enzyme | *In vitro* | [72] |
| **Anti-cholinesterase (cholinomimetic)** | impericine, forticine, delavine, persicanidine A and imperialine | Showed anti-acetylcholinesterase and anti-butyrylcholinesterase inhibitory activity | *In vitro* | [54] |
|  | N-demethylpuqietinone, hupeheninoside, ebeiedinone, yibeinoside A and chuanbeinone | Had anti-red blood cell acetylcholinesterase and anti-plasma butyrylcholinesterase activities | *In vitro* | [142] |
|  | aqueous methanol extracts, solanidine of *F. unibracteata* | Showed the antimuscarinic effects | *In vitro* | [133] |
|  | dichloromethane fraction of *F. michailovskyi* | Showed a positive butyrylcholinesterase inhibitory activity | *In vitro* | [11] |
| **Antibacterial and antiviral** | β-sitosterol-3-O-glucopyranoside | A potent inhibitor of sortase, a bacterial surface protein anchoring transpeptidase against *Bacillus subtilis, Staphylococcus aureus, and Micrococcus leuteus* | *In vitro* | [143] |
|  | ethanol and aqueous extracts *F. thunbergii* | Remarkably inhibitory against all the six Helicobacter pylori strains (MICs: ～ 60 μg/ml) | *In vitro* | [144] |
|  | water extracts *F. thunbergii* | Exerted antiviral effects against influenza *H1N1 viru*s without inducing toxicity *in vitro*, *in ovo* or *in vivo* | *In vitro*, *in ovo*, and *in vivo* | [145] |
| **Antioxidant** | the ethanol extracts and the non-alkaloid fractions, the crude saponin extract and the crude flavonoids extract of *F. ussuriensis* | The antioxidant activity of different extracts decreased in the order: crude flavonoids extract > crude saponin extract > ethanol extracts | *In vitro* | [146] |
|  | Supercritical fluid extraction, peimisine, peimine and peiminine of *F. thunbergii* | The antioxidant capacity of extracts displayed EC_50_, 1,1-diphenyl-2-picrylhydrazylvalue of 5.5 mg/mL, EC_50_, 2, 2’-azino-bis(3-ethylbenzothiazoline-6-sulfonic acid) value of 0.3 mg/ml and ferric reducing capacity value of 118.2 mg ascorbic acid equivalent/100 g. | *In vitro* | [147] |
|  | an acidic water-soluble heteropolysaccharide (FWPS1-1) (average molecular weight: ~7.44 kDa) *F. unibracteata* | Exhibited weak 1,1-diphenyl-2-picrylhydrazyl radical scavenging activity and low ferric reducing antioxidant power but high 2,2-azino-bis(3-ethylbenzthiazoline-6-sulfonic acid) radical scavenging activity, good Fe(II)-chelating ability and remarkable DNA damage protective activity | *In vitro* | [148] |
|  | the acidic fraction FPSP-H2-1 *F. pallidiflora* | Had a strong antioxidant effect against 1,1-diphenyl-2-picrylhydrazyl, hydroxyl and 2, 2’-azino-bis(3-ethylbenzothiazoline-6-sulfonic acid) free radicals | *In vitro* | [149] |
|  | verticinone, verticine, imperialine-3-β-D-glucoside, delavine and peimisine, imperialine | Six isosteroidal alkaloids reduced reactive oxygen species production, elevated glutathione level and promoted heme oxygenase expression, which is in association with induction of NF-E2-related factor 2 (Nrf2) nuclear translocation and up-regulation of Nrf2 expression. Among these alkaloids, verticinone, verticine, imperialine-3-β-D-glucoside, delavine and peimisine exhibited more potent effect against cigarette smoke extract-induced oxidative stress than that of imperialine. | *In vitro* | [150] |
| **Antinociceptive** | verticinone | Exerted a good antinociceptive effect on inflammatory pain and cancer-related neuropathic pain probably through both peripheral and central mechanisms | *In vivo* | [151] |
|  | peimine | Blocked the Nav1.7 ion channel but also preferably inhibited the Kv1.3 ion channel | *In vitro* | [152] |
| **Anti-allergy** | ethanol extract of *F. ussuriensis* | Significantly inhibited the passive cutaneous anaphylaxis reaction and the release of histamine from rat peritoneal mast cells in a concentration-dependent manner, which was associated with the inhibition of IL-6, IL-8, TNF-α and phosphorylation of all three MAPKs | *In vitro & in vivo* | [153] |
|  | peiminine | *In vitro* inhibited the production of the pro-inflammatory cytokine, such as IL-6, IL-8, TNF-a and IL-1β. It was shown to have inhibitory effects on MAPKs phosphorylation and NF-κB expression in human mast cells (HMC)-1  *In vivo* demonstrated inhibitory effects on IgE-dependent anaphylaxis | *In vitro* & *in vivo* | [154] |
| **Neuroprotective** | ent-3β-butanoyloxykaur-15-en-17-ol; ent-kaur-15-en-17-ol; ent-kaur-15-en-3β,17-diol; ent-3β-acetoxykaur-15-en-17-ol; ent-kauran-16β,17-diol | Showed neuroprotective effects against MPP^+^-induced neuronal cell death in human dopaminergic neuroblastoma SH-SY5Y cells | *In vitro* | [155] |
|  | 6α,7β-dihydroxy-labda-8(17),12(E),14-triene and 6-oxo-2α-hydroxy-labda-7,12(E), 14-triene | Showed neuroprotective effects against MPP^+^-induced neuronal cell death in human dopaminergic neuroblastoma SH-SY5Y cells | *In vitro* | [3] |
|  | peimisine-3-O-β-D-glucopyranoside | Showed moderate protection effect on neurotoxicity of PC12 cell lines induced by rotenone. | *In vitro* | [73] |
|  | peiminine | Markedly attenuated behavioral dysfunction and inhibited the loss of dopaminergic neurons and microglial activation in the LPS-induced Parkinson’s disease rat model. In BV-2 cells, peiminine significantly decreased LPS-induced expression of the pro-inflammatory mediators TNF-α, IL-6 and IL-1β, COX-2 and iNOS by inhibiting the phosphorylation of ERK1/2, AKT and NF-κB p65 | *In vitro* & *in vivo* | [156] |
| **Anti-diabetic** | verticinone | Increased insulin secretion and glucose uptake and inhibition of carbohydrate-hydrolyzing enzymes | *In vitro* | [157] |

**References:**

1 Shou Q, Wohlmuth H, He X, Liu L, Shen Z. Chemical constituents from *Fritillaria anhuiensis*. Biochem. Syst. Ecol. 2012;45:16-19.

2 Guo X, Wu X, Ni J, Zhang L, Xue J, Wang X. Aqueous extract of bulbus *Fritillaria cirrhosa* induces cytokinesis failure by blocking furrow ingression in human colon epithelial NCM460 cells. Mutat Res Gen Tox En. 2020;850-851:503147.

3 Xu J, Liu C, Guo P, Guo Y, Jin D, Song X, et al. Neuroprotective labdane diterpenes from *Fritillaria ebeiensis*. Fitoterapia. 2011;82:772.

4 Wu J, Wen Y, Ruan H, Yao N, Zhao Q, Sun H, et al. Structural elucidations of two ent-kaurane dimers from bulbs of *Fritillaria ebeiensis* var. *purpurea*. J. Asian Nat. Prod. Res. 2000;2:213-218.

5 Kardan M, Yazdani Z, Morsaljahan Z, Ebrahimzadeh M, Rafiei A. Cytotoxic effect of methanolic extracts of *Fritillaria imperialis* bulbs and *Eryngium caucasicum* leaves on hepatoma and colon cancer cells. Asian Pac J Trop Biomed. 2019;9:353.

6 Akhtar MN, Atta-ur-Rahman, Choudhary MI, Sener B, Erdogan I, Tsuda Y. New class of steroidal alkaloids from *Fritillaria imperialis*. Phytochemistry. 2003;63:115-122.

7 Huang J, Lei C, Aisa HA, Yu M, Yili A, Hou A. Isosteroidal alkaloids from Fritillaria karelinii. Chinese J. Org. Chem. 2019;39:842-847.

8 Pi H, Ruan H, Zhang Y, Niu L, Wu J. Steroidal alkaloids from bulbs of *Fritillaria lichuanensis*. J. Asian Nat. Prod. Res. 2006;8:133-136.

9 Qian ZZ, Nohara T. Steroidal alkaloids of *Fritillaria maximowiczii*. Phytochemistry. 1995;40:979-981.

10 Matsuo Y, Shinoda D, Nakamaru A, Mimaki Y. Steroidal glycosides from the bulbs of *Fritillaria meleagris* and their cytotoxic activities. Steroids. 2013;78:670-682.

11 Wang Y, Aamer M, Aslay M, Sener B, Khan F, Wahab A, et al. A new steroidal alkaloid from *Fritillaria michailovskyi* Fomin. Nat. Prod. Res. 2020;1-6.

12 Hongning L, Li F, Luo Y, Zhu W, Yan D, Huang X. Diterpenoids from bulbs of Fritillaria monanth. Acta Pharmaceutica Sinica. 2007;42:1152-1154.

13 Li Q, Wu Z, Zhang L, Shao L. Isolation and identification of alkaloids from Fritillaria ningguoensis S. C. Chen et S. F. Yin. Acta Pharmaceutica Sinica. 1988;23:415-421.

14 Wang D, Li Z, Zhang L, Atanasov A, Wang S. Characterization of the isosteroidal alkaloid chuanbeinone from bulbus of *Fritillaria pallidiflora* as novel antitumor agent *in vitro* and *in vivo*. Planta Med. 2016;82:195-204.

15 Jiang Y, Li H, Li P, Cai Z, Ye W. Steroidal alkaloids from the bulbs of *Fritillaria puqiensis*. J. Nat. Prod. 2005;68:264-267.

16 Khare CP. (2007). Indian Medicinal Plants *Fritillaria roylei* Hook. In. New York: Springer.

17 Huang S, Zhou X, Wen J, Wang C, Wang H, Shan L, et al. A novel steroidal alkaloid from *Fritillaria shuchengensis*. J Nat Med. 2013;67:647-651.

18 Hu C, Shang E, Lin W, Cai M. Studies on the chemical constituents of Fritillaria taipaiensis L. Acta Pharmaceutica Sinica. 1993;28:516-521.

19 Zhang J, Lao A, Chen Q, Xu R. Studies on the chemcial constituents of Dongbeimu (Fritillaria thunbergii var. chekiangensis) (I). Chinese Traditional and Herbal Drugs. 1993;24:341-342.

20 Kitamura Y, Kaneko K, Shiro M, Chen Y, Hsh H, Lee P, et al. Tortifoline, a novel (20S, 22R)-5α-cevanine alkaloid from *Fritillaria tortifolia*. Chem. Pharm. Bull. 1989;37:1514-1516.

21 Xu D, Wang S, Huang E, Xu M, Zhang Y, Wen X. Isolation and identification of pingpeimine B. Acta Pharmaceutica Sinica. 1988;23:902-905.

22 Shi L, Lu X, Meng L, Lv W, Liu Q, Liu Y. Chemical constituents from the bulbs of Fritillaria walujewii. Journal of Chinese Medicinal Materials. 2017;40:2098-2100.

23 Jian-fa Z, Zhu H, Aisa H, Chun L, Yili A, Ai-jun H. Studies on alkaloid constituents of Fritillaria yuminensis. China Journal of Chinese Materia Medica. 2019;44:495-499.

24 Xu M, Xu D, Huang E, Zheng W. Alkaloids research of Fritillaria ussuriensis Maxim flower. Bulletin of Chinese Materia Medica. 1988;13:32-33.

25 Xu D, Huang E, Xu M, Zheng W, Sun Z. Chemical components of stems and leaves in Fritillaria ussuriensis. Traditional Chinese Medicine Journal. 1986;11:40-41.

26 Xu D, Zhang B, Li H, Xu M. Isolation and identification of alkaloids from Fritillaria ussuriensis Maxim. Acta Pharmaceutica Sinica. 1982;17:355-359.

27 Yang Z, Duan D. A new alkaloid from *Fritillaria ussuriensis* Maxim. Fitoterapia. 2012;83:137-141.

28 Zhang J, Lao A, Huang H, Ma G, Xu R. Study on the chemical constituents of Fritillaria thunbergii Miq. III. isolation and identification of zhebeinone. Acta Pharmaceutica Sinica. 1992;27:472-475.

29 Cao X, Chen S, Li J, Xiao P, Chen S. Steroidal alkaloids from the bulbs of *Fritillaria delavayi* Franch. (Liliaceae). Biochem. Syst. Ecol. 2008;36:665-668.

30 Wu J. Research of chemical component of Fritillaria hupehensis Ⅰ alkaloid research. Chinese Traditional and Herbal Drugs. 1982;13:3-6.

31 Wu J, Li H, Zhu J. Research of chemical components of Fritillaria genus plants Ⅶ. isolation and authentication of alkaloid in Fritillaria ebeiensis. Chinese Traditional and Herbal Drugs. 1989;20:5-7.

32 Wu J, Pan X, Lou M, Wang X, Ling D. Research of chemical components of Fritillaria genus plants Ⅶ. isolation and authentication of alkaloid in Fritillaria ebeiensis var. purpuea. Acta Pharmaceutica Sinica. 1989;24:600-605.

33 Zhang Z, Fan C. Research of chemical components of Jiangxi Fritillaria monantha Migo (I). Chinese Traditional and Herbal Drugs. 1994;48.

34 Cao X, Li M, Li J, Xiao P, Chen S, Chen S. Alkaloid constituents of Fritillaria cirrhosa. Chinese Traditional and Herbal Drugs. 2009;40:15-17.

35 Li C, Li D, Li L, An M, Zhong S, Jiang H, et al. Study on alkaloid constituents from the bulbus of cultivated Fritillaria wabuensis. West China Journal of Pharmaceutical Sciences. 2019;34:463-467.

36 Wang S, Huang E, Wen X, Wu X, Xu D. Studies on the steroid alkaloids from stems and leaves of Fritillaria ussuriensis Maxim. China Journal of Chinese Materia Medica. 1991;16:554-555.

37 Zhang A, Wang H, Tang X, Zheng Y, Yi X, Yu K. Isolation and structure elucidation of alkaloids from the bulb of *Fritillaria wabuensis*. Planta Med. 1998;64:448-450.

38 Zhang Y, Yang X, Zhang P, Zhou X, Ruan H, Pi H, et al. Cytotoxic alkaloids from the bulbs of *Fritillaria hupehensis*. C&B. 2008;5:259-266.

39 Xu D, Arihara S, Shoji N, Yang X, Huang E, Li C. Isolation and identification of yibeinoside A. Acta Pharmaceutica Sinica. 1990;25:795-797.

40 Liu Q, Jia X, Ren Y, Muhatal, Liang X. Study on the constituents of Fritillaria walujewii. Acta Pharmaceutica Sinica. 1984;19:894-898.

41 Li H, Jiang Y, Li P. Chemistry, bioactivity and geographical diversity of steroidal alkaloids from the Liliaceae family. Nat. Prod. Rep. 2006;23:735.

42 Kaneko K, Katsuhara T, Mitsuhashi H, Chen Y, Hsu H, Shiro M. Isolation and structure elucidation of new alkaloids from *Fritillaria delavayi* Franch. Chem. Pharm. Bull. 1985;33:2614-2617.

43 Hu Z, Zong J, Yili A, Yu M, Aisa HA, Hou A. Isosteroidal alkaloids from the bulbs of *Fritillaria tortifolia*. Fitoterapia. 2018;131:112-118.

44 Feng R, Lin W, Cai M. Studies on the chemical constituents of *Fritillaria taipaiensis* L. Yao xue xue bao = Acta pharmaceutica Sinica. 1993;28:516-521.

45 Kaneko K, Katsuhara T, Mitsuhashi H, Chen Y, Hsu H, Shiro M. Chuanbeinone, a novel D/E cis-(22R,25S)-5α-cevanine alkaloid from Chinese herbal drug, chuan-bei-mu. Tetrahedron Lett. 1986;27:2387-2390.

46 Zhang J, Lao A, Ma G, Xu R. Studies on Chemical constituents of Fritillaria thunbergiss Miq. Acta Botanica Sieica. 1991;33:923-926.

47 Lin G, Ho Y, Li P, Li X. Puqiedinone, a novel 5α-cevanine alkaloid from the bulbs of *Fritillaria puqiensis*, an antitussive traditional Chinese medicine. J. Nat. Prod. 1995;58:1662-1667.

48 Xu D, Huang E, Wang S, Wen X, Wu X. Studies on the chemical constituents of Fritillaria palidiflora Schrenk. Acta Botanica Sinica. 1990;32:789-793.

49 Wang H, Zhang A, Tang X, Zheng Y, Yi X, Yu K. Isolation and Structure flucidation of alkaloids from the bulb of Fritillaria wabuensis S.T. Tang et S.C. Yueh. Journal of West China University of Medical Sciences. 1996;27:100-105.

50 Jiang RW, Ma SS, But PP, Dong H, Mak TC. Sipeimine, a steroidal alkaloid from *F*ritilla*ria roylei* Hooker. Acta Crystallogr C. 2001;57:170-171.

51 Xu D, He C, Wang S, Huang E, Xu M, Wen X. Structure of pingbeimine C. Acta Pharmaceutica Sinica. 1990;25:127-130.

52 Zhang P, Pi H, Zhang J, Ruan H, Zhang Y, Wu J. Alkaloid of stems and leaves in Fritillaria hupehensis. Chinese Traditional and Herbal Drugs. 2008;39:1294-1296.

53 Wu J, Wang Y, Ling D. Study on chemical constituent of hubeibeimu (Fritillaria hupehensis Hsiao et K.C. Hsia) V. isolation and identification of hupehensine. Acta Pharmaceutica Sinica. 1986;21:546-550.

54 Atta-Ur-Rahman, Akhtar MN, Choudhary MI, Tsuda Y, Sener B, Khalid A, et al. New steroidal alkaloids from *Fritillaria imperialis* and their cholinesterase inhibiting activities. Chem. Pharm. Bull. 2002;50:1013-1016.

55 Jiang Y, Li P, Li H, Yu H. New steroidal alkaloids from the bulbs of *Fritillaria puqiensis*. Steroids. 2006;71:843-848.

56 Chen Q, Zhu L, Xu Y, Fan J. A new steroidal alkaloid from the bulbs of Fritillaria wabuensia. Acta Pharmaceutica Sinica. 2004;39:348-350.

57 Xu W, Liu M, Chen D, Wang J. Chemical constituents from bulbs of *Fritillaria pallidiflora* Schrenk. Biochem. Syst. Ecol. 2014;57:198-202.

58 Zhang Y, Yang X, Zhou X, Ruan H, Pi H, Wu J, et al. Alkaloids from *Fritillaria hupehensis*. Chin. J. Chem. 2007;25:1728-1731.

59 Li H, Jiang Y, Li P, Ye W. Puqienine F, a novel veratramine alkaloid from the bulbs of *Fritillaria puqiensis*. Chem. Pharm. Bull. 2006;54:722-724.

60 Xu Y, Xu D, Huang E, Wu X, Jin X, Cui D, et al. Isolation and identification of yibeissine. Acta Pharmaceutica Sinica. 1992;27:121-124.

61 Liu Y, Feng Y, Lu X, Nie J, Li W, Wang L, et al. Isosteroidal alkaloids as potent dual-binding site inhibitors of both acetylcholinesterase and butyrylcholinesterase from the bulbs of *Fritillaria walujewii*. Eur. J. Med. Chem. 2017;137:280-291.

62 Zhang J, Lao A, Xu R. Studies on the chemical constituents of fresh bulbs of Fritillaria thunbergii Miq. China Journal of Chinese Materia Medica. 1993;18:354-355.

63 Mimakli Y, Sashida Y. Studies on the chemical constituents of the bulds of *Fritillaria camtschatcensis*. Chemical and Pharmaceutical Bulletin. 1990;38:1090-1092.

64 Yu S, Xiao P. Study on the chemical constituents of Unibract Fritillary (Fritillaria unibracteara) (I). Chinese Traditional and Herbal Drugs. 1990;21:2-6.

65 Liu H, Li F, Luo Y, Zhu W. Two novel isosteroid alkaloids from *Fritillaria monatha*. J. Asian Nat. Prod. Res. 2007;9:563-567.

66 Liu HN, Li F, Luo YM, Zhu WF. Steroidal alkaloids from the bulbs of *Fritillaria monatha*. Chinese Chem. Lett. 2008;19:544-546.

67 Shou QY, Tan Q, Wu Shen Z. Two 22S-solanidine-type steroidal alkaloids from *Fritillaria anhuiensis*. Fitoterapia. 2010;81:81-84.

68 Yan M, Jin X, Xu D. Studies on the chemical constituents of the stems and leaves of Thunberg Fritillary (Fritillaria thunbergii). Chinese Traditional and Herbal Drugs. 1994;25:344-346.

69 Kitajima J, Komori T, Kawasaki T, Schulten H. Basic steroid saponins from aerial parts of *Fritillaria thunbergii*. Phytochemistry. 1982;21:187-192.

70 Kaneko K, Tanaka M, Nakaoka U, Tanaka Y, Yoshida N, Mitsuhashi H. Camtschatcanidine, an alkaloid from *Fritillaria camtschatcensis*. Phytochemistry. 1981;20:327-329.

71 Xu D, Wang S, Huang E, Xu M. New steroidal saponin of stem and leaf of Fritillaria ussuriensis Maxim. Acta Botanica Sinica. 1989;31:285-288.

72 An J, Zhou J, Li H, Jiang Y, Li P. Puqienine E: An angiotensin converting enzyme inhibitory steroidal alkaloid from *Fritillaria puqiensis*. Fitoterapia. 2010;81:149-152.

73 Zhang Q, Zheng Z, Yu D. Steroidal alkaloids from the bulbs of *Fritillaria unibracteata*. J. Asian Nat. Prod. Res. 2011;13:1098-1103.

74 Liu H, Li F, Luo Y, Zhu W. Two new Isosteroid alkaloids from *Fritillaria monatha* Migo (Ⅲ). Chinese Chem. Lett. 2006;17:631-634.

75 Li Y, Yili A, Li J, Muhamat A, Aisa HA. New isosteroidal alkaloids with tracheal relaxant effect from the bulbs of *Fritillaria pallidiflora* Schrenk. Bioorg. Med. Chem. Lett. 2016;26:1983-1987.

76 Li Q, Wu Z. Isolation and identification of alkaloids from Fritillaria anhuensis S. C. Chen et S. F. Yin. Acta Pharmaceutica Sinica. 1986;21:767-771.

77 Pi HF, Ruan HL, Zhan YH, Wu JZ. Two new steroidal alkaloids from bulbs of *Fritillaria lichuanensis*. J. Asian Nat. Prod. Res. 2006;8:253-257.

78 Zhang J, Lao A, Xu R. Studies on chemical constituents of Fritillaria yuminensis. Acta Botanica Sinica. 1993;35:963-967.

79 Yan Z, Lu Y, Ding W, Chen Z. Studies on the chemical constituents of Fritillaria cirrhosa D. Don. Acta Univertitatis Medicinalis Secondae Shanghai. 1999;19:487-489.

80 Hong S, Kim Y, Kwon Y, Kim C. Alkaloid compounds of the bulbus of *Fritillaria walujewii*. Kor. J. Pharmacogn. 1998;29:104-109.

81 Liu M, Xu W, Xu C, Chen D, Wang J. Two new steroidal alkaloids from bulbs of Fritillaria pallidiflora. Chinese Traditional and Herbal Drugs. 2016;47:876-880.

82 Kitamura Y, Nishizawa M, Kaneko K, Ikura M, Hikichi K, Shiro M, et al. New steroidal alkaloids having a novel seven ring skeleton from *Fritillaria ussuriensis* Maxim. Tetrahedron. 1989;45:5755-5766.

83 Pi H, Zhang P, Ruan H, Zhang Y, Sun H, Wu J. Two new triterpenoids from the leaves and stems of *Fritillaria hupehensis*. J. Asian Nat. Prod. Res. 2009;11:779-782.

84 Pi H, Zhang P, Zhu T, Ruan H, Zhang Y, Sun H, et al. A new cycloartane triterpenoid from the leaves and stems of *Fritillaria hupehensis*. Chinese Chem. Lett. 2007;18:418-420.

85 Wu J, Ruan H, Zeng C, Cheng H, Zhang F, Zhao Q, et al. Structures of two new diterpenoid dimers from bulbs of *Fritillaria ebeiensis*. J. Asian Nat. Prod. Res. 1999;1:251-257.

86 Wu J, Morizan C, Iida A, Ueda S, Zhou Z, Xu M, et al. Structures of three new diterpenoids, fritillebic acid and fritillebins A and B, from bulbs of *Fritillaria ebeiensis* G. D. Yu et G. Q. Ji. Chem. Pharm. Bull. 1995;43:1448-1453.

87 Wu J, Ruan H, Yao N, Zhao Q, Sun H, Morizane C, et al. Structures of two diterpenoid dimers from bulbs of *Fritillaria ebeiensis*. J. Asian Nat. Prod. Res. 2000;2:161-167.

88 Atta-ur-Rahman, Akhtar MN, Choudhary MI, Tsuda Y, Yasin A, Sener B, et al. New diterpene isopimara-7,15-dien-19-oic acid and its prolyl endopeptidase inhibitory activity. Nat. Prod. Res. 2005;19:13-22.

89 Kang L, Zhou J, Shen Z. A new diterpenoid from *Fritillaria anhuiensis*. Acta Pharmaceutica Sinica. 2007;42:58-60.

90 Ruan H, Wu J, Deng S, Ma Z, Liu G, Chen H, et al. Stuctural elucidation of a new diterpenoid, fritillebinol from Ebeiensis Fritillary Bulb (Fritillaria ebeiensis). Chinese Traditional and Herbal Drugs. 1999;30:404-407.

91 Wu J, Pu Q, Jiang H, Jin B. Research of chemical components of Fritillaria genus plants Ⅶ. isolation and authentication of non-alkaloid components. Chinese Traditional and Herbal Drugs. 1989;20:4-6.

92 Shou Q, Tan Q, Shen Z. A novel sulfur-containing diterpenoid from *Fritillaria anhuiensis*. Tetrahedron Lett. 2009;50:4185-4187.

93 Ruan H, Zhang Y, Wu J, Deng S, Sun H, Fujita T. Structure of a novel diterpenoid ester, fritillahupehin from bulbs of *Fritillaria hupehensis* Hsiao and K.C. Hsia. Fitoterapia. 2002;73:288-291.

94 Zhang Y, Ruan H, Pi H, Wu J, Sun H, Fujita T. Structural elucidation of fritillahupehin from bulbs of *Fritillaria hupehensis* Hsiaoet K.C. Hsia. J. Asian Nat. Prod. Res. 2004;6:29-34.

95 Ruan H, Zhang Y, Wu J, Sun H, Fujita T. Two new diterpenoid dimers, fritillebinide D and E, from bulbs of *Fritillaria ebeiensis*. J. Asian Nat. Prod. Res. 2002;4:309-314.

96 Xu J, Guo P, Liu C, Sun Z, Gui L, Guo Y, et al. Neuroprotective kaurane diterpenes from *Fritillaria ebeiensis*. Bioscience, biotechnology, and biochemistry. 2011;75:1386-1388.

97 Park JE, Lee SY, Woo KW, Lee JH, Lee KR. Two new *ent*-kaurane diterpenoids from the roots of *Fritillaria thunbergii*. Bulletin of the Korean Chemical Society. 2013;34:1589-1591.

98 Wu J, Wen Y, Ruan H, Yao N, Zhao Q, Sun H. Structure of a new chloro-substituded diterpenoid, fritillaziebinol from Zihua Ebei Beimu Bulbs (Fritillaria ebeiensis var. purpurea). Chinese Traditional and Herbal Drugs. 1999;30:804-807.

99 Shen S, Chen C, Bu R, Ga L, Li G, Tan Y, et al. Three new steroidal saponins from *Fritillaria pallidiflora*. J. Asian Nat. Prod. Res. 2011;13:1014-1022.

100 Shen S, Li G, Huang J, Chen C, Ren B, Lu G, et al. Steroidal saponins from *Fritillaria pallidiflora* Schrenk. Fitoterapia. 2012;83:785-794.

101 Shen S, Li G, Huang J, Tan Y, Chen C, Ren B, et al. Chemical constituents from *Fritillaria pallidiflora* Schrenk. Biochem. Syst. Ecol. 2012;45:183-187.

102 Li W, Bu R, Chen C, Yu T, Wang J. Isolation and identification of non-alkaloid constituents from Fritillaria pallidiflora Schrenk. Modern Chinese Medicine. 2013;15:175-177.

103 Yue-xian J, Wei-qin P, Hong-yan W, Guo-xu M, Lei-ling S, Jing Z. Chemical constituents from plant of Fritillaria palldiflora. Chinese Traditional and Herbal Drugs. 2019;50:2534-2538.

104 Ying-Ma, Gao T, Wang J, Tian L, Yun-Zhu, Cheng S, et al. Chemical constituents of the flowers of *Fritillaria pallidiflora*. Chem. Nat. Compd.+. 2016;52:309-310.

105 Jin X, Xu D, Xu Y, Cui D, Xiao Y, Tian Z, et al. The structure identification of zhebeiresinol. Acta Pharmaceutica Sinica. 1993;28:212-215.

106 Wei P, Ting H, Qing-Chun L, Lu-Ping Q. Chemical constituents of the flower of *Fritillaria thunbergii*. Chem. Nat. Compd.+. 2012;48:491-492.

107 Wang X, Li Y. Analysis of volatile oil of *Fritillaria cirrhosa* D. Don by GC-MS. Asian J. Chem. 2013;25:3252-3254.

108 Liu J, Peng C, He C, Liu J, He Y, Guo L, et al. New amino butenolides from the bulbs of *Fritillaria unibracteata*. Fitoterapia. 2014;98:53-58.

109 Zhou J, Jiang Y, Bi Z, Li P. Study on nucleosides from Fritillaria puqiensis. Chinese Pharmaceutical Journal. 2008;43:894-896.

110 Cui D, Yan M, Wang S, Zhang X, Xu D. Studies on chemcial constituents of stems and leaves of Fritillaria ussuriensis Maxim. China Journal of Chinese Materia Medica. 1995;20:298-320.

111 Wang D, Zhu J, Wang S, Wang X, Ou Y, Wei D, et al. Antitussive, expectorant and anti-inflammatory alkaloids from bulbus *Fritillariae cirrhosae*. Fitoterapia. 2011;82:1290-1294.

112 Chen S, Li P, Kwan Y, Lin G. In vitro tracheobronchial relaxation of Fritillaria alkaloids. Chinese Journal of Natural Medicines. 2011;9:345-353.

113 Wang D, Wang S, Chen X, Xu X, Zhu J, Nie L, et al. Antitussive, expectorant and anti-inflammatory activities of four alkaloids isolated from bulbus of *Fritillaria wabuensis*. J. Ethnopharmacol. 2012;139:189-193.

114 Xu Y, Ming TW, Gaun TKW, Wang S, Ye B. A comparative assessment of acute oral toxicity and traditional pharmacological activities between extracts of *Fritillaria cirrhosae* Bulbus and *Fritillaria pallidiflora* Bulbus. J. Ethnopharmacol. 2019;238:111853.

115 Wu X, Chan S, Ma J, Li P, Shaw P, Lin G. Investigation of association of chemical profiles with the tracheobronchial relaxant activity of Chinese medicinal herb Beimu derived from various *Fritillaria* species. J. Ethnopharmacol. 2018;210:39-46.

116 Gilani AH, Shaheen F, Christopoulos A, Mitchelson F. Interaction of ebeinone, an alkaloid from *Fritillaria imperialis*, at two muscarinic acetylcholine receptor subtypes. Life Sci. 1997;60:535-544.

117 Lin B, Ji H, Li P, Jiang Y, Fang W. Selective antagonism activity of alkaloids from bulbs *Fritillariae* at muscarinic receptors: functional studies. Eur. J. Pharmacol. 2006;551:125-130.

118 Zhou Y, Ji H, Lin B, Jiang Y, Li P. The effects of five alkaloids from bulbus *Fritillariae* on the concentration of cAMP in HEK cells transfected with muscarinic M_2_ receptor plasmid. Am. J. Chin. Med. 2006;34:901-910.

119 Yeum H, Lee Y, Kim S, Roh S, Lee J, Seo Y. *Fritillaria cirrhosa*, *Anemarrhena asphodeloides*, Lee-Mo-Tang and cyclosporine a inhibit ovalbumin-induced eosinophil accumulation and Th2-mediated bronchial hyperresponsiveness in a murine model of asthma. Basic Clin Pharmacol Toxicol. 2007;100:205-213.

120 Wang D, Du Q, Li H, Wang S. The isosteroid alkaloid imperialine from bulbs of *Fritillaria cirrhosa* mitigates pulmonary functional and structural impairment and suppresses inflammatory response in a COPD-like rat model. Mediat. Inflamm. 2016;2016:1-17.

121 Kim EJ, Yoon YP, Woo KW, Kim J, Min SY, Lee HJ, et al. Verticine, ebeiedine and suchengbeisine isolated from the bulbs of Fritillaria thunbergii Miq. inhibited the gene expression and production of MUC5AC mucin from human airway epithelial cells. Phytomedicine. 2016;23:95-104.

122 Pae H, Oh H, Choi B, Oh G, Paik S, Jeong S, et al. Differentiation-inducing effects of verticinone, an isosteroidal alkaloid isolated from the bulbus of *Fritillaria ussuriensis*, on human promyelocytic leukemia HL-60 cells. Biol. Pharm. Bull. 2002;25:1409-1411.

123 Yun YG, Jeon BH, Lee JH, Lee SK, Lee HJ, Jung KH, et al. Verticinone induces cell cycle arrest and apoptosis in immortalized and malignant human oral keratinocytes. Phytother. Res. 2008;22:416-423.

124 Kavandi L, Lee LR, Bokhari AA, Pirog JE, Jiang Y, Ahmad KA, et al. The Chinese herbs *Scutellaria baicalensis* and *Fritillaria cirrhosa* target NFκB to inhibit proliferation of ovarian and endometrial cancer cells. Mol. Carcinog. 2015;54:368-378.

125 Wang D, Jiang Y, Wu K, Wang S, Wang Y. Evaluation of antitumor property of extracts and steroidal alkaloids from the cultivated Bulbus *Fritillariae ussuriensis* and preliminary investigation of its mechanism of action. BMC Complement Altern Med. 2015;15:29.

126 Bokhari AA, Syed V. Inhibition of transforming growth factor-β (TGF-β) signaling by *Scutellaria baicalensis* and *Fritillaria cirrhosa* extracts in endometrial cancer. J. Cell. Biochem. 2015;116:1797-1805.

127 Abu N, Yeap SK, Pauzi AZM, Akhtar MN, Zamberi NR, Ismail J, et al. Dual regulation of cell death and cell survival upon induction of cellular stress by isopimara-7,15-dien-19-oic Acid in cervical cancer, heLa cells in vitro. Front. Pharmacol. 2016;7:89.

128 Zhi Z, Qinsi H, Liting X, Wenhao C, Hua B, Zhe Z, et al. The peiminine stimulating autophagy in human colorectal carcinoma cells via AMPK pathway by SQSTM1. Open Life Sciences. 2016;11:358-366.

129 Zhao B, Shen C, Zheng Z, Wang X, Zhao W, Chen X, et al. Peiminine inhibits glioblastoma in vitro and in vivo through cell cycle arrest and autophagic flux blocking. Cell. Physiol. Biochem. 2018;51:1566-1583.

130 Lin Q, Qu M, Patra HK, He S, Wang L, Hu X, et al. Mechanistic and therapeutic study of novel anti-tumor function of natural compound imperialine for treating non-small cell lung cancer. J. Ethnopharmacol. 2020;247:112283.

131 Tan H, Zhang G, Yang X, Jing T, Shen D, Wang X. Peimine inhibits the growth and motility of prostate cancer cells and induces apoptosis by disruption of intracellular calcium homeostasis through Ca^2+^ /CaMKII/JNK pathway. J. Cell. Biochem. 2019;121:81-92.

132 Wu K, Mo C, Xiao H, Jiang Y, Ye B, Wang S. Imperialine and verticinone from bulbs of *Fritillaria wabuensis* inhibit pro-inflammatory mediators in LPS-stimulated RAW 264.7 macrophages. Planta Med. 2015;81:821-829.

133 Zhou M, Ma X, Ding G, Wang Z, Liu D, Tong Y, et al. Comparison and evaluation of antimuscarinic and anti-inflammatory effects of five Bulbus Fritillariae species based on UPLC-Q/TOF integrated dual-luciferase reporter assay, PCA and ANN analysis. Journal of Chromatography B. 2017;1041-1042:60-69.

134 Wang D, Yang J, Du Q, Li H, Wang S. The total alkaloid fraction of bulbs of *Fritillaria cirrhosa* displays anti-inflammatory activity and attenuates acute lung injury. J. Ethnopharmacol. 2016;193:150-158.

135 Fan B, Li T, Xu S, Chen L, Wei G, Qian C. Efficient, accurate and comprehensive evaluation of polysaccharides from *Fritillaria* and their inhibitory responses to mouse inflammation. Food Funct. 2019;10:7913-7925.

136 Chen K, Lv ZT, Zhou CH, Liang S, Huang W, Wang ZG, et al. Peimine suppresses interleukin‑1β‑induced inflammation via MAPK downregulation in chondrocytes. Int. J. Mol. Med. 2019;43:2241-2251.

137 Luo Z, Zheng B, Jiang B, Xue X, Xue E, Zhou Y. Peiminine inhibits the IL-1β induced inflammatory response in mouse articular chondrocytes and ameliorates murine osteoarthritis. Food Funct. 2019;10:2198-2208.

138 Liu S, Yang T, Ming TW, Gaun TKW, Zhou T, Wang S, et al. Isosteroid alkaloids with different chemical structures from Fritillariae Cirrhosae Bulbus alleviate LPS-induced inflammatory response in RAW 264.7 cells by MAPK signaling pathway. Int. Immunopharmacol. 2020;78:106047.

139 Kang DG, Oh H, Cho DK, Kwon EK, Han JH, Lee HS. Effects of bulb of *Fritillaria ussuriensis* Maxim. on angiotensin converting enzyme and vascular release of NO/cGMP in rats. J. Ethnopharmacol. 2002;81:49-55.

140 Oh H, Kang DG, Lee S, Lee Y, Lee HS. Angiotensin converting enzyme (ACE) inhibitory alkaloids from *Fritillaria ussuriensis*. Planta Med. 2003;69:564-565.

141 Kang DG, Sohn EJ, Lee YM, Lee AS, Han JH, Kim TY, et al. Effects of bulbus *Fritillaria* water extract on blood pressure and renal functions in the L-NAME-induced hypertensive rats. J. Ethnopharmacol. 2004;91:51-56.

142 Lin B, Ji H, Li P, Fang W, Jiang Y. Inhibitors of acetylcholine esterasein vitro-screening of steroidal alkaloids from *Fritillaria* species. Planta Med. 2006;72:814-818.

143 Kim S, Shin D, Oh M, Chung S, Lee J, Chang I, et al. Inhibition of sortase, a bacterial surface protein anchoring transpeptidase, by β-itosterol-3-O-glucopyranoside from *Fritillaria verticillata*. Bioscience, Biotechnology, and Biochemistry. 2003;67:2477-2479.

144 Li Y, Xu C, Zhang Q, Liu JY, Tan RX. *In vitro* anti-gelicobacter pylori action of 30 Chinese herbal medicines used to treat ulcer diseases. J. Ethnopharmacol. 2005;98:329-333.

145 Kim M, Nguyen D, Heo Y, Park KH, Paik H, Kim YB. Antiviral activity of *Fritillaria thunbergii* extract against human influenza virus H1N1 (PR8) *in vitro*, *in* *ovo* and *in vivo*. J. Microbiol. Biotechnol. 2020;30:172-177.

146 Li X, Gao W, Huang L, Huang L, Liu C. In vitro antioxidant and in vivo anti-inflammatory potential of crude non-alkaloid fractions from *Fritillaria ussuriensis* Maxim. Lat. Am. J. Pharm. 2010;8:1328-1335.

147 Ruan X, Yang L, Cui W, Zhang M, Li Z, Liu B, et al. Optimization of supercritical fluid extraction of total alkaloids, peimisine, peimine and peiminine from the bulb of *Fritillaria thunbergii* Miq, and evaluation of antioxidant activities of the extracts. Materials. 2016;9:524.

148 Pan F, Su T, Liu Y, Hou K, Chen C, Wu W. Extraction, purification and antioxidation of a polysaccharide from *Fritillaria unibracteata* var*. wabuensis*. Int. J. Biol. Macromol. 2018;112:1073-1083.

149 Rozi P, Abuduwaili A, Mutailifu P, Gao Y, Rakhmanberdieva R, Aisa HA, et al. Sequential extraction, characterization and antioxidant activity of polysaccharides from *Fritillaria pallidiflora* Schrenk. Int. J. Biol. Macromol. 2019;131:97-106.

150 Liu S, Yang T, Ming TW, Gaun TKW, Zhou T, Wang S, et al. Isosteroid alkaloids from *Fritillaria cirrhosa* bulbus as inhibitors of cigarette smoke-induced oxidative stress. Fitoterapia. 2020;140:104434.

151 Xu F, Xu S, Wang L, Chen C, Zhou X, Lu Y, et al. Antinociceptive efficacy of verticinone in murine models of inflammatory pain and paclitaxel induced neuropathic pain. Biol. Pharm. Bull. 2011;34:1377-1382.

152 Xu J, Zhao W, Pan L, Zhang A, Chen Q, Xu K, et al. Peimine, a main active ingredient of *Fritillaria*, exhibits anti-inflammatory and pain suppression properties at the cellular level. Fitoterapia. 2016;111:1-6.

153 Cho I, Lee MJ, Kim J, Han NY, Shin KW, Sohn Y, et al. *Fritillaria ussuriensis* extract inhibits the production of inflammatory cytokine and MAPKs in mast cells. Bioscience, Biotechnology, and Biochemistry. 2011;75:1440-1445.

154 Lee B, Kim E, Kim J, Min J, Jeong D, Jun J, et al. Antiallergic effects of peiminine through the regulation of inflammatory mediators in HMC-1 cells. Immunopharm. Immunot. 2015;37:351-358.

155 Xu J, Guo P, Liu C, Sun Z, Gui L, Guo Y, et al. Neuroprotective kaurane diterpenes from *Fritillaria ebeiensis*. Bioscience, Biotechnology, and Biochemistry. 2011;75:1386-1388.

156 Chen G, Liu J, Jiang L, Ran X, He D, Li Y, et al. Peiminine Protects Dopaminergic Neurons from Inflammation-Induced Cell Death by Inhibiting the ERK1/2 and NF-κB Signalling Pathways. IJMS. 2018;19:821.

157 Boojar FMA, Aghaei R, Mashhadi Akbar Boojar M. Data on possible in vitro anti-diabetic effects of verticinone on β-TC6 pancreatic and C2C12 skeletal muscle cells. Data in Brief. 2020;28:104828.
